# Cortical development dynamics across autism spectrum disorder mouse models

**DOI:** 10.1038/s41586-026-10679-1

**Published:** 2026-06-17

**Authors:** Lena A. Schwarz, Christoph P. Dotter, Sergey Isaev, Michela Lisi, Daniel Malzl, Christoph Büschl, Sabrina Ladstätter, Bárbara Oliveira, Matteo Barel, Bernadette Basilico, Chaitanya Chintaluri, Sarah Gorkiewicz, Mohammad Goudarzi, Tereza Belinova, Stephan Reichl, Gintarė Sendžikaitė, Satish Arcot Jayaram, Peter Koppensteiner, Christoph Sommer, Tim P. Vogels, Jörg Menche, Igor Adameyko, Peter V. Kharchenko, Christoph Bock, Gaia Novarino

**Affiliations:** 1 Institute of Science and Technology Austria, Klosterneuburg, Austria; 2 Department of Neuroimmunology, Center for Brain Research, Medical University Vienna, Vienna, Austria; 3 Ludwig Boltzmann Institute for Network Medicine, University of Vienna, Vienna, Austria; 4 Max Perutz Labs, Vienna Biocenter Campus (VBC), Vienna, Austria; 5 Department of Structural and Computational Biology, Center for Molecular Biology, University of Vienna, Vienna, Austria; 6 CeMM Research Center for Molecular Medicine of the Austrian Academy of Sciences, Vienna, Austria; 7 Institute of Artificial Intelligence, Center for Medical Data Science, Medical University of Vienna, Vienna, Austria; 8 Faculty of Mathematics, University of Vienna, Vienna, Austria; 9 Department of Physiology and Pharmacology, Karolinska Institute, Stockholm, Sweden

**Keywords:** Neuroscience, Molecular biology

## Abstract

Despite the functional diversity of over 100 causal genes^1–3^, phenotypic convergence across models may reveal common neurobiological processes in autism spectrum disorder (ASD). Here we profiled 251 samples from 11 monogenic mouse models of ASD using single-nucleus multi-omic sequencing across three developmental stages, both sexes and two brain regions. Despite genetic heterogeneity, ASD-linked mutations converged on perturbations of the radial glial cell lineage. These alterations reflect a transient developmental delay rather than lasting lineage misspecification and resolve by postnatal stages. Molecularly, the largest transcriptional differences emerged in neurons at early postnatal stages. These changes included downregulation of synaptic and ion channel-related genes, consistent with homeostatic adaptation or delayed maturation. Network analysis showed molecular convergence across models within each developmental stage, suggesting that diverse mutations linked to ASD impinge on common, stage-specific processes. Convergence becomes less pronounced by postnatal day 14, highlighting the dynamic nature of ASD-associated changes. Cross-genotype heterogeneity is superimposed on stage-specific effects. Electrophysiology corroborated this pattern: mutants generally showed altered neuronal excitability and synaptic properties with model-specific nuances. Our study also highlighted sex-specific gene expression alterations, with female mice often displaying larger effect sizes than male mice. Together, our findings provide a comprehensive view of developmental cellular and molecular dynamics across models of ASD.

## Abstract (web summary)

Using single-nucleus multi-omic sequencing, diverse autism spectrum disorder-linked gene mutations converge on transient, stage-specific disruptions in early brain development, and highlight sex-specific gene expression alterations.

## Main

Several studies have uncovered the heterogeneous genetic landscape of ASD, identifying numerous monogenic forms^4,5^. Although these discoveries are crucial for understanding ASD biology, fundamental questions remain: how mutations that are scattered across many genes lead to similar disorders; and how similar these genetically defined ASDs are to one another.

**Fig. 1:**
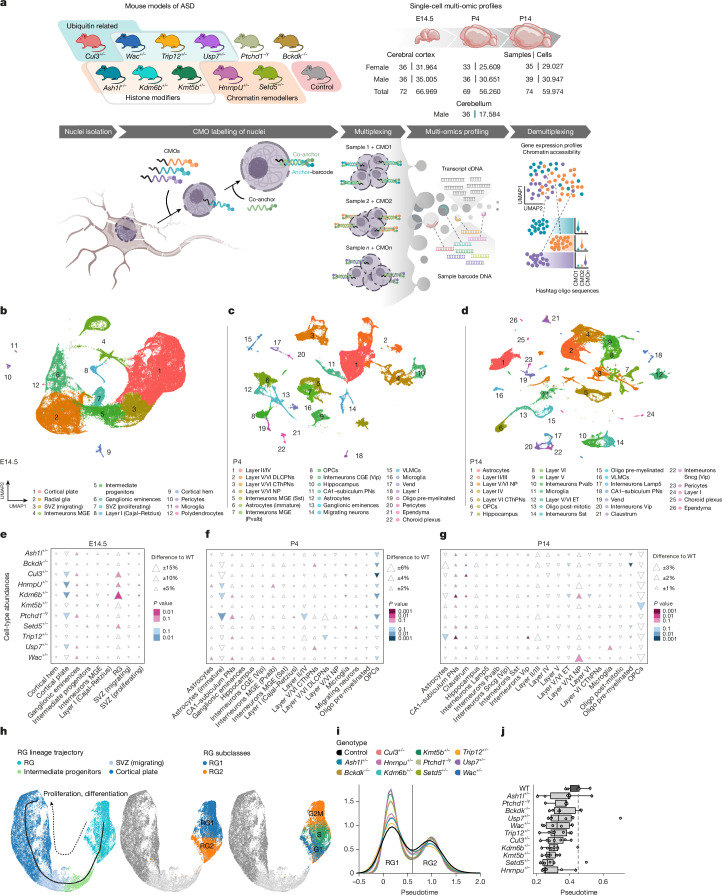
Large-scale multi-omics profiling of high-risk genes associated with ASD. **a**, Simultaneous transcriptome and chromatin accessibility profiling of brain samples from 11 monogenic mouse lines, each carrying a mutation in a high-risk gene linked to ASD. An overview of the experimental design is shown, indicating total samples and cells analysed per time point. Illustration of the multi-omics approach created by J. Kirchner. **b**–**d**, Uniform manifold approximation and projection (UMAP) visualization of integrated snRNA-seq and snATAC-seq data from E15.4 (**b**), P4 (**c**) and P14 (**d**). Cell clusters are coloured and numbered by cell type. ET, extratelencephalic-projecting neurons; NP, near-projecting; PN, projection neuron; SVZ, subventricular zone; Vend, vascular endothelial cells; VLMC, vascular leptomeningeal cell. **e**–**g**, Changes in cell-type abundances in individual mutants linked to ASD from E14.5 (**e**), P4 (**f**) and P14 (**g**). The arrowheads indicate relative percentage differences compared with WT. Statistical significance was assessed in edgeR using a two-tailed quasi-likelihood *F*-test. Nominal *P* values are shown. **h**, Pseudotime analysis of the RG lineage at E14.5. The solid line indicates the trajectory inferred from gene expression embedding (Methods). The dashed arrow indicates the trajectory from the RG (root) towards cortical plate cells (left). Two RG subpopulations were distinguished: RG1 (G1/S phase) and RG2 (G2 phase) (centre and right). **i**, Pseudotime analysis of RG cells indicating that mutants linked to ASD tend to remain in the proliferating RG1 subpopulation. The vertical line marks the boundary between two modes of the pseudotime distribution. **j**, Median pseudotime analysis of RG cells indicates a consistent developmental delay in mutants linked with ASD compared with WT. The centre line represents the median, the box limits show the 25–75% interquartile range (IQR), the whiskers extend to the furthest data points within 1.5× the IQR from the box edges, and the data points outside this range are represented as dots. The dashed line indicates the median of the WT per-sample averages. Animals used for analyses: *n* (E14.5) = 7 (*Cul3*^*+/−*^,* Kdm6b*^*+/−*^ and *Trip12*^*+/−*^), 6 (*Ash1l*^*+/−*^, *Bckdk*^*−/−*^, *HnrnpU*^*+/−*^, *Kmt5b*^*+/−*^, *Setd5*^*+/−*^, *Usp7*^*+/−*^, *Wac*^*+/−*^ and colony-matched controls) and 3 (*Ptchd1*^*−/y*^); *n* (P4) = 6 (*Ash1l*^*+/−*^,* Bckdk*^*−/−*^, *Cul3*^*+/−*^, *HnrnpU*^*+/−*^, *Kdm6b*^*+/−*^, *Setd5*^*+/−*^, *Trip12*^*+/−*^, *Usp7*^*+/−*^, *Wac*^*+/−*^ and colony-matched controls), 4 (*Kmt5b*^*+/−*^) and 3 (*Ptchd1*^*−/y*^); *n* (P14) = 8 (*Ash1l*^*+/−*^ and* Cul3*^*+/−*^), 7 (colony-matched controls), 6 *(Bckdk*^*−/−*^,* HnrnpU*^*+/−*^,* Kdm6b*^*+/−*^,* Kmt5b*^*+/−*^, *Setd5*^*+/−*^, *Trip12*^*+/−*^, *Usp7*^*+/−*^ and *Wac*^*+/−*^) and 3 (*Ptchd1*^*−/y*^).

Here we used single-nucleus multi-omics sequencing to study 251 samples from mouse models of high-risk genes linked to ASD, spanning developmental stages, sexes and two brain regions (Fig. 1a). These data comprehensively represent cell states across ASD-associated mutants, uncovering converging as well as diverging phenotypes.

## Single-nucleus multi-omics profiling

Droplet-based single-cell multi-omic technologies enable matched gene expression and chromatin accessibility profiling in thousands of single cells in one experiment. However, existing methods remain costly and limited in scalability. Here we optimized a multiplexing strategy for combined single-nucleus RNA sequencing (snRNA-seq) and single-nucleus assay for transposase-accessible chromatin using sequencing (snATAC-seq). We utilized a multiplexing approach using cholesterol-modified oligos (CMOs)^6^ as sample-specific barcodes (Fig. 1a and Supplementary Table 1), enabling pooled processing. In this way, we profiled 45 conditions comprising 251 samples (Supplementary Table 1), including mutants and controls, allowing us to assess transcriptional and chromatin states at single-cell resolution across a broad range of genetic ASD mouse models (Fig. 1a; Methods).

In total, we collected the molecular profiles of 200,787 nuclei (66,969 at embryonic day 14.5 (E14.5), 56,260 at postnatal day 4 (P4) and 59,974 at P14 for the forebrain; 17,584 at P4 for the cerebellum). We detected an average of approximately 3,900 transcripts, about 2,000 genes and approximately 4,200 fragments per cell at a median sequencing depth of about 25,000 reads per cell (Supplementary Figs. 1d and 3a), aligning with standard recommendations for these types of analyses.

**Extended Data Fig. 1:**
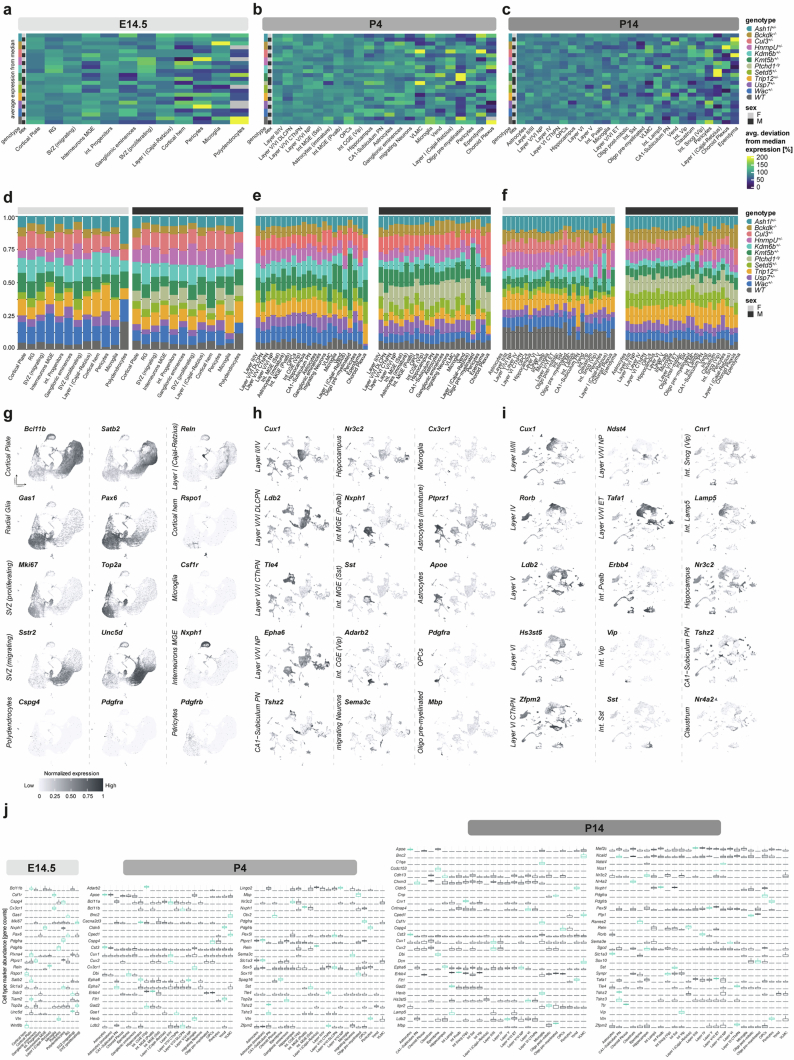
Cell type classifications and distribution across time points. **a-c**, Average deviation from the median expression of individual cell types of the ASD mutants across time points. Each tile in the heatmap shows the average expression deviation of each replicate relative to the median expression of all samples per cell type expressed as a percentage. **d-f**, Relative proportion of cell types by genotype, time point, and sex. **g-i**, UMAP visualization of cells collected at each time point, showing expression levels of canonical molecular markers. **j**, Box plots show marker abundance of selected cell type markers across time points. Center lines represent medians, the box limits show the 25-75% interquartile range (IQR), whiskers extend to the furthest data points within 1.5 × IQR from the box edges. Animals used for analyses: *n (E14.5)* = 7 *(Cul3*^*+/*−^*, Kdm6b*^*+/*−^*, Trip12*^*+/*−^*)*, 6 *(Ash1l*^*+/*−^*, Bckdk*^−*/*−^*, HnrnpU*^*+/*−^*, Kmt5b*^*+/*−^*, Setd5*^*+/*−^*, Usp7*^*+/*−^*, Wac*^*+/*−^, colony-matched controls), 3 (*Ptchd1*^*−/y*^); *n (P4)* = 6 (*Ash1l*^*+/*−^*, Bckdk*^−*/*−^*, Cul3*^*+/*−^*, HnrnpU*^*+/*−^*, Kdm6b*^*+/*−^*, Setd5*^*+/*−^*, Trip12*^*+/*−^*, Usp7*^*+/*−^*, Wac*^*+/*−^, colony-matched controls), 4 (*Kmt5b*^*+/*−^), 3 (*Ptchd1*^*−/y*^); *n (P14)* = 8 (*Ash1l*^*+/*−^*, Cul3*^*+/*−^), 7 (colony-matched controls), *6 (Bckdk*^−*/*−^*, HnrnpU*^*+/*−^*, Kdm6b*^*+/*−^*, Kmt5b*^*+/*−^*, Setd5*^*+/*−^*, Trip12*^*+/*−^*, Usp7*^*+/*−^*, Wac*^*+/*−^*)*, 3 (*Ptchd1*^*−/y*^).

We identified 12 (E14.5), 22 (P4), 26 (P14; cortex) and 16 (P4; cerebellum) major cell types (Figs. 1b–d and 6a; Methods). All samples and genotypes were similarly distributed across clusters (Extended Data Fig. 1a–f and Supplementary Table 1). Each cluster was annotated based on canonical marker gene expression and established mouse brain cell-type classifications (Extended Data Fig. 1g–j).

At E14.5 (Fig. 1b), the cell types comprised primarily cortical plate neurons (expressing *Bcl11b* and *Satb2*), radial glial (RG) cells (*Pax6* and *Gas1*) and intermediate progenitors (*Unc5d*), with a small fraction of medial ganglionic eminence (MGE)-derived interneurons (*Nxph1*). At postnatal stages (Fig. 1c,d), we differentiated between upper (layers II–IV and layer II/III; *Cux1*, *Cux2* and *Bcl11a*) and lower (layer V/VI deep-layer cortical projection neurons (DLCPN); *Bcl11b*, *Ldb2* and *Pex5l*) cortical layers, including corticothalamic projection (layer V/VI corticothalamic projection neuron (CThPN) and layers IV–VI; *Gse1*, *Tle4* and *Zfpm2*) and near-projecting neurons (layer V/VI; *Epha6* and *Lingo2*). As samples included the hippocampus, we distinguished between hippocampal cells (*Cacna2d3*, *Nr3c2* and *Epha6*) and adjacent CA1–subiculum projection neurons (*Tshz2* and *Tshz3*). Glial cells were first observed at P4, including oligodendrocyte precursor cells (OPCs; *Pdgfra* and *Cspg4*) and their maturation stages (*Itpr2* and *Plp2* for postmitotic; and *Mpb* for pre-myelinated), astrocytes (*Ptprz1*, *Slc1a3* and *Apoe*) and microglia (*Hexb*, *Cx3cr1* and *Csf1r*).

Consistent with the timely cortical positioning of interneurons, we identified several interneuron populations from P4 onwards, including caudal ganglionic eminence (Int. CGE (Vip) and Int. Sncg (Vip); *Adarb2*, *Gad2*, *Vip* and *Synpr*) and MGE-derived interneurons (Int. MGE (Pvalb), Int. Sst and Int. Lamp5; *Erbb4*, *Lamp5*, *Nxph1* and *Sst*).

Our dataset thus provides a comprehensive view of the cellular landscape of the developing mouse cerebral cortex (Fig. 1b–d), consistent with previous reports^7–10^.

## RG progression defects in mutants linked to ASD

**Extended Data Fig. 2:**
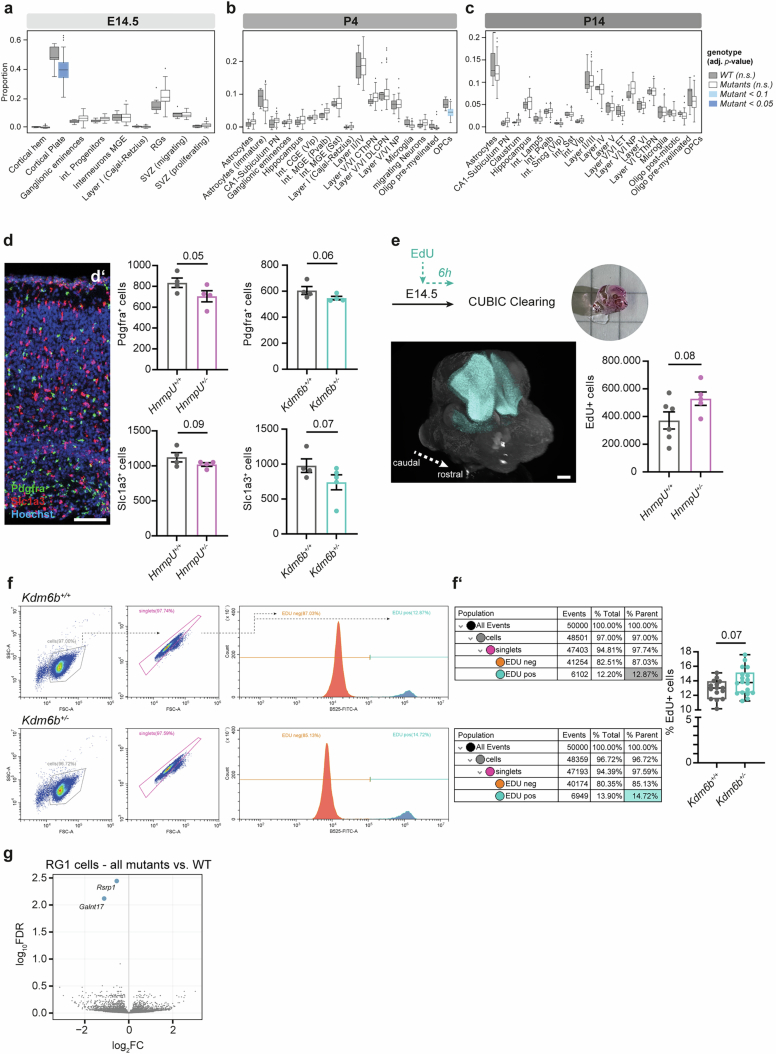
Quantification of cell-type abundance by snRNA-seq, in situ labeling, cytometry and EdU labeling. **a-c**, scCODA analysis from individual time points quantifying compositional cell type changes in the aggregated ASD mutants compared to colony-matched controls. Center lines represent medians, the box limits show the 25−75% interquartile range (IQR), whiskers extend to the furthest data points within 1.5 × IQR from the box edges, and data points outside this range are represented as dots. Animals used for analyses: *n (E14.5)* = 7 *(Cul3*^*+/*−^*, Kdm6b*^*+/*−^*, Trip12*^*+/*−^*)*, 6 *(Ash1l*^*+/*−^*, Bckdk*^−*/*−^*, HnrnpU*^*+/*−^*, Kmt5b*^*+/*−^*, Setd5*^*+/*−^*, Usp7*^*+/*−^*, Wac*^*+/*−^, colony-matched controls), 3 (*Ptchd1*^*−/y*^); *n (P4)* = 6 (*Ash1l*^*+/*−^*, Bckdk*^−*/*−^*, Cul3*^*+/*−^*, HnrnpU*^*+/*−^*, Kdm6b*^*+/*−^*, Setd5*^*+/*−^*, Trip12*^*+/*−^*, Usp7*^*+/*−^*, Wac*^*+/*−^, colony-matched controls), 4 (*Kmt5b*^*+/*−^), 3 (*Ptchd1*^*−/y*^); *n (P14)* = 8 (*Ash1l*^*+/*−^*, Cul3*^*+/*−^), 7 (colony-matched controls), *6 (Bckdk*^−*/*−^*, HnrnpU*^*+/*−^*, Kdm6b*^*+/*−^*, Kmt5b*^*+/*−^*, Setd5*^*+/*−^*, Trip12*^*+/*−^*, Usp7*^*+/*−^*, Wac*^*+/*−^*)*, 3 (*Ptchd1*^*−/y*^). **d**, In situ hybridization for *Pdgfra* (OPCs; green), *Slc1a3* (immature astrocytes; red) and Hoechst (nuclear counterstain; blue) in *HnrnpU*^*+/*−^ and *Kdm6b*^*+/*−^ animals compared to their littermate controls (*n* = 4 animals per condition, at least 3 cortical slices/ animal were analyzed; unpaired one-tailed t-test; unpaired one-tailed Mann-Whitney test for *Pdgfra*^+^ cells in *Kdm6b*^*+/*−^). **d’**, Representative image of in situ hybridization. **e**, EdU-labeling combined with CUBIC clearing showing increased numbers of proliferative cells in *HnrnpU*^+/−^ animals (*n* = 6 (*HnrnpU*^*+/+*^), 5 (*HnrnpU*^*+/*−^), unpaired two-tailed t-test). **f**, Representative cell gates and EdU populations of *Kdm6b* mutant and littermate controls. Sequential gating was performed according to a defined population hierarchy. **f’** EdU-labeling combined with cytometry analysis showing increased numbers of proliferative cells in *Kdm6b*^+/−^ animals (*n* = 15 (*Kdm6b*^*+/+*^), 18 (*Kdm6b*^*+/*−^), unpaired two-tailed t-test). Center lines represent medians, the box limits show the 25-75% IQR, whiskers extend to the minimum and maximum values. **g**, Differential gene expression analysis between all mutants collectively and control RG1 cells highlights two significant downregulated genes, associated with cell cycle, neuronal maturation, and differentiation. Data are mean ± SEM in **d** and **e**. Scale bars: 100 µm in **d’**, 500 µm in **e**. Source data

To explore how mutations associated with ASD affect cortical development, we initially compared the relative proportion of each cell type in all mutants combined versus wild-type (WT) controls (Extended Data Fig. 2a–c). Overall proportions were similar; however, cortical plate cells and OPCs were significantly depleted in mutants at E14.5 and P4 (Extended Data Fig. 2a,b), respectively. Stratifying by genotype confirmed that these changes were consistent across mutants (Fig. 1e–g), with stronger trends in *Cul3*, *HnrnpU*, *Kdm6b* and *Ptchd1*. The reduction in cortical plate cells at E14.5 coincided with an increased proportion of RG cells (Fig. 1e). At P4, neuronal numbers were similar between mutants and WT; however, mutants displayed a reduction in the number of immature astrocytes and OPCs (Fig. 1f). These reductions were independently confirmed in two selected mutants, *HnrnpU*^*+/−*^ and *Kdm6b*^*+/−*^, by in situ hybridization for *Slc1a3* (immature astrocytes) and *Pdgfra* (OPCs; Extended Data Fig. 2d). These observations independently suggest a delay in the RG cell lineage progression, potentially affecting the gliogenic switch. This delay was pronounced in mutants exhibiting larger differences in RG and cortical plate cell numbers at E14.5, such as *Cul3*, *HnrnpU*, *Kdm6b*, *Ptchd1* and *Setd5* mutants. At P14, OPC numbers remained lower across mutants linked with ASD, whereas the number of other cell types had normalized (Fig. 1g).

To identify the source of these alterations, we investigated RG lineage progression by performing pseudotime analysis on the E14.5 dataset (Fig. 1h). By analysing the trajectories from RG cells towards cortical plate neurons, we identified two RG subpopulations (RG1 and RG2; Fig. 1h,i). RG1 cells were characterized by the expression of proliferation markers (for example, *MKi67*), indicating active cell division. Pseudotime analysis revealed that mutant RG cells tend to remain in the proliferating RG1 state (Fig. 1h–j) at the cost of delayed differentiation.

To validate alterations in RG cell proliferation, we performed EdU labelling at E14.5 in *Kdm6b*^*+/−*^ and *HnrnpU*^*+/−*^ embryos and WT littermates (Extended Data Fig. 2e,f′ and Supplementary Video 1). Consistent with the snRNA-seq results, both *HnrnpU*^*+/−*^ and *Kdm6b*^*+/−*^ mutants showed increased numbers of EdU-positive cells, indicative of prolonged proliferation and/or an expanded pool of proliferative RG cells at E14.5 (Extended Data Fig. 2e,f′).

We next analysed differentially expressed genes (DEGs) in RG1 cells shared across mutants by comparing all mutants, collectively, to WT RG1 cells. Only two genes, *Rsrp1* and *Galnt17*, were significantly downregulated (Extended Data Fig. 2g). *Rsrp1* encodes a protein implicated in splicing and cell cycle regulation^11^. *Galnt17* is an *N*-acetylgalactosaminyltransferase that has been linked to developmental delay and neural differentiation defects^12^. Although these two genes may represent biomarkers for neurodevelopmental delay, they are unlikely to fully account for the observed RG progression phenotype. The analyses of each mutant separately showed variable DEG numbers (false discovery rate (FDR) < 0.1) in RG1 cells, with some mutants showing few or no DEGs, suggesting that in some cases, delays may originate earlier during development. *HnrnpU* was the most affected (more than 150 DEGs; Supplementary Table 2). Although other mutants showed fewer DEGs, several DEGs were detected in multiple mutants. For example, *HnrnpU*, *Kdm6b* and *Kmt5b* mutants showed downregulation of *Setbp1*, a gene linked with ASD implicated in neural differentiation^13^. *Nrcam*, an ASD-associated cell-adhesion gene^14^, was downregulated in *Kdm6b*^*+/−*^ and *Kmt5b*^*+/−*^ RG1 cells (Supplementary Table 2).

Together, our data indicate a shared delay in RG lineage progression across mutants associated with ASD, but limited convergence in the potential underlying mechanisms.

## Convergent early postnatal changes in mutants linked with ASD

**Fig. 2:**
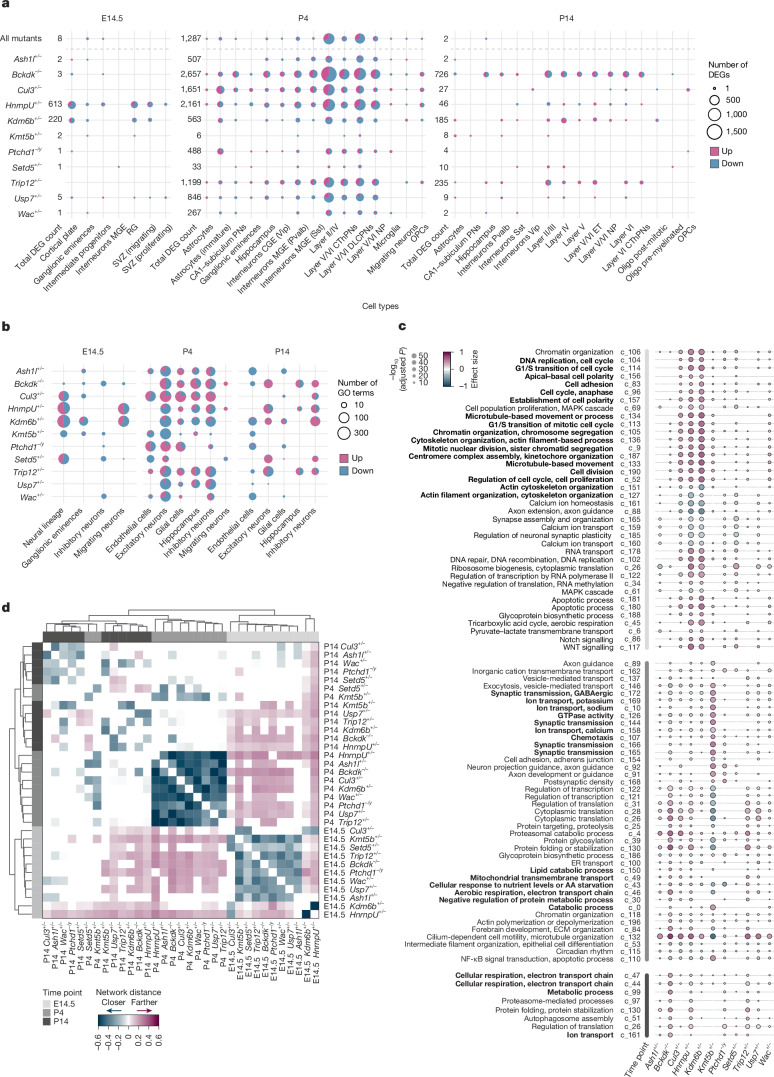
Differential gene expression highlights stage-specific convergent mechanisms. **a**, Number of DEGs (adjusted *P* < 0.05) across mutants and cell types. ‘All mutants’ denotes mutants analysed collectively compared with colony-matched controls. Total DEG counts are summarized per time point across cell types, counting repeated DEGs only once. Statistical significance was assessed using a two-sided Wald test in DESeq2 with Benjamini–Hochberg correction for multiple testing. **b**, Number of Gene Ontology (GO) terms identified by GSEA (FDR < 0.05) at each time point. The colours indicate the direction of the normalized enrichment score. Gene Ontology terms were simplified to remove redundancy; all three sub-ontologies (Biological Processes, Molecular Function and Cellular Component) are included. GSEA was performed using clusterProfiler, with a Kolmogorov–Smirnov-like statistic, permutation-based testing, and Benjamini–Hochberg correction (Methods). **c**, For each genotype, the magnitude (that is, effect size) and significance of expression changes in network clusters representing biological processes in excitatory neurons are shown. Processes mentioned in the text are highlighted in bold. Coefficients were tested against zero using two-sided Student’s *t*-tests and Benjamini–Hochberg multiple comparison test. Those with adjusted *P* < 0.1 were considered significant and marked with a black outline in the plot. AA, amino acid; ECM, extracellular matrix; ER, endoplasmic reticulum. Grey bar colours are as in panel **d**. **d**, Pairwise distances between DEG network localizations in excitatory neurons across genotypes and developmental stages. Smaller distances denote greater similarity (convergence). Animals used for analyses: *n* (E14.5) = 7 (*Cul3*^*+/−*^, *Kdm6b*^*+/−*^ and *Trip12*^*+/−*^), 6 (*Ash1l*^*+/−*^,* Bckdk*^*−/−*^, *HnrnpU*^*+/−*^, *Kmt5b*^*+/−*^, *Setd5*^*+/−*^, *Usp7*^*+/−*^, *Wac*^*+/−*^ and colony-matched controls) and 3 (*Ptchd1*^*−/y*^); *n* (P4) = 6 (*Ash1l*^*+/−*^, *Bckdk*^*−/−*^, *Cul3*^*+/−*^, *HnrnpU*^*+/−*^, *Kdm6b*^*+/−*^, *Setd5*^*+/−*^, *Trip12*^*+/−*^, *Usp7*^*+/−*^, *Wac*^*+/−*^ and colony-matched controls), 4 (*Kmt5b*^*+/−*^) and 3 (*Ptchd1*^*−/y*^); *n* (P14) = 8 (*Ash1l*^*+/−*^ and *Cul3*^*+/−*^), 7 (colony-matched controls), 6 (*Bckdk*^*−/−*^, *HnrnpU*^*+/−*^, *Kdm6b*^*+/−*^, *Kmt5b*^*+/−*^, *Setd5*^*+/−*^, *Trip12*^*+/−*^, *Usp7*^*+/−*^ and *Wac*^*+/−*^) and 3 (*Ptchd1*^*−/y*^).

**Extended Data Fig. 3:**
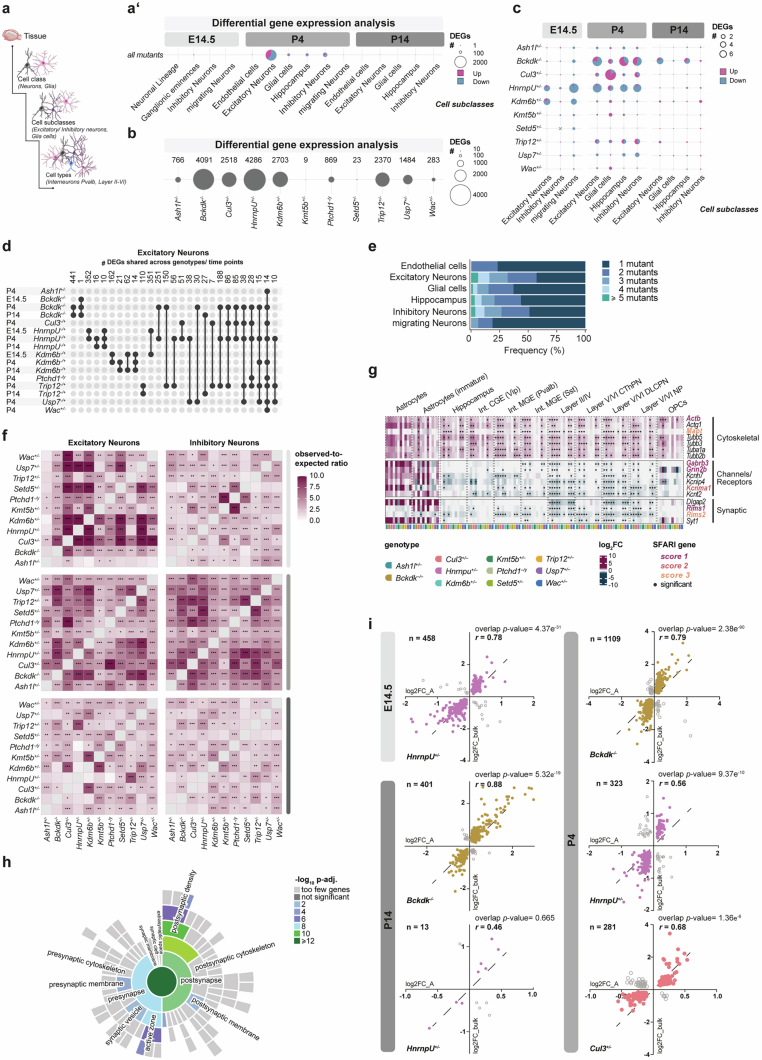
Analysis of the cellular composition and transcriptome correlations across mutants and datasets. **a**, Scheme of the hierarchy between cell classes, subclasses, and types with examples. **a’**, Number of differentially expressed genes (*p-adj*. < 0.05) in cell subclasses highlights excitatory neurons at P4 as the main cell subclass sharing DEGs across mutants. “*All mutants*” refers to the mutants analyzed collectively compared to colony-matched controls. **b**, Overview of the number of DEGs combined across cell subclasses and time points, with repeated DEGs counted only once. **c**, Down-sampling differential expression analysis to match sequencing depth across mutants, cell types, and time points. “X” marks combinations that did not meet minimal coverage requirements. **d**, UpSet R plot showing the number of DEGs identified in the excitatory neuron lineage across time points and mutants, showing that many DEGs are identified multiple times over time. Representative overlaps are shown. **e**, Proportion of DEGs identified at P4 overlapping across a given number of mutants. **f**, Overlap of differential gene expression between mutants across time in excitatory and inhibitory neurons. Each mutant pair was compared vs. distinct colony-matched control sets to ensure independent contrasts. **g**, Heatmap of fold changes of a representative subset of the most shared DEGs across cell types at P4. DEGs were organized into functional groups such as cytoskeleton, channels/receptors, and synaptic processes. ASD risk genes are highlighted with their respective SFARI gene score. Animals used for analyses: *n (E14.5)* = 7 *(Cul3*^*+/*−^*, Kdm6b*^*+/*−^*, Trip12*^*+/*−^*)*, 6 *(Ash1l*^*+/*−^*, Bckdk*^−*/*−^*, HnrnpU*^*+/*−^*, Kmt5b*^*+/*−^*, Setd5*^*+/*−^*, Usp7*^*+/*−^*, Wac*^*+/*−^, colony-matched controls), 3 (*Ptchd1*^*−/y*^); *n (P4)* = 6 (*Ash1l*^*+/*−^*, Bckdk*^−*/*−^*, Cul3*^*+/*−^*, HnrnpU*^*+/*−^*, Kdm6b*^*+/*−^*, Setd5*^*+/*−^*, Trip12*^*+/*−^*, Usp7*^*+/*−^*, Wac*^*+/*−^, colony-matched controls), 4 (*Kmt5b*^*+/*−^), 3 (*Ptchd1*^*−/y*^); *n (P14)* = 8 (*Ash1l*^*+/*−^*, Cul3*^*+/*−^), 7 (colony-matched controls), *6 (Bckdk*^−*/*−^*, HnrnpU*^*+/*−^*, Kdm6b*^*+/*−^*, Kmt5b*^*+/*−^*, Setd5*^*+/*−^*, Trip12*^*+/*−^*, Usp7*^*+/*−^*, Wac*^*+/*−^*)*, 3 (*Ptchd1*^*−/y*^). **h**, SynGO analysis of DEGs found in the highest number of ASD mutants, affecting at least one cell type, reveals enrichment for genes encoding synaptic-related proteins at P4. **i**, Correlations of log_2_FC of DEGs (*p-adj*. < 0.05) from the present snRNA-seq data (*log*_*2*_*FC_A*) with bulk RNA sequencing (*log*_*2*_*FC_bulk*) of *Bckdk*, *Cul3* and *HnrnpU* mutants vs. colony-matched controls. Spearman correlation was applied when data violated normality assumptions (*HnrnpU*^*+/*−^at E14.5, *Bckdk*^−*/*−^*, Cul3*^*+/*−^ and *HnrnpU*^*+/*−^ at P4, *Bckdk*^−*/*−^ at P14); otherwise, Pearson’s r was used (*HnrnpU*^*+/*−^ at P14). Identity line is shown for reference. Overlap *p*-value obtained from one-tailed Fisher’s exact test (enrichment). Animals used for analyses: *n* = 6 per genotype and time point. Source data

To investigate the effect of mutations linked with ASD across development and cell types, we performed pseudobulk differential gene expression analysis within each cell type, comparing all mutants collectively to WT controls, thereby identifying shared transcriptional changes (Fig. 2a, all mutants; and Supplementary Table 3). This analysis revealed numerous DEGs at P4 in layer II/IV (715 DEGs, adjusted *P* < 0.05) and layer V/VI DLCPN (600 DEGs) neurons, most of which were downregulated in mutants. Few or no DEGs were detected in other cell types and time points when considering all mutants together. Aggregating cell types into broader cell subclasses (for example, aggregating all excitatory neurons; Extended Data Fig. 3a and Supplementary Table 4), confirmed that P4 excitatory neurons showed the strongest shared DEG signal (Extended Data Fig. 3a′).

Next, we analysed each mutant separately against WT. Most genotypes showed more than 700 DEGs across cell subclasses and time points, counting repeated DEGs once (Extended Data Fig. 3b). At E14.5, changes were limited, except in *HnrnpU*^*+/−*^ and *Kdm6b*^*+/−*^, which exhibited numerous DEGs affecting cortical plate, RG and subventricular zone migrating cells (Fig. 2a, left). Conversely, P4 showed widespread transcriptional changes across genotypes and cell types (Fig. 2a, centre), with excitatory neurons most affected. Several mutants also showed many DEGs in inhibitory neurons and glia. By P14, DEG numbers declined (Fig. 2a, right). Individual mutants (aggregating replicates per genotype) consistently showed more DEGs than all genotypes together, indicating that many changes are not shared across genotypes. The distribution of upregulated and downregulated DEGs was more balanced in individual mutants than in the collective analysis. However, some mutants showed few DEGs across all analysed time points, including mice with mutations in histone modifier genes, such as *Kmt5b*, *Setd5* and *Wac* (Fig. 2a and Extended Data Fig. 3b). This suggests that mutations in this gene class may, at these stages and under baseline conditions, have subtle effects on gene expression, affecting few genes or causing small expression changes that are difficult to detect.

To control for potential effects of sequencing depth and enable comparisons of DEG counts across mutants, cell types and time points, we downsampled each group to a matched sequencing depth. This confirmed previous trends: excitatory and inhibitory neurons were similarly affected, whereas glial cells showed weaker transcriptional changes, except for *Cul3* mutants, where glial cells showed a stronger transcriptional response than neurons (Extended Data Fig. 3c).

Mutants exhibited significant DEG overlap across time points (Extended Data Fig. 3d), independently validating these DEGs and indicating persistent molecular alterations. For example, *HnrnpU*^*+/−*^ had DEGs associated with the cell cycle (for example, *Ccnd2*, *Cdk4* and *Mef2c*), axonogenesis (for example, *Dscam2*, *Sema5a* and *Robo2*) and transmembrane transporters (for example, *Cacnb3*, *Gabra4* and *Gria4*) overlapping across early and perinatal development. *Trip12*^*+/−*^ shared DEGs across time related to metabolic processes (for example, *Eef1a1* and *Nceh1*) and synaptic signalling (for example, *Nrxn3*, *Syt5* and *Rims1*) at P4 and P14 (Extended Data Fig. 3d).

Neuronal subclasses shared DEGs across mutants, particularly in P4 excitatory neurons, where approximately 10% of DEGs were shared by at least four mutants (Extended Data Fig. 3e). By contrast, the overlap between neuronal and non-neuronal cells within the same mutant was minimal. To assess whether overlaps exceeded random expectation, we compared each mutant pair against distinct sets of control samples randomly divided into two disjoint groups (Methods). The results indicated stage-dependent, non-random convergence on shared transcriptional responses across genotypes (Extended Data Fig. 3f).

Next, we compiled the most frequent DEGs across mutants and cell types (Extended Data Fig. 3g and Supplementary Table 1). This set included a large proportion of genes associated with ASD (34%) and was enriched for genes encoding synaptic proteins (*q* = 1.03 × 10^−15^; Extended Data Fig. 3h). These proteins localize to presynaptic and postsynaptic compartments and are involved in synaptic translation, actin cytoskeleton, synapse organization, and membrane channel and receptor function (Extended Data Fig. 3g,h). The directionality of these alterations was largely consistent across mutants.

Although similarities in gene expression patterns and pathway alterations were evident, genotype-restricted changes were also prominent, with at least 50% of DEGs in each cell type identified only in a single genotype (Extended Data Fig. 3e).

To validate the snRNA-seq results, we performed bulk RNA-seq on a subset of mutants and time points and, despite methodological differences, found significant DEG overlap, with concordant effect directions and magnitudes (Extended Data Fig. 3i).

These results pinpoint critical periods and highlight variations in developmental trajectories across mutants linked with ASD, with some mutants having greater effects earlier and others later. We also identified shared DEGs across mutants associated with ASD, underscoring convergent mechanisms that probably reflect stage-specific developmental processes.

## Functional pathway disruptions in mice with mutations linked with ASD

To identify biological processes affected by mutations associated with ASD, we used gene set enrichment analysis (GSEA), which captures coordinated gene set changes beyond DEG cut-offs and provides a broader perspective on cell states.

**Extended Data Fig. 4:**
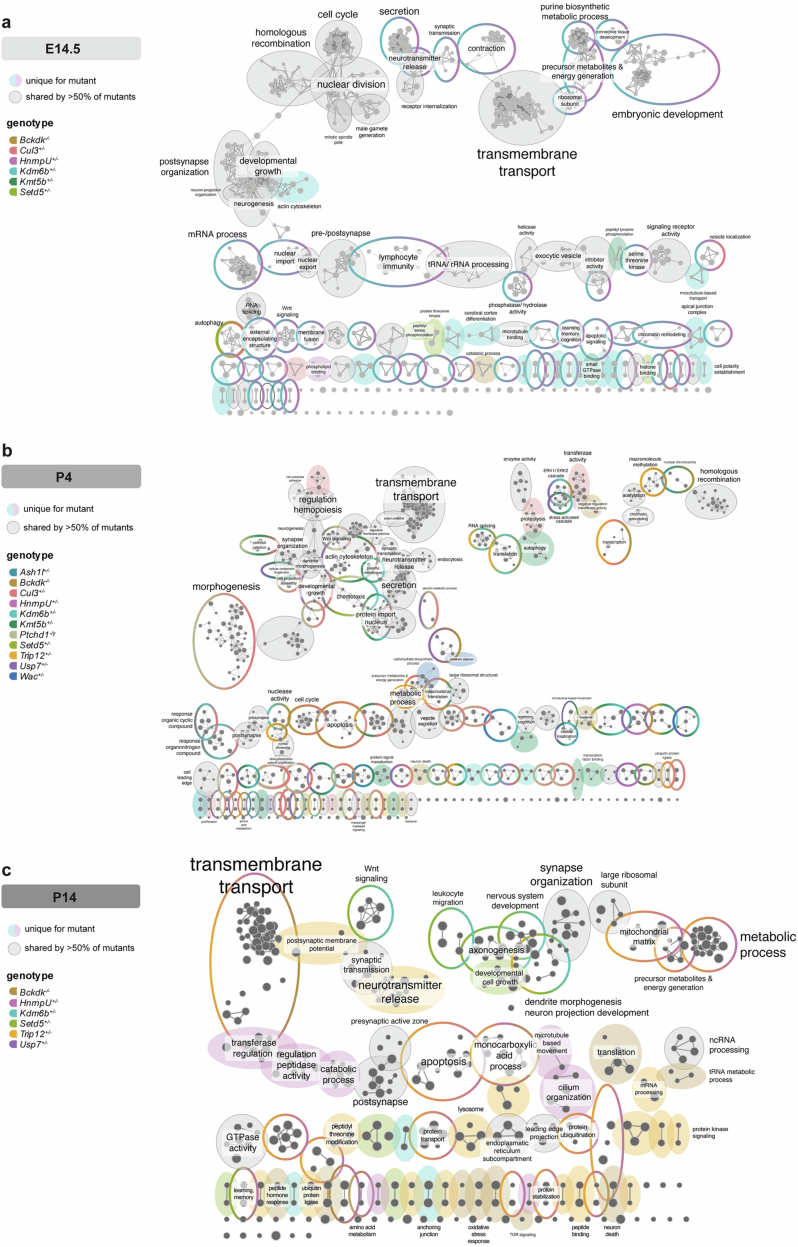
Network of the biological processes affected in ASD mutant mice. **a-c**, Networks of GO terms identified in the excitatory neuronal lineage at E14.5 (**a**), P4 (**b**), and P14 (**c**). GO terms were automatically clustered using the EnrichmentMap plugin of Cytoscape. Nodes represent functionally related gene sets. Cluster size (circles) corresponds to the total number of gene sets. Clusters in grey indicate processes shared by at least 50% of the mutants. Clusters are colored to match the color of an ASD mutant and to indicate processes unique to that mutant. Shared processes are highlighted by colored contours representing the respective ASD mutants. Animals used for analyses: *n (E14.5)* = 7 *(Cul3*^*+/*−^*, Kdm6b*^*+/*−^*, Trip12*^*+/*−^*)*, 6 *(Ash1l*^*+/*−^*, Bckdk*^−*/*−^*, HnrnpU*^*+/*−^*, Kmt5b*^*+/*−^*, Setd5*^*+/*−^*, Usp7*^*+/*−^*, Wac*^*+/*−^, colony-matched controls), 3 (*Ptchd1*^*−/y*^); *n (P4)* = 6 (*Ash1l*^*+/*−^*, Bckdk*^−*/*−^*, Cul3*^*+/*−^*, HnrnpU*^*+/*−^*, Kdm6b*^*+/*−^*, Setd5*^*+/*−^*, Trip12*^*+/*−^*, Usp7*^*+/*−^*, Wac*^*+/*−^, colony-matched controls), 4 (*Kmt5b*^*+/*−^), 3 (*Ptchd1*^*−/y*^); *n (P14)* = 8 (*Ash1l*^*+/*−^*, Cul3*^*+/*−^), 7 (colony-matched controls), *6 (Bckdk*^−*/*−^*, HnrnpU*^*+/*−^*, Kdm6b*^*+/*−^*, Kmt5b*^*+/*−^*, Setd5*^*+/*−^*, Trip12*^*+/*−^*, Usp7*^*+/*−^*, Wac*^*+/*−^*)*, 3 (*Ptchd1*^*−/y*^).

**Extended Data Fig. 5:**
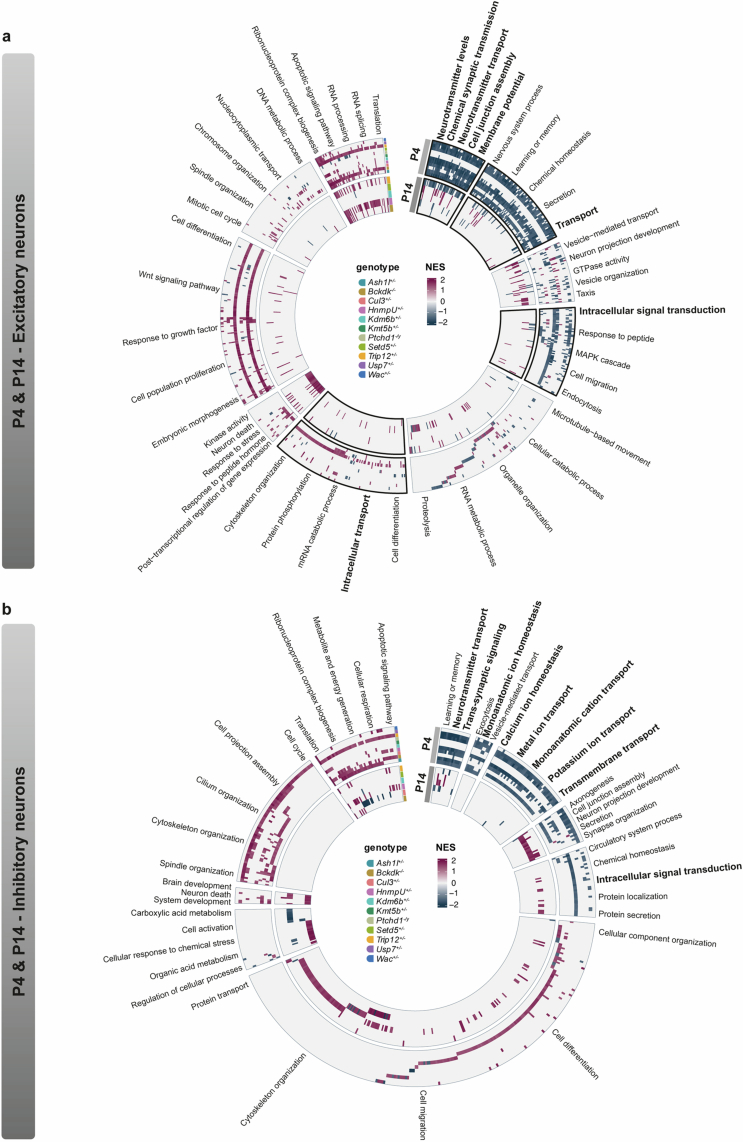
Biological processes impacted in excitatory and inhibitory neurons at postnatal stages. **a**, **b**, Circular heatmap of enriched biological processes at P4 and P14 in excitatory (**a**) and inhibitory neurons (**b**). Representative terms have been selected, and processes mentioned in the results are highlighted in bold. Animals used for analyses: *n (E14.5)* = 7 *(Cul3*^*+/*−^*, Kdm6b*^*+/*−^*, Trip12*^*+/*−^*)*, 6 *(Ash1l*^*+/*−^*, Bckdk*^−*/*−^*, HnrnpU*^*+/*−^*, Kmt5b*^*+/*−^*, Setd5*^*+/*−^*, Usp7*^*+/*−^*, Wac*^*+/*−^, colony-matched controls), 3 (*Ptchd1*^*−/y*^); *n (P4)* = 6 (*Ash1l*^*+/*−^*, Bckdk*^−*/*−^*, Cul3*^*+/*−^*, HnrnpU*^*+/*−^*, Kdm6b*^*+/*−^*, Setd5*^*+/*−^*, Trip12*^*+/*−^*, Usp7*^*+/*−^*, Wac*^*+/*−^, colony-matched controls), 4 (*Kmt5b*^*+/*−^), 3 (*Ptchd1*^*−/y*^); *n (P14)* = 8 (*Ash1l*^*+/*−^*, Cul3*^*+/*−^), 7 (colony-matched controls), *6 (Bckdk*^−*/*−^*, HnrnpU*^*+/*−^*, Kdm6b*^*+/*−^*, Kmt5b*^*+/*−^*, Setd5*^*+/*−^*, Trip12*^*+/*−^*, Usp7*^*+/*−^*, Wac*^*+/*−^*)*, 3 (*Ptchd1*^*−/y*^).

**Extended Data Fig. 6:**
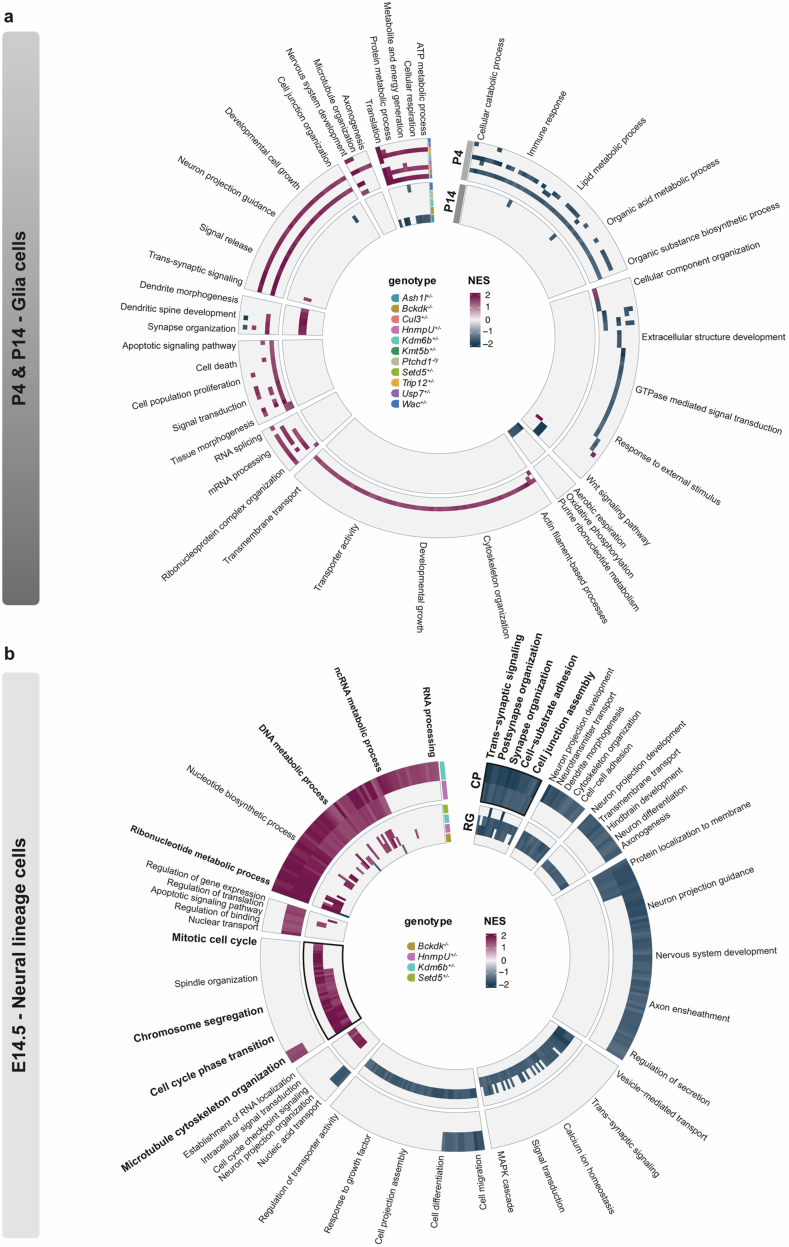
Biological processes affected in glial cells at postnatal stages, and neural lineage cells at E14.5. **a**, **b**, Circular heatmap of enriched biological processes at P4 and P14 in glial cells (**a**) and neural lineage cells, including RG and CP cells at E14.5 (**b**). Representative terms have been selected, and processes mentioned in the results are highlighted in bold. Animals used for analyses: *n (E14.5)* = 7 *(Cul3*^*+/*−^*, Kdm6b*^*+/*−^*, Trip12*^*+/*−^*)*, 6 *(Ash1l*^*+/*−^*, Bckdk*^−*/*−^*, HnrnpU*^*+/*−^*, Kmt5b*^*+/*−^*, Setd5*^*+/*−^*, Usp7*^*+/*−^*, Wac*^*+/*−^, colony-matched controls), 3 (*Ptchd1*^*−/y*^); *n (P4)* = 6 (*Ash1l*^*+/*−^*, Bckdk*^−*/*−^*, Cul3*^*+/*−^*, HnrnpU*^*+/*−^*, Kdm6b*^*+/*−^*, Setd5*^*+/*−^*, Trip12*^*+/*−^*, Usp7*^*+/*−^*, Wac*^*+/*−^, colony-matched controls), 4 (*Kmt5b*^*+/*−^), 3 (*Ptchd1*^*−/y*^); *n (P14)* = 8 (*Ash1l*^*+/*−^*, Cul3*^*+/*−^), 7 (colony-matched controls), *6 (Bckdk*^−*/*−^*, HnrnpU*^*+/*−^*, Kdm6b*^*+/*−^*, Kmt5b*^*+/*−^*, Setd5*^*+/*−^*, Trip12*^*+/*−^*, Usp7*^*+/*−^*, Wac*^*+/*−^*)*, 3 (*Ptchd1*^*−/y*^).

We performed GSEA in individual mutants at the cell-subclass level. Consistent with the DEG analysis, the effects were most pronounced at P4, although GSEA also revealed differences at E14.5 and P14 (Fig. 2b, Extended Data Figs. 4–6a and Supplementary Table 5). For example, we identified enrichment of gene sets in neural lineage cells at E14.5 in *Cul3*, *Kmt5b* and *Setd5*, in addition to *HnrnpU* and *Kdm6b* mutants (Supplementary Table 5).

At E14.5, several mutants exhibited changes in pathways associated with cell cycle regulation, chromosome segregation and neuronal projection development (Extended Data Figs. 4a and 6b). At P4, mutants displayed changes in pathways related to synaptic and ion channel functions. Synapse-related transcripts were also affected at P14 (Extended Data Fig. 4b,c).

Inhibitory neurons showed fewer enriched gene sets than excitatory neurons, but were qualitatively similarly affected, with terms related to synaptic transmission, intracellular signalling, ion channels and transmembrane transporters (Extended Data Fig. 5a,b). Glial cells were generally less affected than neurons, except in *Cul3* and *Ptchd1* mutants (Extended Data Fig. 6a and Supplementary Table 5). Certain gene sets in neurons were oppositely regulated at P4 and P14 (Extended Data Fig. 5). For instance, synaptic transmission and organization were downregulated in P4 *Kdm6b*-mutant neurons but upregulated at P14.

We refined GSEA to identify cell-type-specific features (Supplementary Table 6). For example, RG and cortical plate cells shared disruptions in neurogenesis-related terms but also showed distinct features. *HnrnpU* and *Kdm6b* mutations altered cell-cycle-relevant transcripts in RG cells, probably contributing to the developmental delay observed through pseudotime analysis. Conversely, synaptic signalling and developmental processes were predominantly affected in cortical plate cells (Extended Data Fig. 6b). GSEA also revealed more enriched terms in the excitatory neurons of layer II/IV, corticothalamic and near-projecting layer V/VI, and parvalbumin-expressing (PV^+^) interneurons, whereas hippocampal and somatostatin-expressing (SST^+^) neurons showed fewer terms (Supplementary Table 6).

Several mutants showed deregulation of axonogenesis and dendrite morphogenesis-related genes. In most mutants, DEGs associated with these processes were upregulated in PV^+^, SST^+^ and vasoactive intestinal peptide-expressing (VIP^+^) interneurons, but downregulated in excitatory layer II/IV neurons. *Cul3*^*+/−*^ and *Ptchd1*^*−*^^*/y*^ were exceptions, showing upregulation of DEGs in the latter. *Cul3* and *Ptchd1* mutants also exhibited increased synaptic gene expression in immature astrocytes and VIP^+^ interneurons (Supplementary Table 6). *Ptchd1*^*−/y*^ and *Setd5*^*+/−*^ had minimal effects on synaptic gene expression in excitatory neurons across cortical layers, aligning with previous findings^15^. Protein secretion-related processes were upregulated in hippocampal neurons and interneurons, but downregulated in cortical excitatory neurons (Supplementary Table 6).

**Extended Data Fig. 7:**
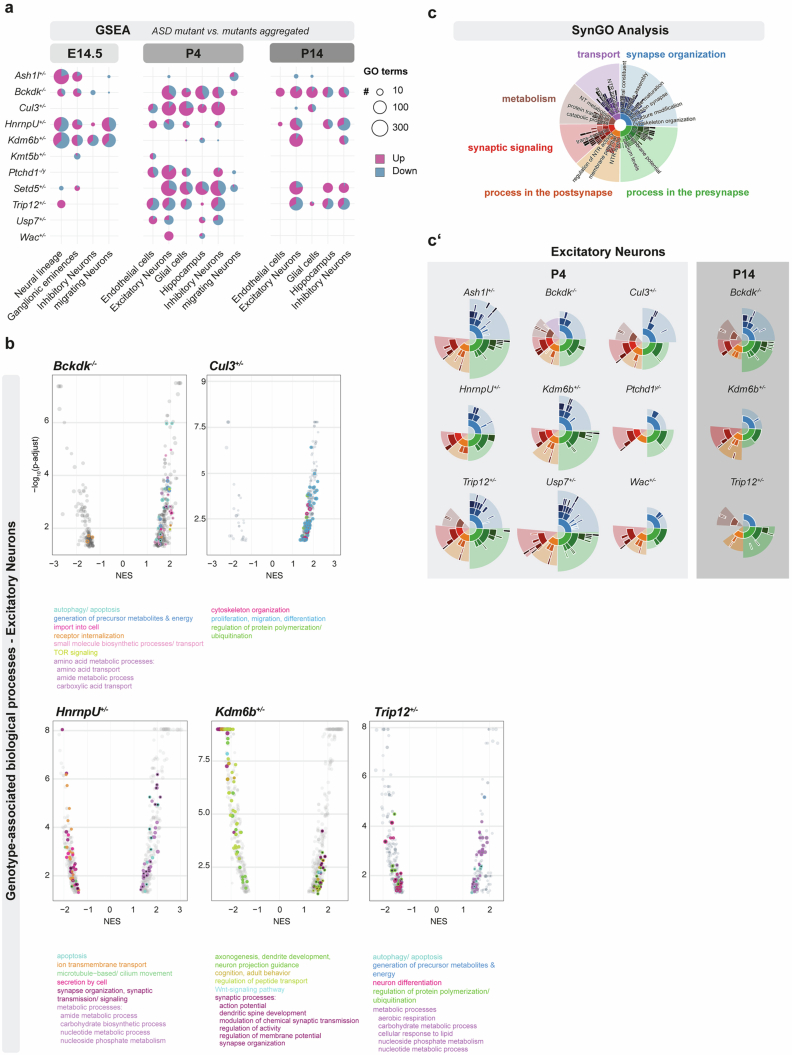
Distinct transcriptional profiles and biological processes identified in ASD mutant mice. **a**, Number of GO terms identified by the Gene Set Enrichment Analysis (GSEA, FDR < 0.05) across the analyzed time points when comparing each mutant with all the other ASD mutants collectively. Colors indicate the direction of the normalized enrichment score (NES). GO terms were simplified to remove redundancy; all three sub-ontologies (BP, MF, CC) are shown. GSEA was performed using clusterProfiler, with a Kolmogorov–Smirnov–like statistic, permutation-based testing, and Benjamini–Hochberg correction (Methods). **b**, Volcano plots of the biological processes enriched in the selected ASD mutants across the analyzed time points. Volcano plots are based on GO enrichment analysis using clusterProfiler, with a one-sided hypergeometric test and Benjamini–Hochberg correction. Colored GO terms were identified specifically in one ASD mutant. The circle size represents the gene count ratio. E14,5, colored filled circles; P4, gray filled circles; P14, black filled circles. **c**, Annotation of synaptic processes based on SynGO. **c’**, Representation of the synaptic-related DEGs identified in excitatory neurons of each ASD mutant. All the DEGs were used as input for the SynGO analysis. Only SynGO terms identified as significantly enriched in the respective mutant were retained for the generation of the heatmap. Heatmaps are colored based on the SynGO terms. Each ASD mutant shows a unique combination of affected synaptic processes. Animals used for analyses: *n (E14.5)* = 7 *(Cul3*^*+/*−^*, Kdm6b*^*+/*−^*, Trip12*^*+/*−^*)*, 6 *(Ash1l*^*+/*−^*, Bckdk*^−*/*−^*, HnrnpU*^*+/*−^*, Kmt5b*^*+/*−^*, Setd5*^*+/*−^*, Usp7*^*+/*−^*, Wac*^*+/*−^, colony-matched controls), 3 (*Ptchd1*^*−/y*^); *n (P4)* = 6 (*Ash1l*^*+/*−^*, Bckdk*^−*/*−^*, Cul3*^*+/*−^*, HnrnpU*^*+/*−^*, Kdm6b*^*+/*−^*, Setd5*^*+/*−^*, Trip12*^*+/*−^*, Usp7*^*+/*−^*, Wac*^*+/*−^, colony-matched controls), 4 (*Kmt5b*^*+/*−^), 3 (*Ptchd1*^*−/y*^); *n (P14)* = 8 (*Ash1l*^*+/*−^*, Cul3*^*+/*−^), 7 (colony-matched controls), *6 (Bckdk*^−*/*−^*, HnrnpU*^*+/*−^*, Kdm6b*^*+/*−^*, Kmt5b*^*+/*−^*, Setd5*^*+/*−^*, Trip12*^*+/*−^*, Usp7*^*+/*−^*, Wac*^*+/*−^*)*, 3 (*Ptchd1*^*−/y*^).

To identify genotype-associated processes, we compared each mutant against the aggregate of the other ten mutants (Extended Data Fig. 7a and Supplementary Table 7). The number of affected processes yielded a pattern similar to the mutant–WT comparison, with most changes occurring at P4. We then confirmed that processes identified in the cross-mutant comparison were also affected in the mutant–WT comparison. For example, *Cul3* mutants showed enrichment for genes related to cell migration (Extended Data Fig. 7b), aligning with previous results^16^. *Bckdk*^*−/−*^ and *Trip12*^*+/−*^ showed upregulation of metabolism and energy production genes. Consistent with *Bckdk* function^17^, amino acid-related genes (for example, *Bcat1*, *Cars* and *Slc7a5*; Supplementary Tables 3 and 4) were altered across multiple cell types and time points in *Bckdk*^*−/−*^ but not other mutants. Our data suggest that these changes predominantly affect neurons rather than glial cells (Extended Data Fig. 3c). *Trip12* mutation affected processes related to cellular respiration and energy production, including glycolysis (for example, *Pdk3* and *Mpc2*), the tricarboxylic acid cycle (for example, *Mdh1* and *Mdh2*) and lipid metabolism (for example, *Lactb*; Extended Data Fig. 7b and Supplementary Table 7). *HnrnpU* mutants showed changes in transcripts related to secretion and cilium development (Extended Data Fig. 7b and Supplementary Table 7), consistent with previous literature^18^. Finally, *Kdm6b* mutants exhibited differential expression of WNT signalling-related genes, often associated with ASD^19,20^ (Extended Data Fig. 7b and Supplementary Table 7).

In summary, these results show convergent and genotype-associated changes across ASD models. Although most mutants exhibited dysregulation of synaptic transmission and ion channel pathways, some alterations appeared enriched within particular genotypes, including alterations in cytoskeleton dynamics, cilium function and metabolism.

## Network analysis reveals convergent phenotypes

As GSEA revealed commonalities across mutants, we evaluated the extent of overlaps across genotypes and developmental stages using network colocalization analysis on a protein–protein interaction network. Functionally related proteins were grouped into clusters via averaged random walks, and each cluster assessed for functional enrichment (Methods).

**Extended Data Fig. 8:**
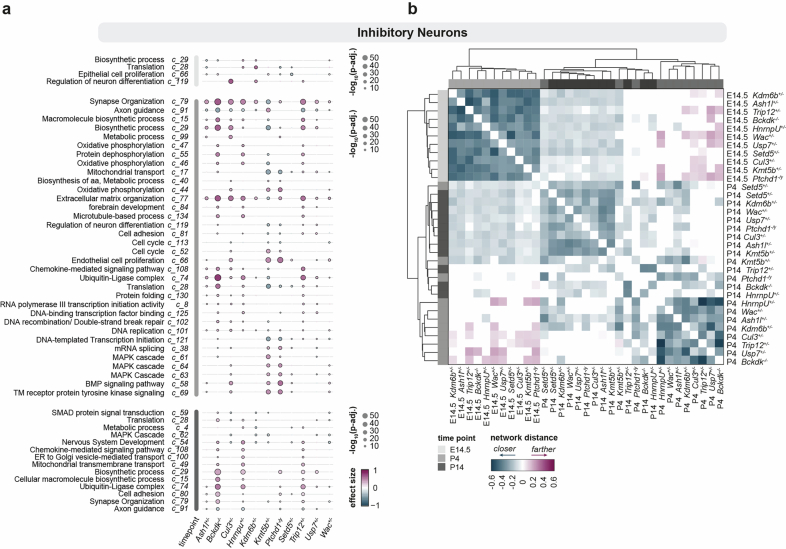
Network colocalization in inhibitory neurons. **a**, For each genotype, effect sizes and significance of expression changes mapped onto protein–protein interaction (PPI) network clusters (biological processes) in inhibitory neurons. Coefficients were tested against zero using two-sided t-tests and Benjamini–Hochberg multiple comparison test. Those with *p-adj*. <0.1 were considered significant and marked with a black outline in the plot. **b**, Pairwise distances between DEG network localizations of inhibitory neurons across genotypes and developmental stages. Smaller distances denote greater similarity (convergence). Animals used for analyses: *n (E14.5)* = 7 *(Cul3*^*+/*−^*, Kdm6b*^*+/*−^*, Trip12*^*+/*−^*)*, 6 *(Ash1l*^*+/*−^*, Bckdk*^−*/*−^*, HnrnpU*^*+/*−^*, Kmt5b*^*+/*−^*, Setd5*^*+/*−^*, Usp7*^*+/*−^*, Wac*^*+/*−^, colony-matched controls), 3 (*Ptchd1*^*−/y*^); *n (P4)* = 6 (*Ash1l*^*+/*−^*, Bckdk*^−*/*−^*, Cul3*^*+/*−^*, HnrnpU*^*+/*−^*, Kdm6b*^*+/*−^*, Setd5*^*+/*−^*, Trip12*^*+/*−^*, Usp7*^*+/*−^*, Wac*^*+/*−^, colony-matched controls), 4 (*Kmt5b*^*+/*−^), 3 (*Ptchd1*^*−/y*^); *n (P14)* = 8 (*Ash1l*^*+/*−^*, Cul3*^*+/*−^), 7 (colony-matched controls), *6 (Bckdk*^−*/*−^*, HnrnpU*^*+/*−^*, Kdm6b*^*+/*−^*, Kmt5b*^*+/*−^*, Setd5*^*+/*−^*, Trip12*^*+/*−^*, Usp7*^*+/*−^*, Wac*^*+/*−^*)*, 3 (*Ptchd1*^*−/y*^).

For each genotype, we assessed the magnitude (effect size) and significance of expression changes across clusters, mapping the network-wide effect of each mutation on biological processes, with emphasis on stage-restricted effects (Fig. 2c and Extended Data Fig. 8a).

Consistent with the GSEA, at E14.5 (Fig. 2c, top), we observed enrichment of actin cytoskeleton, cell cycle and proliferation pathways in excitatory neurons, particularly in *HnrnpU* and *Kdm6b* mutants, but also in *Cul3* mutants, consistent with earlier reports^16^. Network-level analysis also highlighted changes in WNT signalling, particularly in *HnrnpU* and *Kdm6b*. At postnatal stages (Fig. 2c, centre and bottom), the network highlighted effects on synaptic processes, ion transport and metabolism, reflecting the transition towards neuronal specialization. Compared with the other mutants, *Kmt5b* mutants showed an inverse effect size pattern at P4, suggesting counteractive or compensatory influences within shared biological systems, potentially reflecting an altered regulatory role. At P14, metabolism-related processes emerged, with *Bckdk*, *HnrnpU* and *Trip12* mutants exhibiting larger effect sizes (Extended Data Fig. 7b).

Next, we compared network localization profiles of top-ranked DEGs across genotypes and developmental time points to evaluate the similarity of affected gene systems. Network localization and corresponding similarity scores were primarily shaped by developmental stage (Fig. 2d and Extended Data Fig. 8b). Within each developmental stage, mutants were more similar than expected by chance, indicating that their DEGs cluster within comparable network regions. By P14, however, mutants were less similar than at earlier stages, particularly in excitatory neurons. Thus, mutations associated with ASD influence common, stage-specific processes but progressively diverge into distinct genotype-associated transcriptional programs.

## Synaptic and ion channel changes in ASD models

Given the link between ASD and excitation–inhibition imbalances^21–24^, we explored ion channel alterations, focusing on calcium, potassium and sodium channels (Supplementary Tables 3 and 4). *Scn1a* was among the most commonly affected genes. *Scn1a* encodes a sodium channel involved in the generation of action potentials and mutated in Dravet syndrome^25^. Mutants showing *Scn1a* downregulation often also exhibited reduced *Scn8a* and increased *Scn9a* expression. *Scn9a* physically and genetically interacts with *Scn1a*^26,27^, potentially reflecting compensatory mechanisms. Similarly, several potassium and calcium channels were affected in excitatory neurons, including *Kcnv1*, which encodes a modifier subunit of voltage-gated potassium channels, and *Kcnq3*, which encodes a mediator of neuronal M-currents and is mutated in familial epilepsy syndromes^28–30^.

We explored synaptic functions using SynGO (Extended Data Fig. 7c). Most mutants disrupted both postsynaptic and presynaptic processes, including synapse organization and cytoskeleton, vesicle release, signalling and neurotransmitter receptor organization (Extended Data Fig. 7c′ and Supplementary Table 8). None of the mutants showed enrichment for transcripts related to axon or dendritic transport. Metabolic and synaptic translation processes were affected in *Bckdk*, *Cul3* and *Trip12* mutants, consistent with their functions related to proteostasis^16,31–34^. Both *Ash1l* and *Cul3* mutants showed enrichment for synaptic actin cytoskeleton genes, with *Ash1l* mutants affecting actin cytoskeleton dynamics, and *Cul3* mutants affecting actin assembly. In addition, *Ash1l* mutants showed enrichment for synaptic vesicle priming and release, terms absent in the SynGO analysis of *Cul3* mutants.

Commonly altered transcripts related to neurotransmitter receptors and synaptic transmission included upregulated *Cacng4*, an AMPA receptor modulator, and downregulated *Camk2a*, a calcium/calmodulin-dependent kinase involved in neuronal and glutamatergic synaptic functions. *Rims1* and *Rims2*, encoding key organizers of the presynaptic compartment, were downregulated across multiple genotypes and time points. *Stxbp5l*, encoding a syntaxin-binding protein relevant for vesicular trafficking and neurotransmitter release^35^, was also downregulated in most mutants.

Overall, ion channel and synaptic gene alterations represent a convergent molecular theme across mutants associated with ASD. Yet, each genotype exhibits a distinct combination of affected pathways, underscoring shared biology layered on pronounced heterogeneity.

## Electrophysiology correlates with transcriptomics

**Fig. 3:**
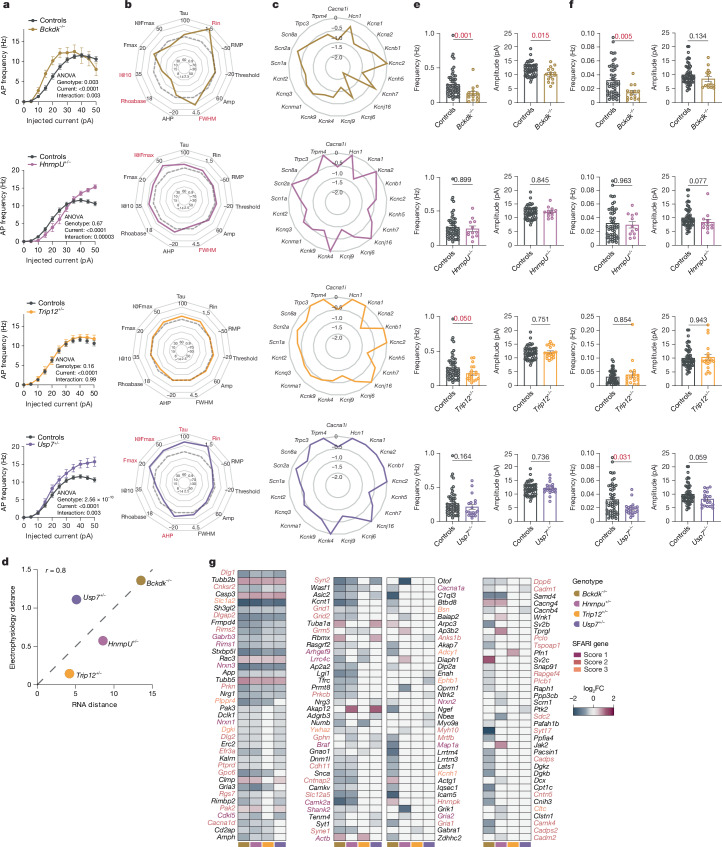
Correlation of electrophysiological and transcriptomic changes in mutants associated with ASD. **a**, Frequency–current curves for *Bckdk*^*−/−*^, *HnrnpU*^*+/−*^, *Trip12*^*+/−*^ and *Usp7*^*+/−*^ mutants (top to bottom) versus pooled controls (colony matched and littermate; two-way analysis of variance (ANOVA) and Sidak’s multiple comparison test). AP, action potential. **b**, Radar plots summarizing membrane and action potential properties; significant features are highlighted in red; values are normalized to matched controls. Colours are as in panel **a**. The dashed grey line indicates normalized WT properties. AHP, action potential afterhyperpolarization; Amp, amplitude; *F*_max_, maximal firing rate; FWHM, full-width at half-maximum; I, current; Rin, input resistance; RMP, resting membrane potential; Tau, membrane time constant. **c**, Radar plots of DEGs encoding ion channels involved in action potential generation (log_2_FC; non-significant channels set to 0). Colours are as in panel **a**. **d**, Spearman correlation between Euclidean distances of electrophysiological properties (**b**) and channel expression profiles (**c**); weighted by −log_10_(adjusted *P*). The identity line for reference is shown. **e**,**f**, mEPSCs (**e**) and mIPSCs (**f**) in mutants and controls. Statistically significant values are shown in red. Animals and cells used for electrophysiology analysis: *n* (animals; *Bckdk*^*−/−*^) = 5, *n* (*HnrnpU*^*+/−*^) = 4, *n* (*Usp7*^*+/−*^) = 7 and *n* (*Trip12*^*+/−*^) = 7; *n* (pooled controls: colony-matched controls *n* = 11, littermate controls *n* = 7) total = 18; *n* (cells; detailed cell counts per experiment are provided in the Source Data); unpaired two-tailed *t*-test for normally distributed data, and unpaired two-tailed Mann–Whitney test for non-normally distributed data. **g**, Heatmap of synaptic DEGs in layer II/IV neurons at P4, showing convergence across mutants. DEGs are ordered by the degree of shared differential expression; risk genes for ASD are highlighted and coloured by SFARI score. Data are mean ± s.e.m. (**a**,**e**,**f**). Source data

**Extended Data Fig. 9:**
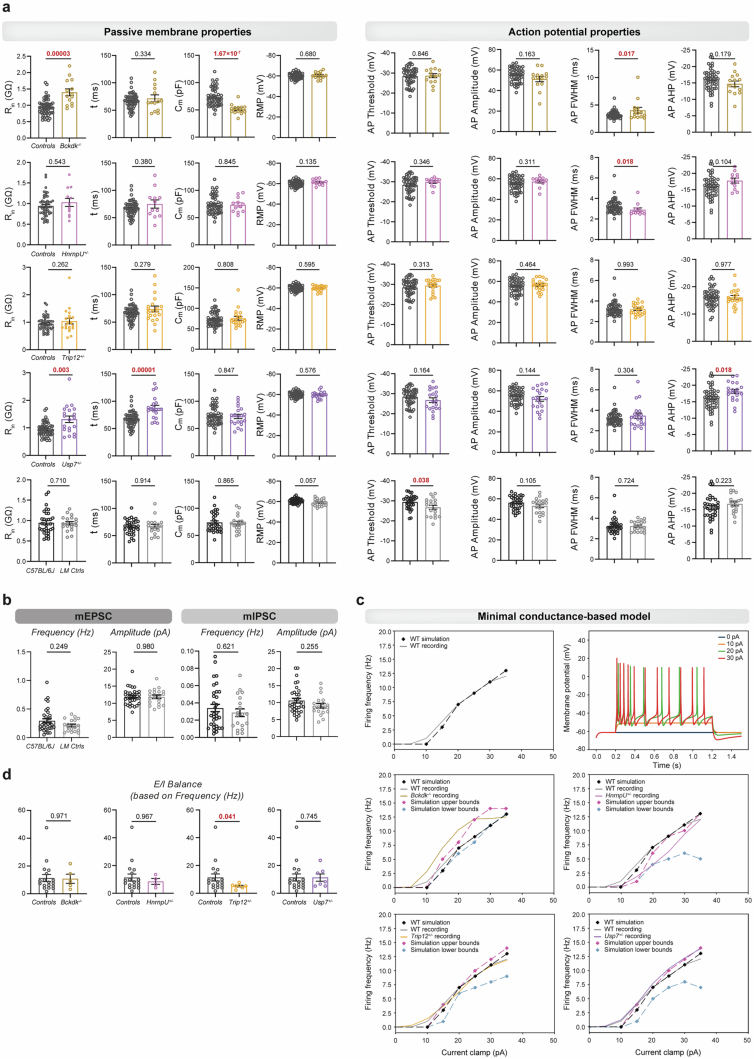
Electrophysiological recordings and minimal conductance model. **a**, Recorded membrane and action potential properties of *Bckdk*^−*/*−^*, HnrnpU*^*+/*−^*, Trip12*^*+/*−^, and *Usp7*^*+/*−^ mutant animals alongside pooled colony-matched and littermate controls (top to bottom). **b**, Both colony-matched and littermate controls were tested. Given there were no overt differences between the control groups, data were pooled for analyses. **c**, Minimal conductance-based model of a P7 Layer II/III pyramidal neuron, parametrically adjusted according to the direction and magnitude of observed ion channel expression changes to reproduce the diversity of AP and F–I curves. By tuning channel activities within transcriptomically informed ranges, the model approximated the experimentally observed electrophysiological changes and recapitulated recorded responses. The F–I curve shows mean wild-type recordings (solid line) and model output (dashed line; top left). Example voltage traces illustrate firing responses of the simulated neuron to increasing current injections during a 1-second voltage response (top right). **d**, Excitation/Inhibition (E/I) ratio derived from AP frequency. Data are mean ± SEM. *n (animals; Bckdk*^−*/*−^*)=* 5, *n (HnrnpU*^*+/*−^*)=* 4, *n (Usp7*^*+/*−^*)=* 7, *n (Trip12*^*+/*−^*)=* 7, *n* (pooled controls)*= 18, n* (colony-matched controls)*=* 11, *n* (littermate controls)*=* 7; *n(cells*; detailed cell counts per experiment are provided in the Source Data); unpaired two-tailed t-test for normally distributed data, unpaired two-tailed Mann-Whitney test for non-normally distributed data in **a, b** and **d**. Source data

To test whether transcriptional changes in ion channel and synaptic genes translate into functional abnormalities, we selected a subset of mutants (*Bckdk*, *HnrnpU*, *Trip12* and *Usp7*) and performed whole-cell patch-clamp recordings from layer II/III somatosensory cortical pyramidal neurons at P7, the earliest stage near P4 with robust synaptic activity (Fig. 3). We quantified passive membrane and action potential properties (frequency–current curves; Fig. 3a,b), then blocked action potentials to isolate miniature excitatory and inhibitory postsynaptic currents (mEPSCs and mIPSCs, respectively) in mutants and controls (Fig. 3e,f and Extended Data Fig. 9a).

Three of the four models showed altered intrinsic excitability and/or input–output (frequency–current) relationships (Fig. 3a,b). The exception was the *Trip12* mutant, which, consistent with its subtle transcriptional changes in layer II/IV excitatory neurons at P4 (Fig. 3c), exhibited no obvious differences in passive or active membrane properties (Fig. 3a,b).

To link molecular and cellular phenotypes, we computed Euclidean distances between each mutant and controls based on the expression of ion channels linked to the measured properties (Fig. 3c) and compared them with distances in a multivariate space of intrinsic and action potential features (Fig. 3a,b). These distances correlated (Fig. 3d), indicating that greater transcriptional divergence corresponds to larger physiological deviations.

Thus, although each genotype presented a distinct combination of molecular and functional alterations, the overall coupling between transcriptional and physiological changes was robust. To assess this relationship, we generated a minimal conductance-based model of a P7 layer II/III pyramidal neuron recapitulating the passive properties and the frequency–current relationship of a WT neuron. We then parametrically adjusted the ion channel conductances of the model according to the direction and magnitude of expression changes inferred from the transcriptomic data. This framework enabled testing whether small shifts in channel composition could reproduce the observed diversity of action potential and frequency–current curves. Indeed, tuning channel activities within transcriptomically informed ranges allowed us to reproduce the experimentally observed electrophysiological changes and recapitulate the recorded responses (Extended Data Fig. 9c).

Miniature synaptic recordings similarly reflected transcriptional signatures of synaptic dysfunction. mEPSC and mIPSC frequencies were reduced in three mutants (*Bckdk*, *Usp7* and *Trip12*; Fig. 3e,f), matching downregulated presynaptic release machinery genes^36–40^ (for example, *Rims1/2*, *Syn2* and *Dnm1l*; Fig. 3g and Supplementary Table 8). Changes in mIPSC frequency (Fig. 3f) were comparable with those in mEPSCs. *Trip12* mutants were the outlier with a small but significant excitation–inhibition frequency imbalance (Extended Data Fig. 9d). *Bckdk* mutants also showed reduced mEPSC amplitude (Fig. 3e), accompanied by selective downregulation of *Gria1* (Fig. 3g), a determinant of quantal amplitude^41^. Our data support a dose-like coupling: the greater the transcriptional deviation of synaptic-related and ion channel-related genes, the larger the intrinsic and synaptic physiological effects.

An exception were *HnrnpU* mutants, which, despite broad transcriptomic alterations, showed mild miniature synaptic phenotypes. DEG (adjusted *P* < 0.01) annotation with SynGO revealed downregulation of a core set of synaptic changes but also displayed genotype-associated upregulated DEGs (Fig. 3g and Extended Data Fig. 7c′), suggesting combinatorial effects. In *HnrnpU* mutants, several synaptic and signalling genes were upregulated and plausibly buffer presynaptic deficits (Fig. 3g), thereby helping to maintain near-control mEPSC or mIPSC rates. For example, *Ap3b2* promotes synaptic vesicle biogenesis and recycling^42^, and its upregulation could sustain vesicle supply and oppose frequency drops. *Tprgl1* modulates presynaptic release probability^43^, and *Diaph1* can stabilize dendritic spines and presynaptic endocytic scaffolds^44^, preserving transmission efficacy. *Akap12*, a regulator of synaptic plasticity^45^, was strongly upregulated in excitatory neurons in *HnrnpU* and *Usp7* mutants (Fig. 3g), both of which lacked changes in mEPSC frequency. By contrast, *Akap12* was not differentially regulated in *Bckdk* and *Trip12* mutants, which showed reduced mEPSC frequency, consistent with a potential compensatory role for *Akap12* in maintaining excitatory transmission. These changes may offset presynaptic deficits in *HnrnpU*, explaining the absence of pronounced mEPSC or mIPSC frequency or amplitude changes. More broadly, although electrophysiological and transcriptomic changes correlate at the pathway level, the net phenotype probably reflects multiple small, opposing effects and possible post-transcriptional buffering, making one-to-one attributions difficult.

## Chromatin accessibility reveals regulatory dynamics

Although snRNA-seq revealed transcriptional variations across developmental stages and mutants, it offers limited insight into upstream regulatory elements. Our single-nucleus multi-omics approach links chromatin accessibility (snATAC-seq) to matched transcriptomes (snRNA-seq) within the same nuclei, enabling investigation of the gene-regulatory landscape underlying the observed expression changes.

**Fig. 4:**
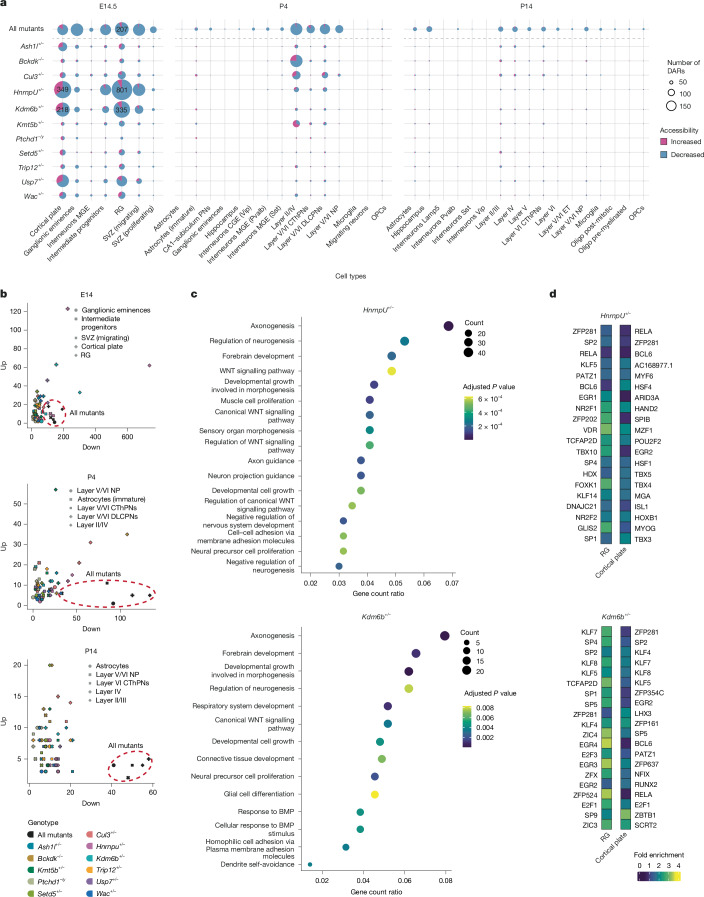
Chromatin accessibility changes in mutants associated with ASD. **a**, Number of DARs (*P* < 0.001) across cell types and mutants. All mutants indicates pooled mutants compared with colony-matched controls. Five comparisons exceeded the plot scale; thus, exact values are shown. **b**, Comparison of DARs in individual mutants versus WT reveals both decreased and increased accessibility, whereas the aggregate of all mutants shows decreased accessibility. **c**, Enriched biological processes of differentially accessible genes in RG cells of *HnrnpU* and *Kdm6b* mutants. Gene Ontology enrichment analysis was performed using clusterProfiler with a one-sided hypergeometric test and Benjamini–Hochberg correction. **d**, Heatmaps of transcription factor motif enrichment analysis in RG DARs from *HnrnpU* and *Kdm6b* mutants (adjusted *P* < 0.05), including regulators associated with neurogenesis and progenitor differentiation. Transcription factors are sorted by ascending adjusted *P* values. Animals used for analyses: *n* (E14.5) = 7 (*Cul3*^*+/−*^, *Kdm6b*^*+/−*^ and *Trip12*^*+/−*^), 6 (*Ash1l*^*+/−*^, *Bckdk*^*−/−*^, *HnrnpU*^*+/−*^, *Kmt5b*^*+/−*^, *Setd5*^*+/−*^, *Usp7*^*+/−*^, *Wac*^*+/−*^ and colony-matched controls) and 3 (*Ptchd1*^*−/y*^); *n* (P4) = 6 (*Ash1l*^*+/−*^,* Bckdk*^*−/−*^, *Cul3*^*+/−*^, *HnrnpU*^*+/−*^, *Kdm6b*^*+/−*^, *Setd5*^*+/−*^, *Trip12*^*+/−*^, *Usp7*^*+/−*^, *Wac*^*+/−*^ and colony-matched controls), 4 (*Kmt5b*^*+/−*^) and 3 (*Ptchd1*^*−/y*^); *n* (P14) = 8 (*Ash1l*^*+/−*^ and *Cul3*^*+/−*^), 7 (colony-matched controls), 6 (*Bckdk*^*−/−*^, *HnrnpU*^*+/−*^, *Kdm6b*^*+/−*^, *Kmt5b*^*+/−*^, *Setd5*^*+/−*^, *Trip12*^*+/−*^, *Usp7*^*+/−*^ and *Wac*^*+/−*^) and 3 (*Ptchd1*^*−/y*^). Statistical significance was assessed using two-tailed Wald test without multiple comparisons testing as implemented in DESeq2 (**a**,**d**).

As with snRNA-seq, we analysed snATAC-seq across all mutants and by genotype. Accessibility patterns varied from transcriptional profiles. We detected the highest number of differentially accessible regions (DARs) at E14.5. At this stage, all cell types showed DARs, with RG cells showing the greatest number (Fig. 4a and Supplementary Table 9). DARs declined at later stages, except in P4 excitatory neurons, which retained comparable high counts. Overall, the collective mutant-versus-WT comparison (Fig. 4a, all mutants) highlighted decreased chromatin accessibility (Fig. 4b), mirroring shared transcriptional downregulation (Fig. 2a).

**Extended Data Fig. 10:**
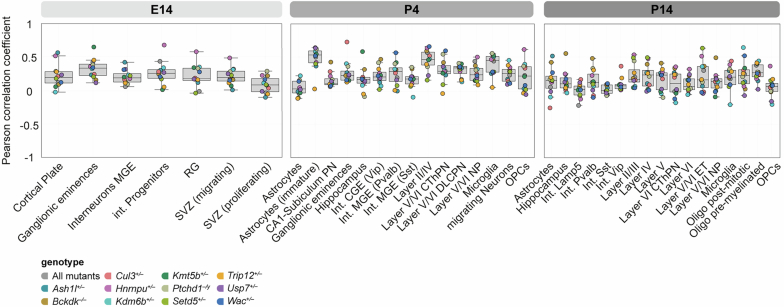
Correlation of snRNA- and snATAC-seq. Pearson correlation between fold changes of genes (*p*-value < 0.1) of pseudobulk chromatin accessibility and gene expression across cell types and time points. Center lines represent medians, the box limits show the 25-75% interquartile range (IQR), whiskers extend to the furthest data points within 1.5 × IQR from the box edges, and data points outside this range are represented as dots. Statistical significance was assessed using two-tailed Wald test without multiple comparisons testing. Animals used for analyses: *n (E14.5)* = 7 *(Cul3*^*+/*−^*, Kdm6b*^*+/*−^*, Trip12*^*+/*−^*)*, 6 *(Ash1l*^*+/*−^*, Bckdk*^−*/*−^*, HnrnpU*^*+/*−^*, Kmt5b*^*+/*−^*, Setd5*^*+/*−^*, Usp7*^*+/*−^*, Wac*^*+/*−^, colony-matched controls), 3 (*Ptchd1*^*−/y*^); *n (P4)* = 6 (*Ash1l*^*+/*−^*, Bckdk*^−*/*−^*, Cul3*^*+/*−^*, HnrnpU*^*+/*−^*, Kdm6b*^*+/*−^*, Setd5*^*+/*−^*, Trip12*^*+/*−^*, Usp7*^*+/*−^*, Wac*^*+/*−^, colony-matched controls), 4 (*Kmt5b*^*+/*−^), 3 (*Ptchd1*^*−/y*^); *n (P14)* = 8 (*Ash1l*^*+/*−^*, Cul3*^*+/*−^), 7 (colony-matched controls), *6 (Bckdk*^−*/*−^*, HnrnpU*^*+/*−^*, Kdm6b*^*+/*−^*, Kmt5b*^*+/*−^*, Setd5*^*+/*−^*, Trip12*^*+/*−^*, Usp7*^*+/*−^*, Wac*^*+/*−^*)*, 3 (*Ptchd1*^*−/y*^).

Next, we correlated log_2_ fold change (log_2_FC) values from snRNA-seq with the snATAC-seq data by aggregating peaks across gene bodies. We found a strong correlation in immature astrocytes and layer II/IV neurons at P4 (Extended Data Fig. 10), probably reflecting the high number of shared transcriptomic changes in these cells.

Next, we analysed snATAC-seq data stratified by genotype. Fewer DARs were detected in the single-mutant analyses than in the collective analysis (Fig. 4a,b), with *HnrnpU* and *Kdm6b* mutants showing the highest numbers of DARs. At E14.5, *HnrnpU*^*+/−*^ and *Kdm6b*^*+/−*^ RG cells showed 801 and 335 DARs, respectively. Although *HnrnpU* and *Kdm6b* mutants had fewer DEGs in RG cells at E14.5, RG cells exhibited the greatest differences in chromatin accessibility in these mutants. Although other mutants had fewer DARs overall, most showed more DARs in cortical plate and RG cells at E14.5 than in any other cell type at later stages. The *Bckdk*^*−/−*^ mutant was an exception, with the largest number of DARs in layer II/IV at P4, matching the mutant-specific and cell-type-specific transcriptomic profile. The general decrease in DARs over time aligns with reports showing that chromatin gets locked in specific states with neural differentiation^46,47^.

Comparison of snATAC-seq and snRNA-seq revealed strong log_2_FC correlations in *HnrnpU*^*+/−*^ RG and cortical plate cells (Extended Data Fig. 10). Gene Ontology enrichment of differentially accessible genes in RG cells overlapped terms identified at the transcript level (Fig. 4c), including progenitor proliferation and forebrain development. WNT signalling and BMP activity emerged as altered processes in both *HnrnpU*^*+/−*^ and *Kdm6b*^*+/−*^ RG cells. Although we observed changes in WNT-related transcripts in *Kdm6b* mutants, they became apparent at the chromatin level in *HnrnpU*^*+/−*^ progenitors as well. *Kdm6b*^*+/−*^ RG cells also showed responses to BMP activity, which promotes neural progenitor division during neurogenesis^48,49^.

To identify gene-regulatory elements, we performed motif enrichment analysis of regions with decreased accessibility (Fig. 4d). DARs in *HnrnpU*^*+/−*^ RG cells were enriched for multiple transcription factors (adjusted *P* < 0.01) linked to neurodevelopment and RG lineage progression^50–53^. Among the top hits was Sp2, which regulates cell cycle length in neural progenitor cells^54^. Several of these transcription factors were similarly enriched in *Kdm6b* mutants, suggesting shared regulatory mechanisms.

## Sex-specific gene expression alterations

**Extended Data Fig. 11:**
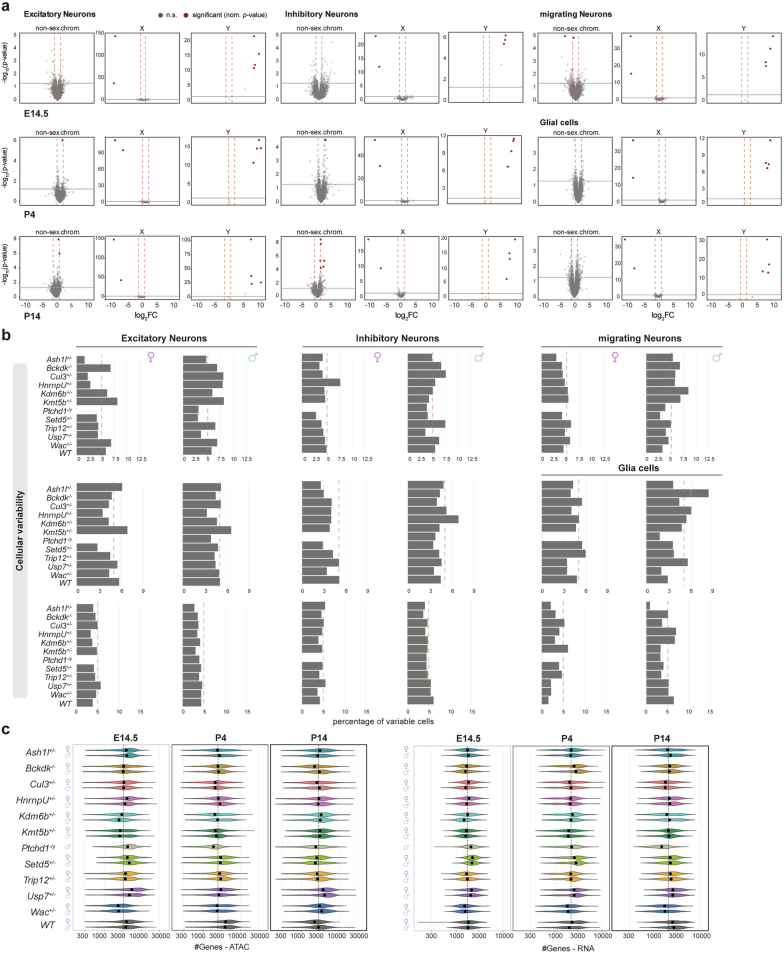
Differential gene expression analysis and quality control of female and male mice. **a**, Differential gene expression analysis of control female and male mice exhibits very little transcriptional differences. DEGs with a nominal *p*-value < 0.05 are shown. Statistical significance was assessed using two-tailed Wald test without multiple comparisons testing. **b**, Gene expression differences observed through pairwise comparison of random cells between females and males of the same genotype suggest minimal variability in gene expression across cells. Animals used for analyses: *n (females and males; E14.5)* = 3; *n (females and males; P4)* = 3*; n (females; P14)* = 4; *n (males; P14)* = 3**. c**, Quality metrics for number of genes sequenced in snRNA- and snATAC-seq in females and males across time points. The dotted lines represent the median values. Animals used for analyses: *n (females; E14.5)* = 4 *(Cul3*^*+/*−^*, Kdm6b*^*+/*−^*, Trip12*^*+/*−^*)*, 3 (*Ash1l*^*+/*−^, *Bckdk*^−*/*−^*, HnrnpU*^*+/*−^*, Kmt5b*^*+/*−^*, Setd5*^*+/*−^*, Usp7*^*+/*−^*, Wac*^*+/*−^, colony-matched controls); *n (males; E14.5)* = 3 per genotype; *n (females and males; P4)* = 3 per genotype*; n (females; P14)* = 4 (*Ash1l*^*+/*−^, *Cul3*^*+/*−^, Controls), 3 (*Bckdk*^−*/*−^*, HnrnpU*^*+/*−^*, Kdm6b*^*+/*−^*, Kmt5b*^*+/*−^*, Trip12*^*+/*−^*, Usp7*^*+/*−^*, Wac*^*+/*−^), 2 (*Setd5*^*+/*−^); *n (males; P14)* = 4 (*Ash1l*^*+/*−^, *Cul3*^*+/*−^, *Setd5*^*+/*−^); 3 (*Bckdk*^−*/*−^*, HnrnpU*^*+/*−^, *Kdm6b*^*+/*−^*, Kmt5b*^*+/*−^, *Ptchd1*^*−/y*^, *Trip12*^*+/*−^*, Usp7*^*+/*−^*, Wac*^*+/*−^, colony-matched controls).

An open question in ASD research is its different presentation in female and male individuals. As the selected genes associated with ASD are found mutated in both sexes, we tested whether their mutations manifest similarly between sexes. Aside from sex-chromosome genes, we observed few DEGs between female and male controls (Extended Data Fig. 11a and Supplementary Table 10). We also found no sex differences in gene expression variability, transcript numbers or cell-type clustering (Extended Data Fig. 11b,c).

**Fig. 5:**
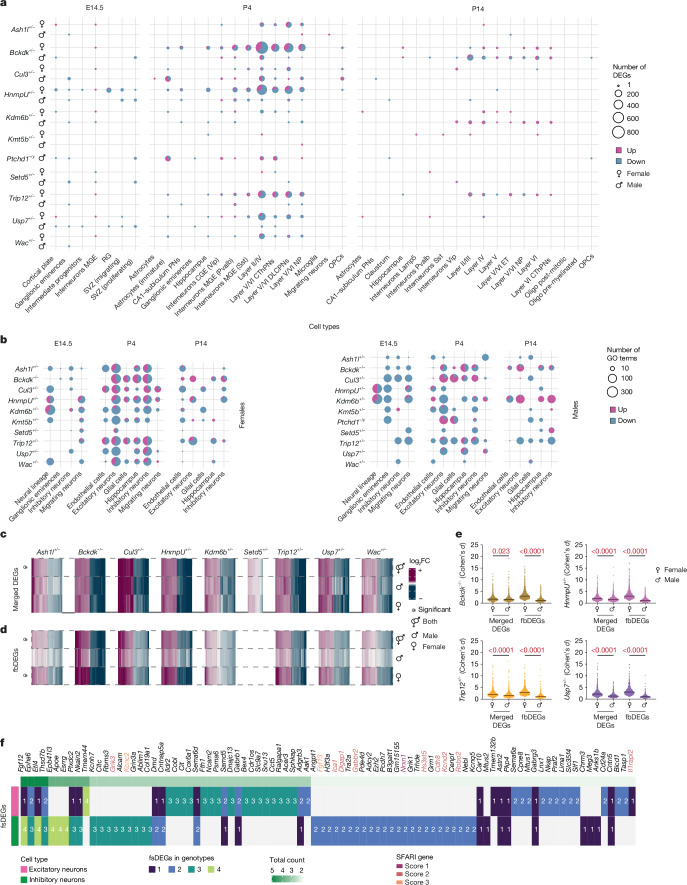
Sex-specific effects across mutants linked to ASD. **a**, Number of DEGs (adjusted *P* < 0.05) in female and male mutants versus sex-matched colony-matched controls. Statistical significance was assessed using two-sided Wald test in DESeq2 with Benjamini–Hochberg correction for multiple testing. **b**, Number of Gene Ontology terms identified by GSEA (FDR < 0.05) across time points, split by sex. The colours indicate the normalized enrichment score direction. Gene Ontology terms were simplified to remove redundancy; all sub-ontologies (Biological Processes, Molecular Function and Cellular Component) are shown. GSEA was performed using clusterProfiler, with a Kolmogorov–Smirnov-like statistic, permutation-based testing, and Benjamini–Hochberg correction (Methods). **c**,**d**, Heatmaps of fold changes for representative merged DEGs (**c**) and fbDEGs (**d**) in P4 excitatory neurons. **e**, Effect sizes for merged DEGs and fbDEGs in females and males. fbDEGs show larger effect sizes in females, whereas merged DEGs show comparable effect sizes between sexes (one-way ANOVA and Sidak’s multiple comparison test). Statistically significant values are shown in red. Black lines indicate the median. **f**, Shared fsDEGs across mutants in P4 excitatory and inhibitory neurons. ASD risk genes are highlighted by the SFARI gene score. Animals used for analyses: *n* (females; E14.5) = 4 (*Cul3*^*+/−*^, *Kdm6b*^*+/−*^ and *Trip12*^*+/−*^) and 3 (*Ash1l*^*+/−*^, *Bckdk*^*−/−*^, *HnrnpU*^*+/−*^, *Kmt5b*^*+/−*^, *Setd5*^*+/−*^, *Usp7*^*+/−*^, *Wac*^*+/−*^ and colony-matched controls); *n* (males; E14.5) = 3 per genotype; *n* (females and males; P4) = 3 per genotype; *n* (females; P14) = 4 (*Ash1l*^*+/−*^, *Cul3*^*+/−*^ and colony-matched controls), 3 (*Bckdk*^*−/−*^, *HnrnpU*^*+/−*^, *Kdm6b*^*+/−*^, *Kmt5b*^*+/−*^, *Trip12*^*+/−*^, *Usp7*^*+/−*^ and *Wac*^*+/−*^) and 2 (*Setd5*^*+/−*^); *n* (males; P14) = 4 (*Ash1l*^*+/−*^, *Cul3*^*+/−*^ and *Setd5*^*+/−*^) and 3 (*Bckdk*^*−/−*^,* HnrnpU*^*+/−*^, *Kdm6b*^*+/−*^, *Kmt5b*^*+/−*^, *Ptchd1*^*−/y*^, *Trip12*^*+/−*^, *Usp7*^*+/−*^, *Wac*^*+/−*^ and colony-matched controls). Source data

Next, we explored the effect of ASD-related mutations on gene transcript levels by splitting sexes (Fig. 5 and Supplementary Fig. 2). Gene expression changes in mutants relative to sex-matched controls differed markedly by sex. At P4, *Ash1l*, *Bckdk*, *HnrnpU*, *Trip12*, *Usp7* and *Wac* mutants showed more DEGs in females than in males (Fig. 5a), whereas the other strains showed minimal sex-dependent effects. For example, combined data from males and females indicated comparable DEG numbers in *Cul3* and *Trip12* mutants, but sex-stratified analysis revealed that *Trip12* haploinsufficiency predominantly affected females, whereas *Cul3* mutants showed similar DEG numbers in the two sexes. Sex differences were generally weaker at E14.5 and P14, except for *HnrnpU*^*+/−*^ and *Trip12*^*+/−*^ females, which consistently had more DEGs at E14.5 and P14, respectively. Conversely, at P14, *Bckdk*^*−/−*^ and *Kdm6b*^*+/−*^ displayed stronger responses in males.

Next, we performed a sex-stratified GSEA, mitigating variance-driven differences in *P* values. At E14.5, most mutants showed changes in cell adhesion, chemotaxis and neuronal maturation in ganglionic eminence cells in males but not in females (Fig. 5b and Supplementary Tables 11 and 12). At P4, GSEA confirmed stronger effects in females, especially in neurons. However, differences were less pronounced than at the DEG level, as most mutants also showed numerous affected Gene Ontology terms in males (Fig. 5b). Exceptions at P4 were *Ash1l*, *HnrnpU* and *Wac* mutants, which consistently exhibited more affected processes in females. *Cul3*^*+/−*^ animals displayed balanced patterns in neurons, whereas glial cells were more affected in males (Fig. 5b). At P14, sex differences were minimal, with overall balanced effects.

To investigate the origins of the sex differences at P4, we first analysed transcripts identified as DEGs in the combined-sex analysis, but not in sex-stratified analyses (approximately 61% of DEGs). We called these genes ‘merged DEGs’. Although not significant in the sex-specific analysis, these genes showed comparable trends in both sexes (Fig. 5c), suggesting shared effects that failed to reach statistical significance when analysing sexes separately due to insufficient statistical power. Examples include mutant-associated genes, such as *Bcat1* and *Slc7a5* in *Bckdk*, *Ccnd2* and *Sema5a* in *HnrnpU*, and *Mdh1* and *Lactb* in *Trip12*.

Subsequently, we explored overlaps of DEGs in female and male mice. In most instances, DEGs in males were also DEGs in females of the same genotype and cell type, independently confirming these changes. Conversely, many of the DEGs in female mice were not significant in male mice. We categorized these genes into two groups: (1) DEGs significant only in females with similar expression trends in both sexes, termed female-biased DEGs (fbDEGs; Fig. 5d), and (2) DEGs significant only in females but exhibiting no or opposite expression changes in males, referred to as female-specific DEGs (fsDEGs; Supplementary Table 13).

Next, we compared standardized effect sizes (Cohen’s *d* values) of fbDEGs between females and males (Fig. 5e). Unlike merged DEGs, which showed similar effect sizes in both sexes, fbDEGs consistently exhibited higher effect sizes in females. In addition, fbDEGs showed larger average effect sizes in females than merged DEGs in either sex, indicating that sex differences are driven by stronger effects in females (Fig. 5e and Supplementary Note).

Finally, we examined fsDEGs (Fig. 5f). *Wac* mutants showed the highest proportion of fsDEGs. Of note, *WAC* has recently been identified as a female-specific candidate gene^55^, suggesting sex-specific effects and potentially greater disease penetrance in female individuals.

Several fsDEGs were shared across mutants linked to ASD and cell subclasses (Fig. 5f). These were enriched for synaptic functions (*q *= 6.5 × 10^−7^), ion channels (*q* = 4.8 × 10^−6^) and cell adhesion (*q* = 7.9 × 10^ −3^). Some fsDEGs have been previously implicated in sex-specific effects. For example, deletion of *Grik1*, an fsDEG in inhibitory neurons of *Bckdk* and *HnrnpU* mutants, results in sex-divergent phenotypes in mice^56^. *ApoE* was downregulated specifically in female interneurons in *Ash1l*, *Bckdk*, *Trip12* and *Usp7* mutants. *ApoE*, an Alzheimer’s disease risk gene, has been associated with sex differences in ASD^57–60^.

In summary, mutations in several genes associated with ASD elicit sex-specific effects, with generally higher effect sizes in female mutant mice.

## Restricted cerebellar effects in mutants linked with ASD

**Fig. 6:**
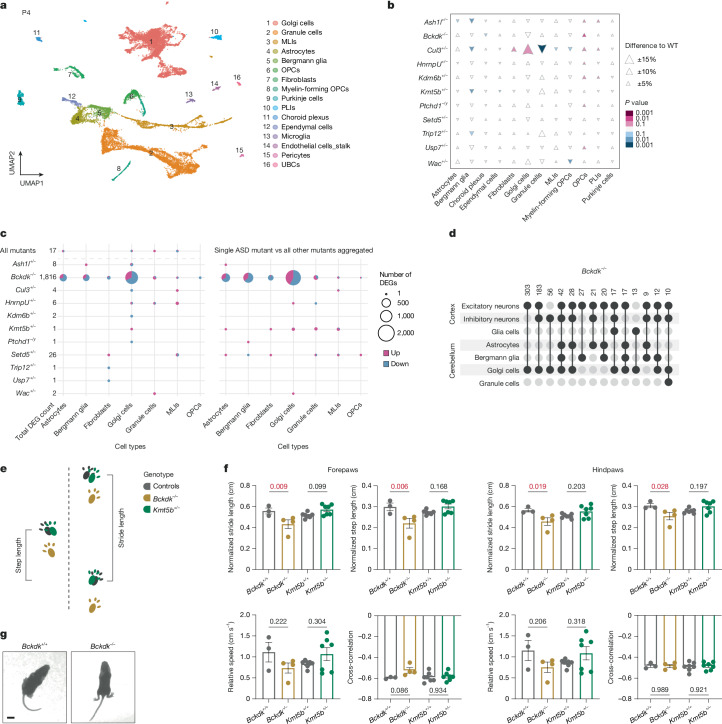
Multi-omics sequencing of perinatal cerebellar tissue. **a**, UMAP of integrated snRNA-seq and snATAC-seq data from male P4 cerebellum, coloured by cell-type annotation. MLI, molecular layer interneuron; PLI, Purkinje layer interneuron; UBC, unipolar brush cell. **b**, Changes in cell-type abundance across mutants. The arrowheads represent relative percentage differences versus colony-matched controls (WT). Statistical significance was assessed in edgeR using a two-tailed quasi-likelihood *F*-test. Nominal *P* values are shown. *n* = 3 per genotype. **c**, Number of DEGs (adjusted *P* < 0.05) across mutants and cell types. All mutants denotes mutants analysed collectively compared with colony-matched controls. Total DEGs per cell type per time point, counting repeated DEGs once, are shown. DEGs (adjusted *P* < 0.05) identified by comparing each mutant to all other mutants combined is also displayed (right). Statistical significance was assessed using two-sided Wald test in DESeq2 with Benjamini–Hochberg correction for multiple testing. **d**, Overlap of DEGs between the *Bckdk-*mutant cortex and cerebellum at P4 (1,023 shared genes between indicated cell types). **e**, Schematic of gait patterns in juvenile (P13–15) *Bckdk* and *Kmt5b* mutants and littermate controls. **f**, Altered gait in *Bckdk*^*−/−*^ mice, including reduced normalized stride and step length of forepaws and hindpaws (top), reduced stride speed and mild forepaw coordination deficits assessed as cross-correlation during walking (bottom; *n* = 3 (*Bckdk*^*+/+*^), 4 (*Bckdk*^*−/−*^) and 7 (*Kmt5b*^*+/+*^ and *Kmt5b*^*+/−*^). Data are mean ± s.e.m.; one-way ANOVA with Sidak’s multiple comparison test. Statistically significant values are shown in red. **g**, Abnormal hindpaw positioning during stride cycles in *Bckdk*^*−/−*^ mice versus littermate controls. Scale bar, 1 cm. Source data

To investigate whether mutations associated with ASD lead to changes in other brain regions, we analysed gene expression in the cerebellum (Fig. 6a and Supplementary Note), a region associated with motor coordination and cognitive functions that has been shown to be affected in individuals who have autism^61,62^.

First, we assessed cerebellar cell-type abundance to assess lineage progression in mutants. Compared with WT, only *Cul3* mutants exhibited differences, with a trend towards increased Golgi cell and reduced Granule cell numbers (Fig. 6b).

We then investigated cerebellar gene expression at P4. Pseudobulk analysis revealed no shared transcriptional changes across all mutants. However, analysing each mutant separately revealed widespread gene expression changes in *Bckdk* mutants, affecting glial cells as well as excitatory and inhibitory neurons (Fig. 6c and Supplementary Tables 3 and 4). To exclude potential control-specific effects, we compared each mutant against all other mutants collectively (Fig. 6c, right). This confirmed the distinct transcriptional profile of *Bckdk* mutants, with limited changes in the others. Transcripts affected in the cerebellum of *Bckdk* mutants largely matched those altered in the cortex, suggesting consistent pathway disruption across regions (Fig. 6d).

GSEA confirmed significant shifts in amino acid and metabolic pathways in *Bckdk* mutants, particularly affecting neurons and, more specifically, Golgi cells, consistent with the cerebral cortex. Other genotypes displayed only few changes, with some showing variations in synaptic protein expression (Supplementary Table 6).

Given the role of the cerebellum in motor coordination, we examined motor behaviour in juvenile (P13–15) *Bckdk*^*−/−*^ mice and compared them with *Kmt5b*-mutant mice (Fig. 6e), which exhibited only one DEG in the cerebellum. *Bckdk* mutants had motor impairments, including reduced speed, shorter stride and step lengths, and a trend towards reduced cross-correlation of forepaw movements during the walking phase (Fig. 6f and Supplementary Videos 2 and 3). These mice also showed abnormal hindpaw positioning during stride cycles compared with WT littermates (Fig. 6g). By contrast, *Kmt5b* mutants showed no motor behaviour abnormalities at the same age.

Overall, mutations linked with ASD did not broadly alter cerebellar gene expression at P4, except in *Bckdk* mutants, which also showed motor deficits at this stage. In the cerebellum, *Bckdk* mutation effects mirror those in the cerebral cortex, predominantly influencing metabolic and synaptic functions in neuronal cells.

## Discussion

By testing multiple ASD models across development, our study highlights the dynamic nature of ASD-related changes and outlines a coherent trajectory in which diverse mutations linked with ASD impinge on common, stage-bound programs, such as delayed RG progression and early postnatal downregulation of ion channel or synaptic modules, while preserving genotype-associated signatures that intensify with maturation. Network localization confirms that convergence is strongest within early development but weakens by P14. Functionally, transcriptome–electrophysiology coupling and minimal conductance modelling suggest that small shifts in ion channel expression are sufficient to produce a diverse range of phenotypes. Collectively, our results motivate stage-aware and sex-aware intervention strategies, while also raising the question of whether intervention targeting shared early developmental changes can redirect later disorder trajectories (Supplementary Discussion).

## Methods

### Animals

All animals used in this study were approved by the Institutional Animal Care and Use Committee at ISTA and by the Bundesministerium für Bildung, Wissenschaft und Forschung, Austria (approval numbers BMWFW−66.018/0008-WF/II/3b/2014, BMWFW-66.018/0012-WF/V/3b/2015, BMWFW-66.018/0012-WF/V/3b/2017, BMWFW-66.018/0015-V/3b/2019, BMWFW-66.018/0032-V/3b/2019, BMBWF-V/3b/2020-0.342.159, BMBWF-V/3b/2020-0.148.791, BMBWF-V/3b/2021-0.291.172, BMBWF-V/3b/2021-0.291.177, BMBWF-V/3b/2022-0.292.788 and BMBWF-V/3b/2022-0.121.445). All our mutant lines were maintained in a C57BL/6J background. To maintain this background, colonies are continuously refreshed by mating mutants with C57BL/6J animals drawn from a continuously refreshed central C57BL/6J colony. All experiments were performed on mice ranging from E14.5 to P4 and P14. Embryonic time points were determined by plug checks after timed matings, defining E0.5 as the morning post-coitum. Animals were kept in the Preclinical Facility at ISTA, housed in commercially available individually ventilated cages under defined standard laboratory conditions (room temperature 22 ± 1 °C, relative humidity 55 ± 10%) on a 12-h light–dark cycle (lights on at 07:00). Animals were housed in groups of 3–4 animals per cage, with food and water available ad libitum. Experiments were carried out under specific pathogen-free conditions, and the health status of the mouse lines was routinely checked by a veterinarian. All transgenic mouse lines were backcrossed into a C57BL/6J background a minimum of two times. Both females and males were used for experiments.

### Generation of constitutive heterozygous mouse lines

Constitutive heterozygous mice (that is, *Ash1l*^*+/−*^, *Kdm6b*^*+/−*^, *Kmt5b*^*+/−*^, *Trip12*^*+/−*^, *Usp7*^*+/−*^ and *Wac*^*+/−*^) were generated within the Transgenic Unit at IST Austria in a C57BL/6J background by zygote electroporation of the Cas9 protein (634621, TakaraBio) and custom-made sgRNAs. To generate *Ash1l*^*+/−*^ mice, one sgRNA (CAATCACCATTCCGCTATCC AGG) targeting exon 12 of *the Ash1l* gene was used. For *Kdm6b*^*+/−*^, two sgRNAs (CCCAAGGACCCGAGGTGATA GGG and TACGTATGAGGAGCGAACCC TGG) targeting intronic regions flanking exon 19 of the *Kdm6b* gene were used. For *Kmt5b*^*+/−*^, two sgRNAs (CCCTCACGACCCTACTACTG TGG and TTTACGTCTCAAGTCACACT GGG) targeting intronic regions flanking exon 9 of the *Kmt5b* gene were used. Similarly, to generate *Trip12*^*+/−*^ mice, two sgRNAs (AGCGTATTCTCACTTTGATC TGG and TAAATTTGTAGAGACAGTTA AGG) targeting intronic regions flanking exon 3 of the *Trip12* gene were used. Similarly, for *Usp7*^*+/−*^, two sgRNAs (CTCTCCCGAACATTATGAAG AGG and TGCCACGTCTTATACACATT AGG) targeting intronic regions flanking exon 6 of the *Usp7* gene were used. To generate *Wac*^*+/−*^ mice, one sgRNA (ATGTCGGAGGCGCAGATGTA GGG) targeting exon 8 of the* Wac* gene was used.

Superovulation was performed to produce large numbers of zygotes. In brief, 3–4-week-old donor females (in-house) were hormone primed by intraperitoneal injection of 5 IU of pregnant mare serum gonadotropin. Of human chorionic gonadotropin, 5 IU was administered at 46–48 h after pregnant mare serum gonadotropin treatment, followed by 1:1 mating with stud males (in-house). Zygotes were isolated from the oviduct of mated females at 0.5 days post-coitum. M2 with hyaluronidase (MR-051-F, Sigma) was used to dissociate cumulus cells. Zygotes were then cultured in small drops of KSOM (MR-121-D, Sigma) under mineral oil (M-5310, Sigma) at 37 °C and 5% CO_2_, until the electroporation. Electroporation of mouse embryos was performed similarly as previously described^63^ with the adaptation of using Opti-MEM (31985062, Thermo Fisher) instead of PBS. Finally, F0 founder mice were genotyped using standard PCR amplification with primers 500 bp to 1 kb away from guide RNA-binding sites. F1 mice were generated by backcrossing founder mice with C57BL/6J animals. The F1 mice were again genotyped by PCR, and positive amplicons covering the deleted sequence were Sanger sequenced to confirm precise targeting. All transgenic mouse lines were back-crossed into a C57BL/6J background a minimum of two times, and predicted off-targets were checked. A list of oligonucleotides used is provided in Supplementary Table 1. The constitutive knockout mouse line (*HnrnpU*^*+/−*^) was obtained by mating *HnrnpU*^*WT/flox*^ (Hnrnpu<tm1.1Tman>/J, strain: #032187, RRID:IMSR_JAX:032187) mice with a *CMV-Cre* line (B6.C-Tg(CMV-cre)1Cgn/J; strain #:006054, RRID:IMSR_JAX:006054) and subsequent backcrossings to C57BL/6J WT animals. Crossing of *HnrnpU*^*WT/flox*^ mice with homozygous animals of the *CMV-Cre*^*Cre/Cre*^ line results in ubiquitous deletion of one allele of *HnrnpU* spanning exons 4–14.

### Single-nucleus multi-omics profiling of gene expression (snRNA-seq) and chromatin accessibility (snATAC-seq) from the same nucleus (snMultiome-seq)

#### Sample preparation and nuclei isolation

All the sequenced mutant animals were generated by mating a C57BL/6J female from the continuously refreshed central C57BL/6J colony with a mutant male maintained on the same C57BL/6J background (for example, C57BL/6J female × *Ash1l*^*+/−*^ male = litter sequenced). The only exceptions were homozygous *Bckdk* mice whose heterozygous breeders were regularly refreshed with the same central C57BL/6J colony. Control animals were C57BL/6J × C57BL/6J offspring sourced from the same central colony. Dams used for mutant and control litters were drawn from the same central-colony cohorts and often littermates; at minimum, they were not separated by generations of inbreeding.

Brain tissue (forebrain and cerebellum) from E14.5, P4 and P14 mice was used for snMultiome-seq experiments. Animals coming from at least three separate litters were collected to avoid potential litter-specific biases. For the embryonic time point (that is, E14.5; *n* = 3 per genotype + sex), timed-pregnant females were decapitated, and embryos were rapidly dissected on ice. Cortical tissue was dissected in ice-cold PBS, meninges were removed, and forebrain structures were snap-frozen in liquid nitrogen and stored at −80 °C until nuclei isolation.

For postnatal time points (that is, P4 and P14; *n* = 3 per genotype + sex + time point), mice were decapitated, the brain was rapidly dissected on ice and subcortical structures were removed. The remaining forebrain (cortex + hippocampus) and cerebellum were separately snap-frozen in liquid nitrogen and stored at −80 °C until nuclei isolation.

Frozen tissue samples were dissociated to generate single-nuclei gene expression libraries following a modified protocol from 10X Genomics (Chromium Nuclei Isolation Kit CG000505, Rev A). Buffers were supplemented with RNase inhibitors (provided by 10X Genomics and 3335402001, Sigma). In brief, 200 µl of lysis buffer was added to the sample and dissociated with pestles provided by the vendor. After adding additional 300 µl of lysis buffer, the tissue was homogenized by pipette mixing and incubated on ice for 10 min. For the P14 forebrain, an additional 500 µl of EZ lysis buffer (NUC101, Sigma) was added to the samples to prevent clogging of the nuclei isolation columns. Afterwards, 500 µl of the cell suspension was loaded onto nuclei isolation columns and centrifuged for 20 s at 16,000 rcf at 4 °C, followed by vortexing the flow-through for 10 s and centrifugation for 3 min at 500 rcf at 4 °C in a swinging bucket rotor. The supernatant was removed, and the pellet was gently resuspended in 500 µl of debris removal buffer before centrifugation for 10 min at 700 rcf at 4 °C. After removal of the supernatant, the pellet was washed with 1 ml of wash buffer followed by centrifugation for 5 min at 500 rcf at 4 °C. For multiplexing, six samples were pooled after barcoding with CMOs (sequences of all CMOs used are provided in Supplementary Table 1). Each sample pellet was resuspended in a total of 180 µl of wash buffer containing 0.2 µM unique CMO anchor–barcode, vortexed for 3 s and incubated on ice for 5 min. CMO co-anchor was added to all samples of a multiplexing group with a final concentration of 0.2 µM, vortexed for 3 s and incubated on ice for 5 min. After adding 1 ml of wash buffer, samples were centrifuged for 5 min at 500 rcf at 4 °C. Depending on the tissue size, one (embryonic, P4 cortex and cerebellum) or two (P14 cortex) additional washing steps were performed. After removing the remaining supernatant, the pellet was resuspended in 50 µl of resuspension buffer. Nuclei were counted by flow cytometry (BD LSRFortessa) with Precision Count Beads (424902, BioLegend), and samples were diluted to obtain approximately 4,000 nuclei per sample, resulting in a total of 20,000 nuclei per lane of a 10X Chromium Controller (PN-1000204). GEMs were generated using Chromium Next GEM Chip J Single Cell kit (PN-1000230), Chromium Next GEM Single Cell Multiome GEM Kit A (PN-1000232) and Chromium Next GEM Single Cell Multiome Gel Bead Kit A (PN-1000231) as instructed by the manufacturer.

#### Library preparation and snMultiome sequencing

Library preparation was carried out according to the manufacturer’s protocol (Chromium Next GEM single cell multiome ATAC + gene expression, CG000338, Rev F) with the following modifications regarding the multiplexing reactions: during cDNA amplification, 0.2 µM ADT additive primer (5′-CCTTGGCACCCGAGAATTCC-3′) was added to the reaction. During cDNA cleanup, 120 µl of supernatant was saved to purify Hash-DNA using a 2.0× ratio of SPRIselect bead volume (B23319, Beckman Coulter) to sample volume. Hashtag libraries were generated using 5 ng of Hash-DNA, 1X KAPA2G Fast HotStart Readymix (7958935001, Roche) and unique combinations of 0.4 µM i5 and i7 indices (sequences are provided in Supplementary Table 1). Following amplification (98 °C for 2 min, 10 times (98 °C for 20 s, 54 °C for 30 s, 72 °C for 20 s), 72 °C for 5 min, 4 °C hold), libraries were cleaned up using a 1.2× ratio of SPRIselect bead volume (B23319, Beckman Coulter) to sample volume. Library quality and size were assessed with the High Sensitivity DNA Analysis Kit (5067-4626, Agilent Technologies) and the Bioanalyzer 2100. Libraries were sequenced by the Biomedical Sequencing Facility at the CeMM Research Center for Molecular Medicine of the Austrian Academy of Sciences, using the Illumina NovaSeq 6000 platform. For gene expression, libraries were pooled in a ratio of 83% RNA:17% HTO and sequenced on Illumina NovaSeq6000 systems using the sequencing format: 28 cycles for read 1, 10 cycles for i7, 10 cycles for i5 and 90 cycles for read 2. For snATAC-seq, libraries were sequenced on Illumina NovaSeq6000 systems using the sequencing format: 50 cycles for read 1N; 8 cycles for i7, 24 cycles for i5 and 49 cycles for read 2N.

### Single-nucleus muli-omics data analysis

#### Pre-processing and demultiplexing, initial analysis and clustering

Samples used for multi-omics analyses were demultiplexed, and the raw sequencing data were converted to FASTQ format using bcl2fastq (v2.20.0.422). Joint processing of the gene expression and ATAC modalities was performed using the cellranger-arc software (v2.0.0). The mm10 reference genome was used for the alignment (refdata-cellranger-arc-mm10-2020-A-2.0.0, obtained from https://cf.10xgenomics.com/supp/cell-arc/refdata-cellranger-arc-mm10-2020-A-2.0.0.tar.gz). To capture CMO reads, the gene expression modality was separately processed using the cellranger count pipeline (v7.0.0 and v7.1.0), aligning it against the mm10 reference genome (refdata-gex-mm10-2020-A, obtained from https://cf.10xgenomics.com/supp/cell-exp/refdata-gex-mm10-2020-A.tar.gz). Each nucleus was assigned to the sample of origin as part of further demultiplexing, for which a Gaussian mixture model was fitted to the CMO count distributions. The probability that a given CMO read count was derived from background or positive signal was then inferred (as previously described^64^). The resulting multiome data was further analysed in R (v4.3.0) or Python (v3.10.5) using Seurat^65^ (v4.3.0.9012) and Signac^66^ (v1.10.0). For reproducibility, the analysis was implemented as targets^67^ (v1.2.2) pipeline using renv (v1.0.2).

Pre-processing was done per 10X lane (that is, per multiplexing group of 4–6 samples). Peaks for the snATAC-seq modality were called for each run separately using MACS2 (v2.2.7.1) within Signac. A set of consensus peaks for the snATAC-seq analysis was created according to Signac. CMO demultiplexing information was taken from Cellranger output and added as metadata.

Doublets were identified using scDblFinder^68^ (v1.14.0), separately for snRNA-seq and snATAC-seq modality. snRNA-seq parameters for the scDblFinder function were dims = 50, cluster = TRUE and knownUse = ‘discard’. snATAC-seq features were first aggregated (aggregateFeatures function, *k* = 500) and parameters for scDblFinder were dims = 50 and aggegateFeatures = TRUE. Cells identified as doublets based on CMO data were included as known doublets (knownDoublets parameter). Quality control metrics such as the percentage of mitochondrial reads, nucleosome signal and transcription start site enrichment were calculated per run. After removing known and estimated doublets, outlier thresholds for snRNA-seq read counts (nCount_RNA) or snATAC-seq read counts (nCount_ATAC) were derived using the isOutlier function from the scuttle package^69^ (v1.10.2) with parameters nmads = 3, type = ‘both’ and log = TRUE. Nuclei falling outside these thresholds or with more than 5% of mitochondrial reads were removed.

#### Integration into combined analysis

Complete datasets for each time point or tissue were created by integrating individual Seurat objects representing separate 10X lanes (that is, multiplexing groups). snRNA-seq data were normalized using SCTransform^70,71^ from Seurat with parameter vst.flavor = ‘v2’. Integration of normalized snRNA-seq data was performed according to Seurat using the reciprocal principal component analysis (PCA) method with the following non-default parameters: SelectIntegrationFeatures(nfeatures = 2,500), FindIntegrationAnchors(reduction = ‘rpca’) and IntegrateData(dims = 1:50). Integration of snATAC-seq data was performed at the level of the LSI embedding using the IntegrateEmbeddings(dims.to.integrate = 1:50) function with the integration anchors derived from snRNA-seq data. Identification of cell types was performed using the combination of both modalities: snRNA-seq and snATAC-seq. A PCA embedding was derived for the integrated snRNA-seq data, the weighted nearest neighbour graph was calculated using the FindMultimodalNeighbors function with the integrated LSI and integrated PCA reductions as input and dims_atac = 2:50 and dims_rna = 1:50. The resulting weighted nearest neighbour graph was used for UMAP visualization of the dataset and identification of clusters using the FindClusters function with parameters algorithm = 3 (smart local moving algorithm), resolution = 0.5 (forebrain and cortex) or 0.3 (cerebellum). Cells designated as outliers per cell cluster were designated as such and removed using the Mahalanobis distance (more than 100) calculated from genes expressed in more than 80% of the cells.

#### Label transfer and cell-type annotation

To support cluster annotation, reference datasets for each tissue and time point were used (a list of respective references used is provided in Supplementary Table 1). Seurat objects were created from all datasets except where data were provided in this form. Normalization and dimensionality reduction were performed in alignment with the published studies where possible. Cell-type annotation of clusters was done in multiple steps. Label transfer from multiple published datasets (see above) was used for automated annotation of clusters using Seurat. Seurat’s FindMarkers was used to determine marker genes for each cluster. Cluster annotations were manually validated afterwards. Here information from different reference datasets, in addition to the expression of canonical marker genes, was validated. Clusters annotated as striatal cell types were excluded from further analyses due to sample preparation variabilities, that is, brain region adjacent to the forebrain, and the presence or absence thereof is highly variable. To reduce the complexity of downstream analyses, clusters belonging to the same cell-type annotation were merged. To avoid merging disparate clusters, differential expression (using FindMarkers of subgroups of clusters) was used as a reference.

#### Cell variability

Single-cell gene expression heterogeneity within each cell-type cluster and mutant was tested and visualized using coefficient of variation histogram plots and permutation testing. Permutation testing refers to the number of genes designated as significantly different between (1) cells of the same transgenic mouse line or the C57BL/6J WT mice, or (2) cells of different transgenic mouse lines or the C57BL/6J WT mice. Twelve cells were randomly sampled from the respective population (or populations) two times, and differences in expression values were obtained. Any difference value that was obtained from less than 10 cells with an expression value was discarded. Then, two such differences in expression value sets (either for (1) or (2) were compared with a *t*-test and deemed to have a high variability when their *P* value was below 0.05. This was repeated ten times. Furthermore, differences were also calculated between cell-type clusters of one transgenic mouse line and the C57BL/6J WT mice.

#### Differential abundance analysis

Differential compositional analysis of cell abundances between all mutants linked with ASD collectively and WT mice was conducted using the scCODA Python package^72^ (v0.1.8), using Hamiltonian Monte Carlo sampling. The reference cell types used were as follows: layer I (Cajal–Retzius) neurons for the cerebral cortex at E14.5, CGE interneurons (interneurons CGE (Vip)) for the cerebral cortex at P4, interneurons Sncg (Vip) for the cerebral cortex at P14 and choroid plexus cells for the cerebellum at P4. The credibility of the effects was evaluated at two FDR thresholds: 0.05 and 0.01. Differential abundance of cell-type clusters across individual mutants linked with ASD was also tested with edgeR^73^ (v3.42.4) using absolute cluster abundances in cells per sample as input. For cortical time points, the model included sex as a covariate.

#### Pseudotime analysis

To reconstruct the differentiation trajectory from RG to the cortical plate cells at E14.5, we created an embedding based on the transcriptomic profiles using the Python package Harmony^74^ (harmonypy, v0.0.5) for batch correction. After that, Palantir^75^ (v1.0.1) was used to generate a latent representation that preserves the pseudotime ordering of cells. Utilizing the first five components of this representation, the SimplePPT algorithm^76,77^ was applied to learn the principal tree that captures the differentiation process. Subsequently, the scFates tool^78^ (v1.0.6) was used to map this principal tree back onto the constructed UMAP using Harmony-corrected PCA coordinates. Pseudotime values for each cell were assigned by projecting the cells onto the principal tree. The RG cell cluster was defined as the root of the trajectory, progressing towards cells of the cortical plate. The pseudotime analysis included RG cells, subventricular zone migrating cells, intermediate progenitors and cortical plate cells.

#### Pseudobulk differential gene expression analysis

Differential gene expression between genotypes within cell-type clusters was performed on pseudobulk data. Either (1) all female mice only, (2) all male mice only, or (3) all female and male mice without sex indicators were used for testing between one transgenic mouse line and the C57BL/6J WT mice.

Pseudobulk profiles per cluster and per sample were derived using the Libra package (v1.0.0). Mitochondrial genes were removed, and only genes with at least 15 counts in at least two samples (single sex) or 30 counts in at least two samples (pooled sexes) were included. DESeq2 (ref. ^79^) (v1.40.2) was used to test for differential gene expression using test = ‘Wald’, fitType = ‘local’ and an adjusted *P* value threshold of 0.05. The model included sex as a covariate for the analysis of the forebrain and cortex. Shrunken fold changes for downstream rank-based analyses were calculated using the apeglm method^80^ implemented in the lfcShrink function from DESeq2. The tests involved three comparison types: (1) comparing each single mutant genotype to WT controls, (2) comparing each of the single mutant genotypes to all other mutant genotypes (excluding WT controls), and (3) comparing the WT mice to all mutants collectively. Composite differential expression analysis was carried out by comparing gene names designated as differently expressed (DESeq2) and organizing these as Venn diagrams. Furthermore, a leave-one-out cross-validation strategy, which systematically removed one of the C57BL/6J WT mice from the DESeq2 comparison, was used to investigate whether any of the WT mice were a strong outlier. To assess differences between female and male WT controls, WT controls were also compared with a similar statistical setup as described above using pseudobulk differential gene expression analysis. Here, instead of adjusted *P* values, nominal *P* values were used to assess the extent of the sex differences with a critical α-threshold of 0.05.

To evaluate the statistical significance of overlap in differential gene expression patterns between mutants, each mutant pair was compared against distinct sets of C57BL/6J control samples. Control samples (including both male and female samples) were randomly divided into two disjoint groups. For each mutant pair, differential expression analysis was performed in DESeq2 using the same model as described above (‘~group + sex’), except for *Ptchd1* mutants, where all samples were male and the model ‘~group’ was used. Comparison of each mutant to a distinct control set ensured statistical independence between the two comparisons. Overlap between each mutant pair was assessed by comparing the top 300 most DEGs (ranked by *P* value), and the observed-to-expected ratio and statistical significance of the overlap were determined using Fisher’s exact test. Only genes with a minimal expression level (baseMean ≥ 1) in at least one mutant of a given pair were included in the analysis.

To compare the extent of transcriptional differences across mutants and cell types, we used a downsampling-based differential expression analysis aimed at balancing statistical power. Pseudobulk profiles were generated for each genotype–cell type combination and downsampled to 10,000 molecules per profile. Profiles with fewer than 95% of this target were discarded, and only combinations with at least five valid samples were included in the analysis. Differential expression testing was performed with DESeq2 using the design formula (‘~genotype + sex’). To mitigate stochastic effects from subsampling, we conducted 100 independent downsampling iterations, and for each mutant–cell type comparison, we reported the average number of genes with an adjusted *P* < 0.05.

#### Gene Ontology analysis and GSEA

Gene Ontology term analysis was performed using the enrichGO function from the clusterProfiler package^81^ (v4.8.2) using the list of DEGs using a cut-off *P* < 0.01 and cut-off *q* < 0.05.

GSEA was performed as previously described^82^, using the gseGO function from the clusterProfiler package^81^ (v4.8.2) with default parameters except minGSSize = 100 and ont = ‘ALL’ using a cut-off *P* < 0.05. All genes were used as input for clusterProfiler and the list of genes was sorted by log_2_FC using shrunk fold changes using the lfcShrink function. Enrichment was assessed using a Kolmogorov–Smirnov-like statistic with permutation-based significance testing. Benjamini–Hochberg FDR correction of *P* values was used for both analyses.

To remove redundancy in enriched gene ontologies, the simplify-method function from clusterProfiler was used to visualize enriched gene sets (Figs. 2b and 5b).

We performed Gene Ontology term enrichment analysis on genes associated with differential accessibility at the gene-body level. DAR (*P* < 0.01) per cell type were enriched with Gene Ontology terms using the clusterProfiler R package (using the Biological Process ontology, using a cut-off *P* < 0.01 and a cut-off *q* < 0.05. Enriched terms were illustrated using the enrichplot R package (v1.20.1) with the dotplot function.

#### Constructing enrichment maps using Cytoscape

To visualize the results of enriched gene sets and pathways for mutants linked with ASD, the EnrichmentMap plugin^83^ (v3.4.0) for Cytoscape (v3.10.1) was used. Enriched gene sets for excitatory neurons were loaded into the EnrichmentMap plugin with an FDR cut-off value of 0.1. Gene sets were not filtered, setting no minimum experimental cut-off (Extended Data Fig. 4).

#### Summarizing of Gene Ontology terms using REVIGO

REVIGO^84^ was used to consolidate GSEA terms into a reduced set of parent terms. For this, a comprehensive list of all GSEA terms, irrespective of time point or genotype, was constructed and uploaded. The parameters ‘small (0.5)’ and ‘remove obsolete GO terms’ were used, resulting in robust and comprehensive clustering. Subsequently, all detected GSEA terms were mapped to their new parents. The parent terms were organized in subclusters of circular heatmaps, which were implemented with the circlize (v0.4.15) R package^85^. Representative terms were highlighted in the corresponding figures showing circular heatmaps.

#### Synaptic function and localization analysis using SynGO

Synaptic Gene Ontology (SynGO)^86^ (v1.2) was used to identify types of known synaptic proteins (in presynaptic and postsynaptic compartments) and different functional classes. Lists of DEGs of the respective mutants linked with ASD of P4 and P14 excitatory neurons were used as input to investigate overrepresented synaptic terms. Biological processes were selected for enrichment.

#### Differential accessibility

We conducted pseudobulk testing using the DESeq2 R package^79^ (v1.34.0) to identify peaks that differ between ASD mutant and WT mice. Given the low signal-to-noise ratio in scATAC-seq experiments^87^ and the high number of comparisons, we used an uncorrected *P* value threshold of 0.001 to include peaks in the list of DARs. Motif enrichment analysis was performed on a set of downregulated peaks using the SnapATAC2 Python package (v2.3.0), with binomial testing and Benjamini–Hochberg FDR correction of *P* values. We tested for correlations between the gene-body differential accessibility *P* values and the corresponding gene lengths. Our analysis revealed no significant association (<0.1), demonstrating that gene length is not a systematic confounding factor.

### Network-based analyses

#### Generation of reference protein–protein interaction network

Several sources were merged to generate the protein–protein interaction network (PPI) used in this study. In brief, interaction data for mouse was downloaded from MIPPIE^88^, IntAct^89^, STRING^90^, Reactome^91^ and BioGRID^92^. For STRING, only physical interactions were used. The BioGRID database was downloaded separately, as newer releases were not integrated in the MIPPIE database. In addition, we incorporated mouse-brain-specific interaction data from Skinnider et al.^93^ to ensure coverage of target tissue-specific interactions. The resulting PPI comprises 18,921 proteins with 417,767 undirected interactions.

#### Network colocalization of DEGs

Raw count data were log normalized using a library size normalization $\log (\frac{x}{\mathrm{libary}}\times 10,000+1)$. DEGs from pseudobulk differential gene expression analysis of all 11 mutant lines were evaluated for similarity in their underlying PPIs using a modified version of a previously published method^94^ applied to our reference PPI network. Key modifications include the use of a symmetric random walk, which inherently corrects for degree distribution^95^, and an extension of the original tied diffusion algorithm^96^ to handle more than two random walks. In brief, for each genotype and time point, we performed symmetric random walks using the pseudobulk DEGs as seed nodes. For each walk, network nodes were ranked in descending order according to their visiting probability. An aggregated rank for each node was then computed as the geometric mean of its ranks across all walks, referred to as the rank product^97^. Only nodes with a rank product below 3,000 were retained for further analysis. This approach builds on NetColoc, originally designed to compare pairs of diseases using the tied diffusion algorithm introduced by Paull et al.^96^ to identify shared components of molecular networks between two sets of molecules. Although NetColoc was only demonstrated for pairwise comparisons, we argue that the method is not inherently restricted to two sets, as described below. In brief, TieDIE begins by performing a random walk on a common reference interaction network for each set of molecules of interest. Each random walk assigns weights to network nodes according to their proximity to the respective molecules. The shared neighbourhood among the resulting walks is then determined by applying a threshold to a weighting function z=f(R), where *R* denotes the set of random walk outputs and f(.) is an arbitrary function that prioritizes nodes with consistently high weights across walks. To accommodate an arbitrary number of random walks, we adopted a previously published ranking approach developed to identify highly ranked DEGs across multiple differential expression analyses^97^. Specifically, our ranking function is the rank product across all random walks in *R*, calculated as the geometric mean of the rank for each node across the walks.

#### Computing gene systems from colocalized genes

Colocalized gene sets were grouped into clusters of genes with similar molecular functions, referred to as gene systems, using HiDeF^98^. We applied HiDeF to cluster colocalized genes separately for each time point. To focus on more specific systems, we excluded clusters containing more than 250 genes, as such large clusters are often nonspecific artefacts of the hierarchical clustering used by HiDeF. We further removed clusters with a persistence score below 20 or without any overlap with the corresponding seed gene sets. For the remaining clusters, we assessed functional enrichment in GO_BP_2023 (Biological Processes), GO_MF_2023 (Molecular Function), GO_CC_2023 (Cellular Component) and MGI_Mammalian_Phenotype_Level_4_2024 using the ENRICHR API via gseapy^99^. To avoid redundant gene systems across time points, we merged clusters based on their enrichment profiles using hierarchical clustering. The similarity between two clusters was calculated as the jaccard index of the tokenized enrichment terms. If no enrichment was available for either cluster, similarity was computed from the overlap with genes in the system.

#### Gene system–genotype associations

Associations between gene systems and genotypes were assessed using a regression-based method described in Jin et al.^100^. In brief, we first filtered the gene systems to retain only those with at least four genes present in our snRNA-seq dataset, removing any system that fell below this threshold after excluding genes absent from the data. For each time point, we then assessed the association between gene systems and genotypes by fitting regression models of the genotype variables on the module eigengene scores of each system, calculated per cell in the corresponding subset of the snRNA-seq data. The full model was fit in statsmodels (v0.14.3) using the WT as reference, and is specified as follows:

module eigengene score *~ *genotype + nFeature_RNA

The resulting regression coefficients represent the associations between each genotype and gene system relative to WT. To further reduce the number of systems for visualization, we retained only those that (1) were associated with more than 20% of genotype–time point combinations, and (2) exhibited substantial variation across genotypes and time points, defined as having a maximum absolute difference from the median greater than 0.2 across all combinations (genotype + time point).

#### Network overlap computation

We computed pairwise gene set overlaps on the PPI as introduced in Menche et al.^101^. Gene sets were defined for each genotype and time point by ranking genes within the PPI network according to their absolute log_2_FC values from pseudobulk differential expression analysis. Excitatory and inhibitory neurons were analysed separately. Network overlap analyses were then performed for all pairs of genotype–time point combinations following Menche et al.^101^. For each comparison, overlaps of 1,000 randomly drawn gene set pairs were computed to generate a reference distribution. To ensure comparability, we implemented a degree-matched randomization procedure by binning PPI nodes into 10 equally sized bins according to their degree and drawing random genes from these bins based on the degree distribution of the actual gene sets. Statistical significance of observed overlaps was assessed by computing empirical *P* values, defined as the proportion of random overlaps more extreme than the observed overlap. Resulting *P* values were corrected for multiple testing using the Benjamini–Hochberg procedure as implemented in the statsmodels package^102^ (v0.14.3). Coefficients different from zero were assessed using two-sided *t*-tests with Benjamini–Hochberg correction. Coefficients with adjusted *P* < 0.1 were considered significant and indicated with a black outline in the plot.

### Bulk RNA-seq

#### Sample preparation

Brain tissue from *Bckdk*^*−/−*^, *Cul3*^*+/−*^, *HnrnpU*^*+/−*^ and C57BL/6J colony-matched controls (*n* = 3 per sex + genotype) across time points (E14.5, P4 and P14), as well as from E16.5 *HnrnpU* and WT littermate mice (*n* = 3 per sex + genotype) were used for the bulk RNA-seq experiment. Animals coming from at least three separate litters were collected to avoid potential litter-specific biases. Timed-pregnant females were decapitated, and embryos were rapidly dissected on ice. Cortical tissue was dissected in ice-cold PBS, for embryonic tissue, the meninges were removed, and the tissue (whole brain for E16.5 *HnrnpU* animals, forebrain for mutants linked with ASD at E14.5, P4 and P14) was snap-frozen in liquid nitrogen and stored at −80 °C. RNA extraction for the E16.5 *HnrnpU* dataset was performed using TRIzol–chloroform extractions as described in the Methods section for RNA isolation. For the dataset using multiple mutants linked with ASD at E14.5, P4 and P14, the RNAeasy Mini RNA isolation kit (74104, Qiagen) was used according to the manufacturer’s protocol. One microgram of RNA obtained was used for cDNA library preparation using the NEBNext Ultra II Directional RNA Library Prep kit (E7765, NEB) with unique dual indexing primers (NEBNext Multiplex Oligos for Illumina, E6440S, NEB) and poly(A) selection with the NEBNext Poly(A) mRNA Magnetic Isolation Module (E7490, NEB), according to the manufacturer’s protocol. RNA quality, library quality and size were assessed with the RNA 6000 Nano Kit (5067-1511, Agilent) and High Sensitivity DNA Analysis Kit (5067-4626, Agilent) and the Bioanalyzer 2100. Libraries were sequenced on the Illumina NextSeq 2000 and NovaSeq X platforms by the Vienna Bio Center NGS facility.

#### Pre-processing and analysis of RNA-seq

For the analysis of E16.5 *HnrnpU* mutant and WT animals, demultiplexed FASTQ files were filtered and trimmed using Cutadapt^103^ (v4.1) and aligned to the mm10 reference genome using STAR^104^. Similarly, this method was used to obtain read counts. All further downstream analyses were performed in R. The same pipeline used for pseudobulk DGE analysis of the snRNA dataset as described above was used. However, the pseudobulking step was omitted, and only the mutant genotype was compared with WT littermate mice. One sample (HET genotype, female) was removed due to an increased number (approximately 10%) of duplicated sequences. For the analysis of mutants linked with ASD (*Bckdk*^*−/−*^, *Cul3*^*+/−*^ and *HnrnpU*^*+/−*^) and C57BL/6J colony-matched controls, data were analysed using R (v4.5.0). Demultiplexed FASTQ files were filtered and trimmed using Cutadapt^103^ (v4.1). Samples were aligned to the GRCm39 reference genome using STAR (v2.7.11b), and gene-level counts were obtained using FeatureCounts (v2.1.1)^105^. Mutant samples were compared with WT C57BL/6J colony-matched controls. Unwanted variation was corrected using the RUVSeq package (v1.42.0)^106^. To define empirical control genes, an initial DESeq2 analysis was performed, and all genes were ranked by *P* value. The top 5,000 DEGs were excluded, and the remaining genes were used as empirical controls for RUV (remove unwanted variation) correction. The value of *k* was selected as the lowest number that effectively reduced unwanted variation, based on visual inspection of correction effects in the PCA space, following the recommendations of Risso et al.^106^.

### RNA isolation and quantitative real-time PCR

Brain tissue from male and female *Ash1l*^*+/−*^, *HnrnpU*^*+/−*^, *Kdm6b*^*+/−*^, *Kmt5b*^*+/−*^, *Trip12*^*+/−*^, *Usp7*^*+/−*^ and *Wac*^*+/−*^ and their WT littermates were used to validate the expression of targeted genes. For RNA extraction of cortical samples, 700 µl TRIzol (Invitrogen) and 150 µl chloroform (Sigma) were used for homogenization, followed by centrifugation at 12,000*g* for 15 min at 4 °C. For cerebellar samples, 600 µl TRIzol and 130 µl chloroform were used.

The upper aqueous phase was transferred to a new tube, and 1.5 volumes of 100% ethanol (EtOH) were added. RNA was extracted using Zymo-Spin IC columns (Zymo Research). The aqueous phase–ethanol mixture was loaded onto the column and washed with 400 µl of 70% EtOH following treatment with RQ1 DNAseI (Promega; 5 µl + 5 µl reaction buffer + 40 µl 70% EtOH) for 15 min at room temperature. After two washes with 70% EtOH, the sample was eluted from the column with DEPC-treated H_2_O. RNA concentration was measured using the NanoDrop spectrophotometer (Thermo Scientific). Of RNA, 1 µg was used for cDNA preparation with the RevertAid First Strand cDNA Synthesis Kit (Thermo Fisher). cDNA was diluted 1:5 and used for quantitative PCR analysis with Lightcycler 480 Sybr green master mix (Roche) on a Real-Time PCR Roche Lightcycler 480 machine. Samples were run in triplicates for target genes, and a list of all oligonucleotides used is provided in Supplementary Table 1. ΔCq expression levels (relative mRNA) were calculated upon normalization to housekeeper genes and plotted.

### Cytometry, imaging and analysis

#### Sample preparation and imaging of EdU-injected and CUBIC-cleared brains

Timed-pregnant female mice were injected with 0.05 mg g^−1^ of 5-ethynyl-2′-deoxyuridine (EdU) at E14.5 and euthanized 6 h later; embryos were decapitated, rapidly dissected on ice and immersion fixed in 4% paraformaldehyde overnight at 4 °C, followed by a washing step in 1X PBS. Cells in S-phase that incorporated EdU were detected using click chemistry (BCK-EdU555IM100+IV-M, Baseclick). Embryonic heads were cleared using the CUBIC clearing protocol^107^ with adaptations coupled with EdU staining. In brief, fixed, washed samples were incubated for 4 h at 37 °C on a rotating shaker in CUBIC-L/R1a in mH_2_O. Samples were incubated in CUBIC-L/R1a solution for 48 h at 37 °C on a rotating shaker with a change of solution after 24 h. Afterwards, samples were washed with PBS and incubated overnight at 37 °C with PBS–0.2% Triton X-100–20% DMSO–0.3 M glycine. Before incubating the samples with the EdU reaction solution according to the manufacturer’s protocol overnight, samples were incubated for 2 h in PBS–0.2% Triton X-100 and briefly washed twice with PBS. After incubating with the EdU reaction, samples were again washed twice with PBS and 2 h in PBS–0.2% Triton X-100. To match the refractive index, samples were stepwise incubated for 2 h at 37 °C in ascending concentrations of CUBIC-R + (N) in mH_2_O. In the case that embryonic heads were not fully transparent, they were incubated overnight in CUBIC-L/R1a at 37 °C on a rotating shaker, followed by stepwise refractive index (RI) matching. Cleared heads were imaged in oil (Gelest #PDM-7040, RI of 1.556; and J62592.AP, Thermo Fisher, RI of 1.467). Both oils were mixed to match the refractive index (RI ~ 1.52) of the cleared heads. Imaging was performed on a Zeiss Lightsheet 7 microscope equipped with dual-side illumination and two camera detection (PCO edge 4.2 sCMOS) using ZEN Black imaging software (v3.10) using 5×/0.1foc illumination and 5×/0.16 EC-Plan Neofluar detection objectives with the following settings: filter set: band pass 505-530/LP560/LP585, Zoom 1.4, lightsheet thickness of 7.76 µm, scaling *xyz* of 0.67 µm × 0.67 µm × 1.5 µm. Imaging was performed using single-side illumination to achieve optimal sample illumination and to prevent potential duplication of nuclei in the images. Whenever possible, both the left and right brain hemispheres were imaged, and damaged or incompletely cleared samples were excluded from the analysis. Images of EdU-stained, cleared embryo heads were analysed automatically using Zeiss Arivis Pro software (v4.2.0). A semi-automated analysis pipeline was optimized to count EdU-positive cells within one cortical hemisphere, defined as a region of interest, using the Blob Finder function implemented in Zeiss Arivis Pro (v4.2.0).

#### Sample preparation and analysis of EdU-injected brains by cytometry

Timed-pregnant female mice were injected with 0.05 mg g^−1^ EdU at E14.5 and euthanized 2 h later; embryos were rapidly dissected on ice. Cortical tissue was isolated in ice-cold HBSS (14175053, Thermo Fisher), meninges were removed and forebrain structures were dissociated using an established papain-DNAse protocol^108^. Following dissociation, S-phase cells that incorporated EdU were detected by click chemistry (BCK-EdU488FC50+IV-S, Baseclick). In brief, washed samples were fixed for 15 min at room temperature and permeabilized using the supplied 1X saponin-based permeabilization and wash buffer. Two washes (1% BSA in PBS, 300*g* for 10 min each) were performed between fixation and permeabilization. Following fixation and permeabilization, the click reaction was prepared according to the manufacturer’s protocol, and cells were incubated for 30 min at room temperature, protected from light. Samples were washed twice with supplied 1X saponin-based permeabilization and wash buffer and centrifuged at 300*g* for 10 min per wash. A control sample was processed identically up to the permeabilization step and used as negative control for cytometry analysis. Samples were analysed on a Beckman Coulter Cytoflex LX. Cell populations were gated based on forward scatter versus side scatter, and doublets were excluded via forward scatter area versus height gating. Fluorescence of EdU in gated single cells was measured in the B525-FITC-A channel (excitation of 488 nm and emission of 525/40 band pass. Total events (*n* = 50,000) were acquired per sample, and gating and analysis were performed using Beckman Coulter’s CytExpert software (v2.7).

#### RNAscope assay

Spatial gene expression analysis was performed using the RNAscope94 Multiplex Fluorescent v2 Assay (323110, ACD) kit including specific probes targeting *Pdgfra* and *Slc1a3* mRNA (480661 and 430781-C3, ACD). For sample preparation, P4 mice were decapitated, and the brain was dissected rapidly on ice. The cerebellum was removed, and the brain was embedded in pre-cooled Tissue-Tek OCT Compound (Sakura Finetek) and stored at −80 °C until further use. Tissue was sliced at 20 μm at a CryoStar NX70 cryostat (Thermo Scientific) and directly mounted on SuperFrost Plus Adhesion slides (Epredia). Sections were stored at −80 °C until use. The assay was performed according to the instructions provided by the RNAscope Multiplex Fluorescent v2 Assay kit. In brief, sections were fixed in 4 °C cold 4% paraformaldehyde and further pre-treated using a H_2_O_2_ and protease digestion. After pre-treatment, sections were incubated with the probes targeting the mRNAs of interest. Probes were further hybridized to a cascade of signal amplification molecules, followed by a hybridization with TSA vivid fluorophore 520 for *Pdgfra* and TSA vivid fluorophore 570 for *Slc1a3* (323271 and 323272, ACD). After the hybridization steps, sections were stained with a nuclear counterstain and mounted using ProLong Gold Antifade Mountant (P36930, Invitrogen). Images were acquired using a LSM900 inverted confocal microscope using 10× objective and analysed in ImageJ. To assess the number of astrocytes and OPCs, *Slc1a3*^*+*^ and *Pdgfra*^*+*^ cells were manually quantified in blinded images of coronal brain sections of similar size in at least three images per mouse.

#### Electrophysiology

Brain slices were obtained from P7 *Bckdk*^*−/−*^, *HnrnpU*^*+/−*^, *Trip12*^*+/−*^, *Usp7*^*+/−*^, littermate and colony-matched C57BL/6J control mice. Acute coronal slices (300 µm) were prepared from the primary somatosensory cortex. Animals were decapitated and whole brains were rapidly removed from the skull and sectioned using a VT 1200S vibratome (Leica Microsystems). Slices were then allowed to recover in in regular artificial cerebrospinal fluid in 1 mM Ca^2+^ at 35 °C for 10 min, followed by a cool-down to room temperature for at least 20 min in artificial cerebrospinal fluid, containing: 118 mM NaCl, 2.5 mM KCl, 1.5 mM MgSO_4_ × 7H_2_O, 1.25 mM NaH_2_PO_4_, 2 mM CaCl_2_, 20 mM sucrose, 3 mM myo-inositol, 10 mM glucose and 26 mM NaHCO_3_ (320 mOsm l^−1^, pH 7.35–7.40).

Patch pipettes (3–5 MΩ; Sutter Instrument) were pulled on a P-1000 puller (Sutter Instrument) and filled with the intracellular recording solution, containing: 120 mM K-gluconate, 20 mM KCl, 2 mM MgCl_2_, 10 mM HEPES, 0.5 mM EGTA, 2 mM MgATP, 0.2 mM NaGTP, 23.4 mM sucrose and 0.3% w/v biocyin; adjusted to a pH of 7.35, and osmolarity adjusted to 302 mOsm l^−1^.

Excitability (current versus action potential frequency) was measured in current clamp mode by applying current steps from −10 to +50 pA in 5-pA increments (1-s duration and 8-s inter-sweep interval). Input resistance (Rin) was calculated as the linear regression of the current–voltage relationship from −10 to 0 pA. Membrane time constant tau was measured by an exponential fit of the initial voltage change in response to a −10-pA current injection. Membrane capacitance was calculated by dividing tau by Rin. Resting membrane potential was measured as the average voltage at the 0-pA current injection sweep. Action potential properties were measured in the first evoked action potential, with threshold potential measured at the onset of the steep rising phase. Action potential amplitude was measured as the difference between threshold and peak potential. Action potential full-width at half-maximum was measured as the time between rising and falling phase at half of the maximal amplitude. Action potential afterhyperpolarization was measured as the voltage 50 ms after the peak relative to the threshold potential. mEPSCs and mIPSCs were recorded for 3–5 min in voltage clamp mode at a holding potential of −60 mV and in the presence of 1 µM tetrodotoxin + 10 µM bicuculline methiodide (mEPSCs) or 1 µM tetrodotoxin + 10 µM DNQX + 50 µM D-AP5 (for mIPSCs). Access resistance (Ra) was monitored throughout all recordings, and recordings with Ra exceeding 20 MΩ were excluded. Miniature neurotransmission was manually analysed using Mini Analysis Program (Synaptosoft, v6.0.7). Passive and active membrane properties were analysed using Clampfit (v11.3.0.02). All recordings and analyses were performed with the experimenter blind to the animal experimental condition.

#### Minimal conductance-based model

To model the electrophysiological properties of WT neurons, we utilized the biophysical model of a layer II/III pyramidal neuron from the mouse visual cortex provided by the Allen Brain Institute (https://modeldb.science/184162). Membrane properties and morphology were modified to match the developmental stage of P7 and electrophysiological characteristics (Rin and membrane time constant) observed in our data. The soma and dendrites were scaled to 70% of their original radius and length, and the axon was reduced to 50% of its original dimensions. To match the observed frequency–current relationship, the membrane capacitance was set to 0.38× and the passive membrane conductance to 0.5× of the original values in their respective compartments. Reversal potentials were set to −75 mV for passive current, +30 mV for Na^+^ and −110 mV for K^+^ ions. Simulations were performed in Python 3 using the NEURON simulator^109^. Following the described parameter adjustments, the model exhibited a net input resistance of approximately 988 MΩ, a membrane time constant of 63 ms and a resting membrane potential of −61 mV, approximately matching the mean passive properties of a WT control neuron.

The model includes multiple ion channel types: persistent potassium (Kp), delayed rectifier potassium (Kt), A-type potassium (Kv3.1), Ca^2+^-activated potassium (SK), non-inactivating potassium (M-channel), persistent sodium, transient sodium, hyperpolarization-activated cyclic nucleotide-gated (Ih), Im channels, high-voltage-activated calcium and low-voltage-activated calcium channels.

### Behavioural analysis of *Bckdk*-mutant and *Kmt5b*-mutant animals

#### Gait recordings

*Bckdk*-mutant and WT littermate animals (P13–15) were acclimated to the testing room 1 h before behavioural recording and were not handled before testing due to their young age. Animals were led to explore an empty open-field arena (50 × 50 × 40 cm) constructed from IR-transparent acrylic covered with a lid, creating a dark environment during testing. Animals were recorded from below using an IR-camera (Basler acA3088-57µm) ensuring continuous visibility of the paw of the animals. Video acquisition was performed with OBS Studio (v31.0.1). Each recording lasted a minimum of 15 min to capture a sufficient number of stride cycles. The experimenter was blind to the genotype during recordings. Pairs of control-mutant littermates were randomly tested in the morning and in the afternoon to control for circadian rhythm.

#### Analysis of mouse pose estimation and gait

Body parts of mice were tracked using SLEAP^110^ (v1.4.1) by defining a SLEAP skeleton containing 14 body parts. Four are at the head of the mouse (nose, ear left, ear right and neck base), the four paws (forepaw left, forepaw right, hindpaw left and hindpaw right), at the centre and sides of the mouse body (spine centre, left side, right side) and three parts for the tail (tail base, tail middle and tail tip).

After annotating 364 frames of a single mouse across WT and mutant conditions, we trained both a centroid and a centred instance model using the top-down pipeline in SLEAP. In the centroid model, which detects the central body region, we used an input scaling of 0.5, an output stride of 2, and defined spine centre as the anchor point with σ = 2.5 px. All other network parameters were kept at their default values. The model was trained for 128 epochs with early stopping enabled (plateau patience = 32 epochs).

The centred instance model, which detects all body parts within a bounding box centred on the anchor point predicted by the centroid model, was configured with an input scaling of 1.0, output stride of 2, maximum stride of 32, number of filters of 32 and the filter rate of 2. We used a σ = 1.75 for the body parts. This network was trained for 500 epochs allowing for early stopping (plateau patience = 84 epochs).

Both models used a UNet backbone and were trained with an Adam optimizer (initial learning rate = 0.0001). Data augmentation included random rotations, horizontal flips and random size variations between a scale of 0.95 and 1.05. The validation dataset comprised 10% of the total annotated frames. Training was performed on a NVIDIA RTX 4080 SUPER GPU with 16 GB of VRAM.

The centroid model achieved a detection precision and recall of 1 on the validation set. The centred instance model reached a body-part recall of 0.992 and precision of 0.996, with an average pixel error of 2.59 px. To track the detected mouse poses, the default centroid tracker in SLEAP using the Hungarian method for instance matching in a frame window of size 5 was used.

To analyse mouse gait, we implemented mouse gait and posture metrics as previously described^111^. To extract strides, we first egocentrically aligned each mouse pose by centring the mouse to its central part (that is, spine centre) and rotating the coordinate system such that the neck base pointed upwards along the *y* axis. In this egocentrically aligned coordinate system, local maxima of the *y* location of paws (for example, forepaw left) were identified as the time point of foot-strike events, marking the start of the stance phase. Similarly, local minima determine the toe-off event, marking the beginning of the swing phase.

Before detecting extrema, paw trajectories were smoothed with a one-dimensional Gaussian filter (*σ* = 3 frames, corresponding to 0.05 s). Maxima and minima were then extracted using the scipy.signal.find_peaks function, requiring a minimum peak prominence of 11 px (0.6 cm).

Strides extracted from one paw part were validated by the corresponding opposite paw. A stride was considered valid if, during its stance phase, the opposite paw exhibited a foot-strike event. Both forepaw and hindpaw strides were validated and included in downstream stride analyses.

To focus on periods of active locomotion, valid strides were filtered to include only those occurring during forwards movement. Forwards motion was quantified by projecting the displacement vector (instantaneous speed) of the mouse onto its body axis (defined by the unit vector from spine centre to neck base). This resulting forwards speed was smoothed using a Gaussian kernel (σ = 60 frames; 1 s) and thresholded with 0.5 cm s^−1^ to define periods of forwards movement. Only strides occurring during forwards movement were used for gait metric computation. All raw stride, step and speed metrics were normalized to the body length of the mouse, defined by the distance from tail base to nose. All resulting stride metrics were averaged per movie.

Stride extraction and metric computation were implemented using the TadPose library^112^ (v0.4.0), a Python library for the analysis of SLEAP pose-estimation data. Mouse forwards movement was calculated in custom Python Jupyter notebook using the scipy library (v0.15.3). Left and right forepaws and hindpaws were analysed separately but averaged to obtain overall gait metrics distinguishing between forepaw and hindpaw movements.

### Statistics

Information on the statistical analyses is provided in the corresponding Methods sections. Blinding and randomization were not applied unless otherwise specified. Each *n* value refers to the number of biological replicates (that is, animals or cells) used per condition.

### Reporting summary

Further information on research design is available in the Nature Portfolio Reporting Summary linked to this article.

## Online content

Any methods, additional references, Nature Portfolio reporting summaries, source data, extended data, supplementary information, acknowledgements, peer review information; details of author contributions and competing interests; and statements of data and code availability are available at 10.1038/s41586-026-10679-1.

## Supplementary information

Supplementary Information Supplementary Notes, Supplementary Discussion, Supplementary Figs. 1–3 and descriptions for Supplementary Data Tables 1–13.

Reporting Summary

Supplementary Data Supplementary Data Tables 1–13.

Supplementary Video 1 Cleared embryo head combined with EdU-labelling. EdU-labelling at E14.5 was combined with CUBIC clearing and imaged on a Zeiss Lightsheet microscope.

Supplementary Video 2 Example video of *Bckdk*^*+/+*^ animal during gait recordings. Recording of a *Bckdk*^*+/+*^ animal used for gait analysis, with stride renderings overlaid on the video frame. Video recorded at 60 fps.

Supplementary Video 3 Example video of *Bckdk*^*−*^^*/*^^*−*^ animal during gait recordings. Recording of a *Bckdk*^*−/−*^ animal used for gait analysis, with stride renderings overlaid on the video frame. Video recorded at 60 fps.

Peer Review File

## Source data

Source Data Fig. 3, 5, 6 and Extended Data Fig. 2, 3, 9

## Data Availability

Single-nucleus multiomics data are available from the Gene Expression Omnibus (GSE328363). The mm10 reference genome was used for the alignment (refdata-cellranger-arc-mm10-2020-A-2.0.0, obtained from https://cf.10xgenomics.com/supp/cell-arc/refdata-cellranger-arc-mm10-2020-A-2.0.0.tar.gz). Single-cell data can be accessed and visualized through a CELLxGENE database (https://adameykolab.hifo.meduniwien.ac.at/cellxgene_public/filecrawl/.2026_Nature_Schwarz). Source data are provided with this paper.

## Code availability

Scripts and analyses that support the main findings of this study are accessible in a GitHub repository (https://git.ista.ac.at/research-sofware/mouseome).
