# Supplementary material for: Cortical development dynamics across autism spectrum disorder mouse models
Source: Nature. 2026 Jun 17;656(8126):159–72. doi: 10.1038/s41586-026-10679-1 (PMC13441911; doi:10.1038/s41586-026-10679-1)
Supplement: Supplementary file 1 [file 41586_2026_10679_MOESM1_ESM.pdf]

nature portfolio

https://doi.org/10.1038/s41586-026-10679-1

Supplementary information

Cortical development dynamics across autism spectrum disorder mouse models

In the format provided by the authors and unedited

Supplementary Information

Cortical development dynamics across autism spectrum disorder mouse models

Schwarz, Lena A. et al.

Supplementary Notes 2
Supplementary Discussion 3
Supplementary Figures 9
Supplementary Legends 13

Supplementary Notes

Gene selection

We analyzed constitutive mutants for *Ash1l*, *Bckdk*, *Cul3*, *HnrnpU*, *Kdm6b*, *Kmt5b*, *Ptchd1*, *Setd5*, *Trip12*, *Usp7,* or *Wac,* together with colony-matched wild-type controls (Supplementary Fig. 1a). Models were selected to represent diverse functional pathways, inheritance modalities, and ubiquitous expression profiles (Supplementary Fig. 1b,c). All selected genes are high-confidence ASD-risk genes (SFARI Gene Score 1, V2023 Q4), except *Usp7* (Score 2). Except for *Bckdk* and *Ptchd1*, we modeled *de novo*, heterozygous, loss-of-function mutations as reported in patients[1–9]. As observed in ASD patients, we studied homozygous mutants for *Bckdk* [10,11], and hemizygous males for the X-linked *Ptchd1*[12,13].

Sex-stratified and combined analyses validate sex-specific differences in DEGs

Because variations in Cohen's d values may reflect differences in expression levels and/or variability in mutant and control samples, we tested whether slight sex differences among controls influenced the results. We therefore pooled female and male controls and compared this combined group to female or male mutants. The pattern persisted, with several genotypes showing more DEGs in females than males (Supplementary Fig. 2a). We independently validated these results by bulk RNAseq of *HnrnpU* E16.5 mutant and wild-type littermate brains. Females again showed higher DEG numbers than males (Supplementary Fig. 2b) with the directionality of these DEGs preserved (Supplementary Fig. 2c-c'').

snRNAseq of male cerebellar samples

We selected a single sex (male) and time point (P4) (Fig. 6, Supplementary Fig. 3), a stage capturing diverse cerebellar cell types within a single sample[14].

We identified 16 primary cell types (Fig. 6a), with balanced distribution of samples and genotypes across all clusters (Supplementary Fig. 3b,c). As expected for this developmental stage[14], we primarily identified Granule cells (*Reln, Rbfox3)* and a small number of Purkinje cells (*Calb1*). We could differentiate between molecular cell layer interneurons (MLI; *Grm8, Nxph1*), Purkinje cell layer interneurons (PLI; *Klhl1, Chrm2*), and unipolar brush cells (UBC; *Lmx1a*). Several glial cell types were detected, including Astrocytes (*Apoe*), Bergmann Glia (*Zeb2*), Microglia (*Cxcr3*), and OPCs *(Pdgfra, Mag;* Supplementary Fig. 3d).

Supplementary Discussion

ASD comprises a heterogeneous group of genetic conditions. Key questions are how all these different disorders develop, the extent of shared pathophysiology, and whether common therapeutic time windows and targets exist. By profiling monogenic ASD models across development, we uncovered dynamic changes in the brain cellular and molecular landscapes, highlighting the complexity of ASD trajectories.

ASD mutants converged on altered radial glial lineage progression, likely reflecting developmental delay rather than a specific alteration in neural lineage specification, as changes emerged early in development and resolved as the brain matures. Consistently, we observed no differences in neuronal cell proportions at P14. These observations align with the literature. Mutations in *ASH1L, CUL3, HNRNPU, SETD5, TRIP12, USP7,* or *WAC* have not been linked to altered brain size in patients. In contrast, patients and mice with *BCKDK* mutations develop postnatal microcephaly[10] (and unpublished data), whereas ~26% of patients with *KDM6B* mutations present with macrocephaly[15]. About 60% of patients with *KMT5B* mutations show macrocephaly; however, this phenotype is not clearly linked to altered proliferation, as the enlargement could reflect a cortical or subcortical phenotype[16]. A lack of persistent neuronal number changes beyond early development also aligns with human postmortem ASD studies[17]. Together, these findings suggest that neither monogenic nor idiopathic ASD is associated with differences in neuronal cell numbers, except for transient developmental effects. Such transient shifts raise the question of whether early, minor cell count variations could lead to lasting connectivity alterations.

In contrast to human data[17], we observed no changes in microglial numbers, suggesting that microglia activation might be an indirect effect emerging later in life. However, postnatal analysis in *Cul3*, *Setd5*, and *Ptchd1* mutants confirmed no significant difference in overall microglial counts (data not shown).

Although cell numbers normalize over time, P4 exhibited the most pronounced molecular alterations. This is intriguing since functional genomic studies hypothesized that ASDs converge on excitatory neuron perturbation at human mid-fetal developmental stages, corresponding to early postnatal stages in mice[18–20]. This pattern is consistent with regulatory priming earlier in development and with peak chromatin accessibility changes at E14.5.

We also found variations across genotypes. *HnrnpU* and *Kdm6b* mutants showed many changes at E14.5 and P4 but not P14, whereas *Bckdk* and *Trip12* mutations

affected P4 and P14. Other mutants showed minimal effects throughout. Given the temporal ubiquitous expression of the ASD genes we have modeled, these results suggest that the developmental context may shape phenotypic outcomes. Transient and milder differences may also be exacerbated during particular behavioral tasks.

The same mutation can affect different biological processes at distinct developmental stages, indicating that ASD mutation effects evolve over time. This temporal variability may explain the challenges in identifying reliable biomarkers and achieving an early diagnosis.

Although DEG profiles differed between mutants, we identified shared DEGs and convergent biological processes, indicating that, despite genetic heterogeneity, ASD mutations result in similar cell states. Our data suggest that convergence may be driven by developmental stage rather than mutation-specific programs. Additionally, convergence on synaptic and ion-channel functions may represent homeostatic compensation for early developmental delays. Nevertheless, the enrichment for ion channel-related genes among DEGs aligns with prior evidence linking ASD to excitation/inhibition imbalances[21]. Although we observed no shifts in the excitatory-to-inhibitory quantal release ratio, electrophysiology analysis indicates altered neuronal excitability that scaled with corresponding transcriptional profiles. A conductance-based computational model, based on our gene expression results, reproduced key features of the recorded responses. More advanced computational models could therefore bridge the gap between molecular changes and phenotypes and predict excitability states from ‘omics’ datasets.

Parallel analysis of multiple ASD models also underscored differences. Mutations in genes with similar molecular functions, such as histone modifiers (e.g., *Ash1l*, *Kdm6b*, and *Kmt5b*) or ubiquitin-related genes (e.g., *Cul3*, *Trip12*, and *Usp7*), did not show greater molecular or phenotypic similarity to one another than to mutants in other functional groups. Furthermore, a large fraction (on average 50%) of DEGs was restricted to a particular mutant. Many of these changes were consistent across developmental stages, suggesting potential genotype-specific biomarkers and likely reflecting direct effects of the causal mutations, whereas shared changes may represent downstream effects. These findings suggest that multiple pathways result in similar cellular states. For example, *CamKIIa* was one of the most commonly regulated DEG. *HnrnpU* is known to bind to *CamKIIa* mRNA and transport it at the synapse, thus potentially directly regulating its levels[22], whereas other ASD genes may regulate this

transcript indirectly. Whether commonly affected processes reflect compensatory resilience pathways or core pathogenic mechanisms remains to be discovered.

To expand our analysis, we investigated the cerebellum, given its connection to the motor and cognitive challenges observed in ASDs. At the analyzed time point, most mutations did not alter cerebellar cell numbers or gene expression, although samples were obtained from the same animals as cortical samples. Although this could be a limitation of the chosen time point, human data support a weaker impact of ASD mutations on the cerebellum than on the cortex[23]. Where changes were noted, as in *Bckdk* mutants, cerebellar transcriptional alterations largely matched those in the cortex, suggesting shared affected pathways. These alterations in *Bckdk* mutants were accompanied by motor deficits. Given the high prevalence of motor impairments in ASD, it will be important to determine whether ASD-associated mutations can lead to similar deficits at later stages and to dissect the contribution of other motor-related structures, such as the striatum. Addressing this will require longitudinal, cell-type-resolved analyses across brain regions, combined with circuit-level perturbation and rescue experiments.

Our experimental design also enabled detection of sex differences. Although the ASD forms studied arise from rare mutations identified in both sexes, some mutations produced larger effect sizes in females. A recent large-scale sequencing study of adult brain samples from patients with schizophrenia, bipolar disorder, and ASD reported similar sex-biased effects[24]. Whether the larger effect size in females is a result of stronger responses or sex-specific resilience remains unclear. However, it is interesting that several of the fsDEGs were shared across mutants and included many transcripts related to synaptic functions and ion channels. Interestingly, some of the shared fsDEGs are X-linked and have been shown to escape X-inactivation[25–28], thus potentially capable of activating female-specific mechanisms. For example, *Nkap* was upregulated in excitatory neurons in females at P4 in *HnrnpU* and *Trip12,* two of the mutants that showed the highest female effect sizes. *Nkap* can escape X-inactivation and is a known activator of NF-κB and a repressor of Notch[29], critical for brain development and functioning[30–34]. Mutations in *Nkap* itself have been identified as a cause of intellectual disability, affecting only males[35]. Furthermore, ATAC-seq analysis indicated that DARs were enriched for the TF Sp1. Sp1 is known to bind to estrogen receptors[36] and has recently been linked to sex-biased gene expression in primate brains[37]. Finally, our analysis suggests that female-male differences may be partially

transient rather than long-lasting along the disorder trajectory. This argues for studying developmental trajectories separately in male and female patients, as critical windows and, thus, optimal treatment timing may differ.

Limitations

While this study presents a step forward in understanding the biology, convergence, and divergence of genetically distinct forms of ASD, it also has limitations. First, droplet-based single-cell sequencing has a relatively low sensitivity, potentially missing lowly expressed transcripts. Therefore, the true number of DEGs may be underestimated. Although this likely does not impact the overall conclusions, some of the biological effects or gene sets might be missing. Second, while we offer a view of the temporal evolution of the mutants, gaps between time points remain. Mutants showing modest effects here might show greater changes at other time points. Third, uniform shifts in absolute cell numbers across all types would not be detected by snRNA-seq, which only provides information on relative cell-type proportions. Fourth, studies of this scale are inherently resource-intensive and require cost-balancing measures, including relatively small sample sizes and shared controls. This design may reduce statistical power to detect subtle effects and could contribute to modest effect sizes in some comparisons. In addition, variability within a small control group may increase the apparent similarity across mutants, potentially inflating signals of convergence. Finally, our analysis is conducted in naïve animals; state-dependent accessibility effects or gene expression changes may be context-dependent. To address this, future work should examine selected mutants during particular behavioral tasks or stimuli, potentially revealing additional differences also later in life.

References

1. De Rubeis, S. *et al.* Synaptic, transcriptional and chromatin genes disrupted in autism. *Nature* **515**, 209–215 (2014).
2. Shen, W., Krautscheid, P., Rutz, A. M., Bayrak-Toydemir, P. & Dugan, S. L. *De novo* loss-of-function variants of *ASH1L* are associated with an emergent neurodevelopmental disorder. *Eur. J. Med. Genet.* **62**, 55–60 (2019).
3. Stolerman, E. S. *et al.* Genetic variants in the KDM6B gene are associated with neurodevelopmental delays and dysmorphic features. *Am. J. Med. Genet. A.* **179**, 1276–1286 (2019).
4. Faundes, V. *et al.* Histone Lysine Methylases and Demethylases in the Landscape of Human Developmental Disorders. https://doi.org/10.1016/j.ajhg.2017.11.013 doi:10.1016/j.ajhg.2017.11.013.
5. Hao, Y.-H. *et al.* USP7 acts as a molecular rheostat to promote WASH-dependent endosomal protein recycling and is mutated in a human neurodevelopmental disorder. *Mol. Cell* https://doi.org/10.1016/j.molcel.2015.07.033 (2015) doi:10.1016/j.molcel.2015.07.033.
6. Fountain, M. D. *et al.* Pathogenic variants in USP7 cause a neurodevelopmental disorder with speech delays, altered behavior, and neurologic anomalies. https://www.gimjournal.org/article/S1098-3600(21)01632-4/fulltext.
7. Bramswig, N. C. *et al.* Identification of new TRIP12 variants and detailed clinical evaluation of individuals with non-syndromic intellectual disability with or without autism. *Hum. Genet.* **136**, 179–192 (2017).
8. Zhang, J. *et al.* Haploinsufficiency of the E3 ubiquitin-protein ligase gene TRIP12 causes intellectual disability with or without autism spectrum disorders, speech delay, and dysmorphic features. *Hum. Genet.* **136**, 377–386 (2017).
9. DeSanto, C. *et al.* WAC loss-of-function mutations cause a recognisable syndrome characterised by dysmorphic features, developmental delay and hypotonia and recapitulate 10p11.23 microdeletion syndrome. https://doi.org/10.1136/jmedgenet-2015-103069 (2015) doi:10.1136/jmedgenet-2015-103069.
10. Novarino, G. *et al.* Mutations in BCKD-kinase Lead to a Potentially Treatable Form of Autism with Epilepsy. *Science* **338**, 394–397 (2012).
11. García-Cazorla, A. *et al.* Two Novel Mutations in the (Branched-Chain Keto-Acid Dehydrogenase Kinase) Gene Are Responsible for a Neurobehavioral Deficit in Two Pediatric Unrelated Patients. *Hum. Mutat.* **35**, 470–477 (2014).
12. Noor, A. *et al.* Disruption at the PTCHD1 Locus on Xp22.11 in Autism Spectrum Disorder and Intellectual Disability. *Sci. Transl. Med.* **2**, 49ra68-49ra68 (2010).
13. Chaudhry, A. *et al.* Phenotypic spectrum associated with PTCHD1 deletions and truncating mutations includes intellectual disability and autism spectrum disorder. *Clin. Genet.* **88**, 224–233 (2015).
14. Sarropoulos, I. *et al.* Developmental and evolutionary dynamics of cis-regulatory elements in mouse cerebellar cells. *Science* **373**, eabg4696 (2021).
15. Rots, D. *et al.* The clinical and molecular spectrum of the *KDM6B*-related neurodevelopmental disorder. *Am. J. Hum. Genet.* **110**, 963–978 (2023).
16. Sheppard, S. E. *et al.* Mechanism of KMT5B haploinsufficiency in neurodevelopment in humans and mice. *Sci. Adv.* **9**, eade1463 (2023).
17. Wamsley, B. *et al.* Molecular cascades and cell type–specific signatures in ASD revealed by single-cell genomics. *Science* **384**, eadh2602 (2024).
18. Willsey, A. J. *et al.* Coexpression Networks Implicate Human Midfetal Deep Cortical Projection Neurons in the Pathogenesis of Autism. https://doi.org/10.1016/j.cell.2013.10.020 doi:10.1016/j.cell.2013.10.020.

19. Parikshak, N. N. *et al.* Integrative functional genomic analyses implicate specific molecular pathways and circuits in autism. *Cell* **155**, 1008–1021 (2014).
20. Geschwind, D. H. & State, M. W. Gene hunting in autism spectrum disorder: on the path to precision medicine. *Lancet Neurol.* **14**, 1109–1120 (2015).
21. Rubenstein, J. L. R. & Merzenich, M. M. Model of autism: increased ratio of excitation/inhibition in key neural systems. *Genes Brain Behav.* **2**, 255–267 (2003).
22. Kanai, Y., Dohmae, N. & Hirokawa, N. Kinesin Transports RNA: Isolation and Characterization of an RNA-Transporting Granule. *Neuron* **43**, 513–525 (2004).
23. Parikshak, N. N. *et al.* Genome-wide changes in lncRNA, splicing, and regional gene expression patterns in autism. *Nature* **540**, 423–427 (2016).
24. Xia, Y. *et al.* Transcriptomic sex differences in postmortem brain samples from patients with psychiatric disorders. *Sci. Transl. Med.* **16**, eadh9974 (2024).
25. Sauteraud, R. *et al.* Inferring genes that escape X-Chromosome inactivation reveals important contribution of variable escape genes to sex-biased diseases. *Genome Res.* **31**, 1629–1637 (2021).
26. Wainer Katsir, K. & Linial, M. Human genes escaping X-inactivation revealed by single cell expression data. *BMC Genomics* **20**, 201 (2019).
27. Garieri, M. *et al.* Extensive cellular heterogeneity of X inactivation revealed by single-cell allele-specific expression in human fibroblasts. *Proc. Natl. Acad. Sci.* **115**, 13015–13020 (2018).
28. Marks, H. *et al.* Dynamics of gene silencing during X inactivation using allele-specific RNA-seq. *Genome Biol.* **16**, 149 (2015).
29. Pajerowski, A. G., Nguyen, C., Aghajanian, H., Shapiro, M. J. & Shapiro, V. S. NKAP Is a Transcriptional Repressor of Notch Signaling and Is Required for T Cell Development. https://www.cell.com/immunity/abstract/S1074-7613(09)00185-X.
30. Mattson, M. P. & Meffert, M. K. Roles for NF-κB in nerve cell survival, plasticity, and disease. *Cell Death Differ.* **13**, 852–860 (2006).
31. Carlén, M. *et al.* Forebrain ependymal cells are Notch-dependent and generate neuroblasts and astrocytes after stroke. *Nat. Neurosci.* **12**, 259–267 (2009).
32. Gutierrez, H. & Davies, A. M. Regulation of neural process growth, elaboration and structural plasticity by NF-κB. https://www.cell.com/trends/neurosciences/abstract/S0166-2236(11)00038-5.
33. Yoon, K. & Gaiano, N. Notch signaling in the mammalian central nervous system: insights from mouse mutants. *Nat. Neurosci.* **8**, 709–715 (2005).
34. Lathia, J. D., Mattson, M. P. & Cheng, A. Notch: from neural development to neurological disorders. *J. Neurochem.* **107**, 1471–1481 (2008).
35. Fiordaliso, S. K. *et al.* Missense Mutations in NKAP Cause a Disorder of Transcriptional Regulation Characterized by Marfanoid Habitus and Cognitive Impairment. https://doi.org/10.1016/j.ajhg.2019.09.009 doi:10.1016/j.ajhg.2019.09.009.
36. Nilsson, S. *et al.* Mechanisms of Estrogen Action. *Physiol. Rev.* **81**, 1535–1565 (2001).
37. DeCasien, A. R. *et al.* Evolutionary and biomedical implications of sex differences in the primate brain transcriptome. https://doi.org/10.1016/j.xgen.2024.100589 doi:10.1016/j.xgen.2024.100589.
38. Di Bella, D. J. *et al.* Molecular logic of cellular diversification in the mouse cerebral cortex. *Nature* **595**, 554–559 (2021).

Supplementary Figures

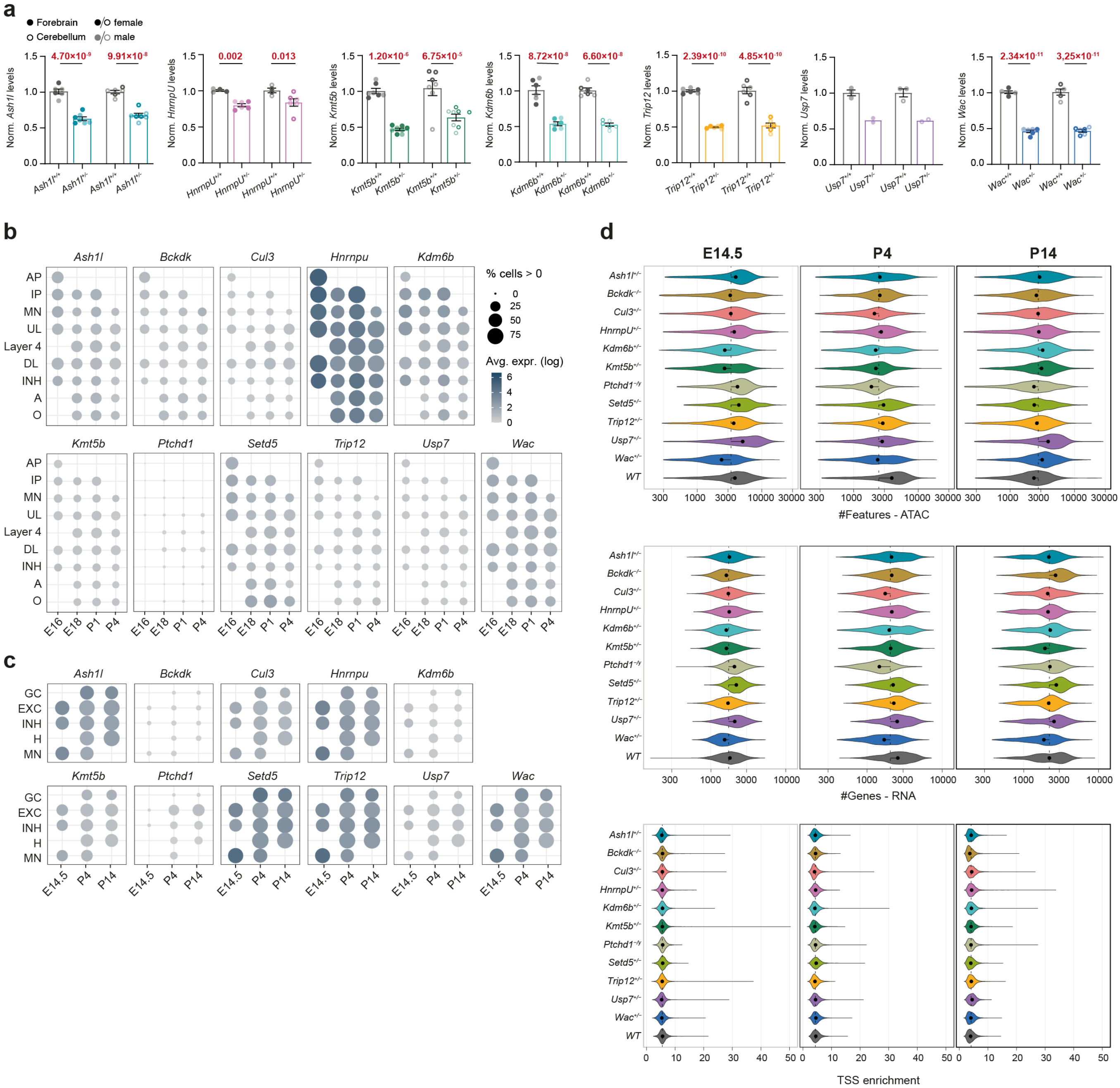

Supplementary Fig. 1: Expression of the selected ASD-risk genes and sequencing quality statistics.

a, Quantitative real-time PCR analysis of mRNA levels for ASD-risk genes in the respective mutants and littermate controls. mRNA levels were normalized to *Gapdh* and *Pgk1* (ΔCq expression values; *n= 8 (*$Kmt5b^{+/-}$*), n= 7 (*$Ash1l^{+/-}$*,* $Kmt5b^{+/+}$*), n= 6 (*$Ash1l^{+/+}$*,* $HnrnpU^{+/-}$*,* $Kdm6b^{+/+}$*,* $Kdm6b^{+/-}$*,* $Trip12^{+/+}$*,* $Trip12^{+/-}$*,* $Wac^{+/-}$*), n= 5 (*$Wac^{+/+}$*), n= 4 (*$HnrnpU^{+/+}$*), n= 3 (*$Usp7^{+/+}$*), n= 2 (*$Usp7^{+/-}$*);* 2-way ANOVA and Sidak's multiple comparison test). **b**, Average expression levels of the selected high-risk ASD genes in the murine cortex during perinatal development (E16, E18, P1, and P4) based on single-cell RNA sequencing data[38] (AP, apical progenitor; IP, intermediate progenitor; MN, migrating neuron; UL, upper layer excitatory neurons; Layer 4, layer IV excitatory neurons; DL, deeper layer excitatory neurons; INH, Inhibitory neurons; A, astrocytes; O, oligodendrocytes). Dot size indicates the percentage of cells expressing the gene

(> 0 counts); dot color indicates average log-normalized expression. **c**, Average expression of the selected high-risk ASD genes in the mouse cortex across embryonic and perinatal stages (E14.5, P4, P14) based on the present single-nucleus RNA sequencing data (GC, Glial cells; EXC, Excitatory neurons; INH, Inhibitory neurons; H, Hippocampus; MN, migrating neurons). Dot size and color as in b. **d**, Quality metrics for the number of genes sequenced in snRNA- and snATAC-seq and transcription starting site (TSS) enrichment per time point. The dotted lines represent the median values. Data are mean ± SEM in **a**. Animals used for analyses: *n (E14.5)*= 7 (*Cul3*$^{+/-}$, *Kdm6b*$^{+/-}$, *Trip12*$^{+/-}$), 6 (*Ash1l*$^{+/-}$, *Bckdk*$^{-/-}$, *HnrnpU*$^{+/-}$, *Kmt5b*$^{+/-}$, *Setd5*$^{+/-}$, *Usp7*$^{+/-}$, *Wac*$^{+/-}$, colony-matched controls), 3 (*Ptchd1*$^{y/-}$); *n (P4)*= 6 (*Ash1l*$^{+/-}$, *Bckdk*$^{-/-}$, *Cul3*$^{+/-}$, *HnrnpU*$^{+/-}$, *Kdm6b*$^{+/-}$, *Setd5*$^{+/-}$, *Trip12*$^{+/-}$, *Usp7*$^{+/-}$, *Wac*$^{+/-}$, colony-matched controls), 4 (*Kmt5b*$^{+/-}$), 3 (*Ptchd1*$^{y/-}$); *n (P14)*= 8 (*Ash1l*$^{+/-}$, *Cul3*$^{+/-}$), 7 (colony-matched controls), *6 (Bckdk*$^{-/-}$, *HnrnpU*$^{+/-}$, *Kdm6b*$^{+/-}$, *Kmt5b*$^{+/-}$, *Setd5*$^{+/-}$, *Trip12*$^{+/-}$, *Usp7*$^{+/-}$, *Wac*$^{+/-}$*)*, 3 (*Ptchd1*$^{y/-}$).

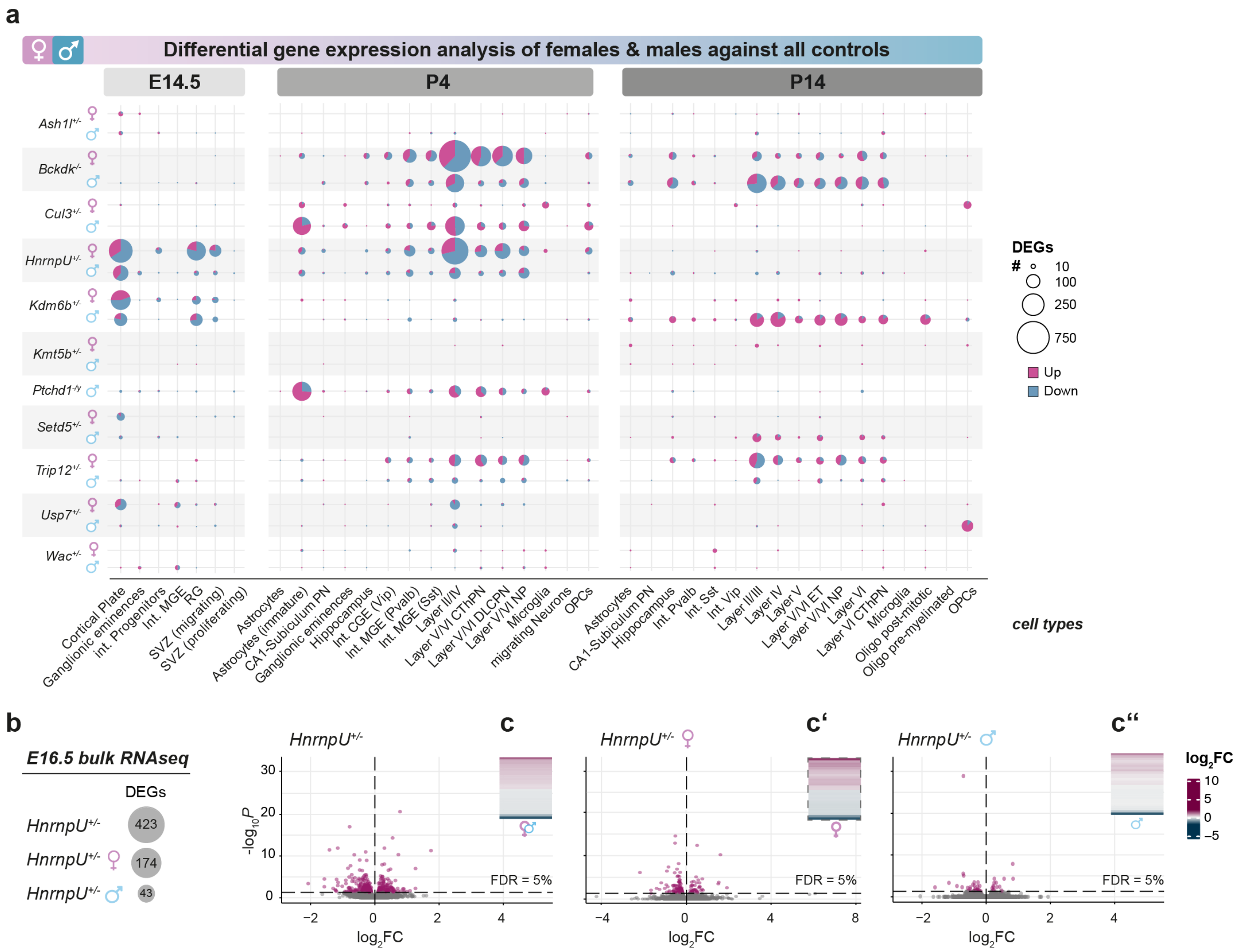

Supplementary Fig. 2: Gene expression analysis of female and male ASD mutants, bulk RNA-seq results confirm sex-specific gene expression changes. **a**, Number of differentially expressed genes (*p-adj.* < 0.05) in cell types across female and male ASD mutants in comparison to all (female and male) colony-matched controls collectively. Statistical significance was assessed using two-sided Wald test in DESeq2 with Benjamini–Hochberg correction for multiple testing. Animals used for analyses: *n (females; E14.5)*= 4 (*Cul3*$^{+/-}$, *Kdm6b*$^{+/-}$, *Trip12*$^{+/-}$), 3 (*Ash1l*$^{+/-}$, *Bckdk*$^{-/-}$, *HnrnpU*$^{+/-}$, *Kmt5b*$^{+/-}$, *Setd5*$^{+/-}$, *Usp7*$^{+/-}$, *Wac*$^{+/-}$, colony-matched controls); *n (males; E14.5)*= 3 per genotype; *n (females and males; P4)*= 3 per genotype; *n (females; P14)* = 4 (*Ash1l*$^{+/-}$, *Cul3*$^{+/-}$, colony-matched controls), 3 (*Bckdk*$^{-/-}$, *HnrnpU*$^{+/-}$, *Kdm6b*$^{+/-}$, *Kmt5b*$^{+/-}$, *Trip12*$^{+/-}$, *Usp7*$^{+/-}$, *Wac*$^{+/-}$), 2 (*Setd5*$^{+/-}$); *n (males; P14)*= 4 (*Ash1l*$^{+/-}$, *Cul3*$^{+/-}$, *Setd5*$^{+/-}$); 3 (*Bckdk*$^{-/-}$, *HnrnpU*$^{+/-}$, *Kdm6b*$^{+/-}$, *Kmt5b*$^{+/-}$, *Ptchd1*$^{y/-}$, *Trip12*$^{+/-}$, *Usp7*$^{+/-}$, *Wac*$^{+/-}$, colony-matched controls). **b**, Independent bulk RNA-seq analysis of E16.5 *HnrnpU* brains reveals that females exhibit a higher number of DEGs compared to males. **c-c''**, Heatmaps of the fold changes of a representative subset of DEGs significant in females show that the directionality of changes was similar in males. Animals used for bulk RNA-seq: *n*= 3 per genotype and sex.

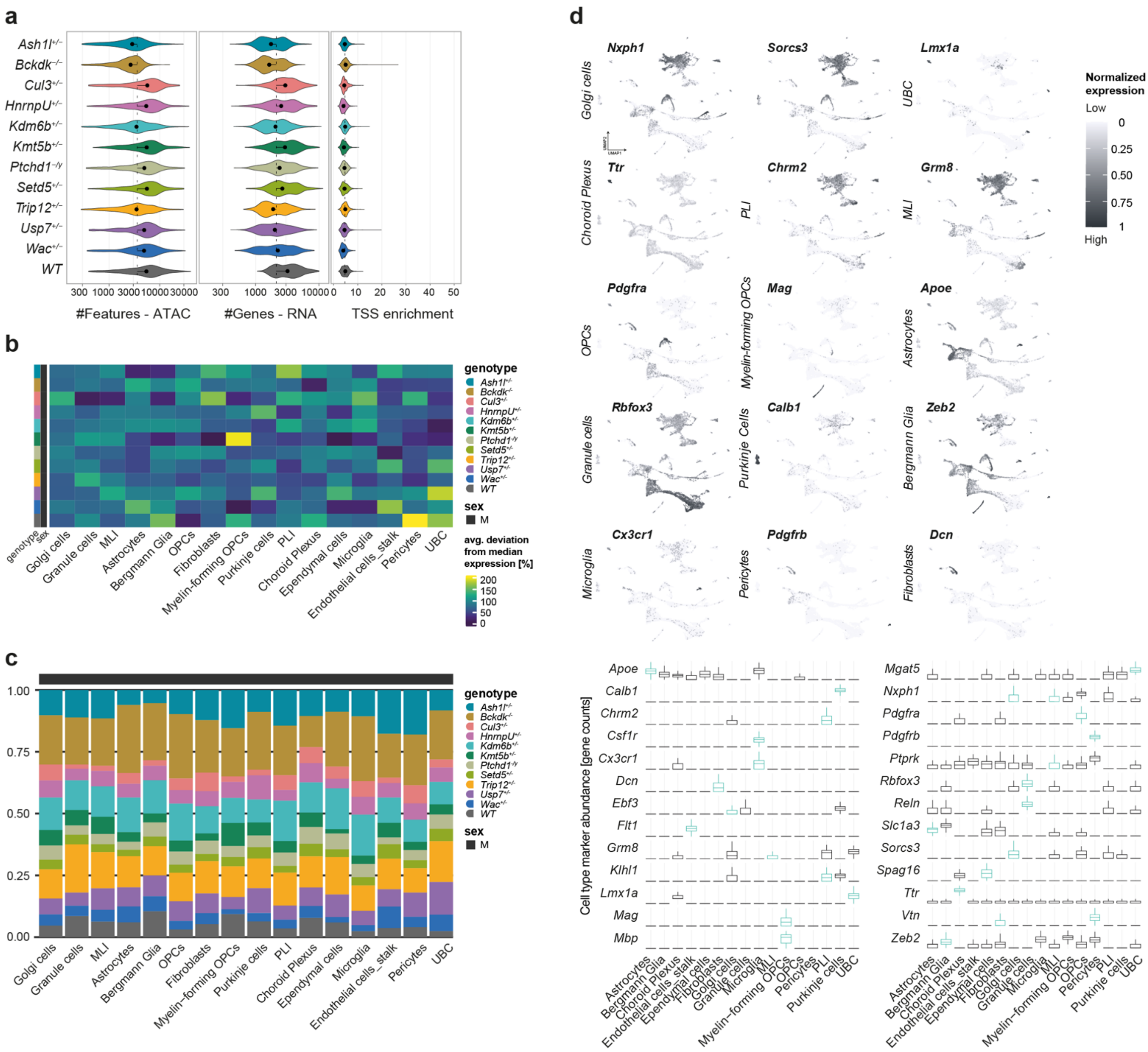

Supplementary Fig. 3: Sequencing quality metrics of perinatal cerebellar tissue. **a**, Quality metrics of the sequenced samples. **b**, Average deviation from the median expression of individual cell types of the ASD mutants at P4. Each tile in the heatmap shows the average expression deviation of each replicate relative to the median expression of all samples per cell type, expressed as a percentage. **c**, Relative proportion of cell types across mutants and colony-matched controls. **d**, UMAP visualization showing expression levels (top) of canonical molecular markers (bottom). Box plots show the abundance of selected cell type markers. Center lines represent medians, the box limits show the 25-75% interquartile range (IQR), whiskers extend to the furthest data points within 1.5 × IQR from the box edges. Animals used for analyses: *n*= 3 per genotype.

Supplementary Legends

Supplementary Table 1: Overview of multiplexing oligos, cluster annotation references used
T1, Table overview. **T2**, Sample-specific hashtag oligos (HTO) employing cholesterol-modification (CMO) used before pooling individual biological samples. **T3**, Sequences of i5 and i7 indices used to generate HTO libraries, unique sets of indices were used per library. **T4**, Overview of sample list, number of samples sequenced per time point. **T5**, Overview of sequences of all oligonucleotides used. **T6**, Reference datasets used for label transfer to support cell type cluster annotation. **T7**, List of most frequently identified DEGs across mutants and cell types at P4 across ASD mutants. **T8-T11**, Overview of number of nuclei analyzed per cell type, mutant, and time point in cortex and cerebellum.

Supplementary Table 2: Differentially expressed genes in RG1 cells at E14.5
T1, Table overview. **T2-12**, Differentially expressed genes identified through pseudotime analysis in RG1 cells across ASD mutants at E14.5.

Supplementary Table 3: Differentially expressed genes in individual cell types and ASD mutants across time
T1, Table Overview. **T2-T12,** Differentially expressed genes identified in individual cell types and ASD mutants across time. **T13**, Differentially expressed genes identified in all mutants collectively compared to colony-matched controls.

Supplementary Table 4: Differentially expressed genes in cell subclasses across time
T1, Table Overview. **T2-T12,** Differentially expressed genes identified in cell subclasses of individual ASD mutants across time. **T13**, Differentially expressed genes identified in all mutants collectively compared to colony-matched controls.

Supplementary Table 5: Enriched gene sets in cell subclasses of ASD mutants across time
T1, Table overview. **T2-T12**, Gene set enrichment analysis (GSEA) of individual ASD mutants in cell subclasses across time.

Supplementary Table 6: Enriched gene sets of individual cell types and ASD mutants across time
T1, Table overview. **T2-T12**, Gene set enrichment analysis (GSEA) of individual cell types and ASD mutants across time.

Supplementary Table 7: Distinct gene sets enriched in cell subclasses of ASD mutants across time
T1, Table overview. **T2-T13**, Gene set enrichment analysis (GSEA) of individual ASD mutants in cell subclasses across time. Individual ASD mutants are compared to all mutants collectively without including colony-matched controls.

Supplementary Table 8: SynGO annotation of synaptic-related genes in ASD mutants at P4 and P14.
T1, Table overview. **T2-T37**, SynGO annotation of synaptic-related genes in ASD mutants at P4 and P14.

Supplementary Table 9: Differentially accessible regions in individual cell types and ASD mutants across time
T1, Table Overview. **T2-T12,** Differentially accessible regions identified in individual cell types and ASD mutants across time. **T13**, Differentially accessible regions identified in all mutants collectively in comparison to colony-matched controls.

Supplementary Table 10: Differentially expressed genes between female and male wild-type control samples
T1, Table overview. **T2-T4**, Differentially expressed genes identified between female and male wild-type control samples.

Supplementary Table 11: Enriched gene sets in cell subclasses of female ASD mutants across time
T1, Table overview. **T2-T11**, Gene set enrichment analysis (GSEA) of individual female ASD mutants in cell subclasses across time.

Supplementary Table 12: Enriched gene sets in cell subclasses of male ASD mutants across time
T1, Table overview. **T2-T12**, Gene set enrichment analysis (GSEA) of individual male ASD mutants in cell subclasses across time.

Supplementary Table 13: Differentially expressed genes between female and male ASD mutants
T1, Table overview. **T2-T46**, Differentially expressed genes between female and male ASD mutants across cell subclasses. Individual sheets can be filtered for merged DEGs, fb/mbDEGs, and fs/msDEGs.

Supplementary Video 1: Cleared embryo head combined with EdU-labeling
EdU-labeling at E14.5 was combined with CUBIC clearing and imaged on a Zeiss Lightsheet microscope.

Supplementary Video 2: Example video of *Bckdk*$^{+/+}$ animal during gait recordings
Recording of a *Bckdk*$^{+/+}$ animal used for gait analysis, with stride renderings overlaid on the video frame. Video recorded at 60 fps.

Supplementary Video 3: Example video of *Bckdk*$^{-/-}$ animal during gait recordings
Recording of a *Bckdk*$^{-/-}$ animal used for gait analysis, with stride renderings overlaid on the video frame. Video recorded at 60 fps.
