# Supplementary material for: Cortical development dynamics across autism spectrum disorder mouse models
Source: Nature. 2026 Jun 17;656(8126):159–72. doi: 10.1038/s41586-026-10679-1 (PMC13441911; doi:10.1038/s41586-026-10679-1)
Supplement: Supplementary file 2 [file 41586_2026_10679_MOESM2_ESM.pdf]

nature portfolio

Corresponding author(s): Gaia Novarino

Last updated by author(s): Apr 22, 2026

# Reporting Summary

Nature Portfolio wishes to improve the reproducibility of the work that we publish. This form provides structure for consistency and transparency in reporting. For further information on Nature Portfolio policies, see our Editorial Policies and the Editorial Policy Checklist.

## Statistics

For all statistical analyses, confirm that the following items are present in the figure legend, table legend, main text, or Methods section.

| n/a | Confirmed | |
|---|---|---|
| ☐ | ☒ | The exact sample size (*n*) for each experimental group/condition, given as a discrete number and unit of measurement |
| ☐ | ☒ | A statement on whether measurements were taken from distinct samples or whether the same sample was measured repeatedly |
| ☐ | ☒ | The statistical test(s) used AND whether they are one- or two-sided<br>*Only common tests should be described solely by name; describe more complex techniques in the Methods section.* |
| ☒ | ☐ | A description of all covariates tested |
| ☐ | ☒ | A description of any assumptions or corrections, such as tests of normality and adjustment for multiple comparisons |
| ☐ | ☒ | A full description of the statistical parameters including central tendency (e.g. means) or other basic estimates (e.g. regression coefficient) AND variation (e.g. standard deviation) or associated estimates of uncertainty (e.g. confidence intervals) |
| ☐ | ☒ | For null hypothesis testing, the test statistic (e.g. *F*, *t*, *r*) with confidence intervals, effect sizes, degrees of freedom and *P* value noted<br>*Give P values as exact values whenever suitable.* |
| ☐ | ☒ | For Bayesian analysis, information on the choice of priors and Markov chain Monte Carlo settings |
| ☒ | ☐ | For hierarchical and complex designs, identification of the appropriate level for tests and full reporting of outcomes |
| ☐ | ☒ | Estimates of effect sizes (e.g. Cohen's *d*, Pearson's *r*), indicating how they were calculated |

*Our web collection on statistics for biologists contains articles on many of the points above.*

## Software and code

Policy information about availability of computer code

| | |
|---|---|
| Data collection | Multi-omics data were processed and quality-controlled using established bioinformatics software. Samples were de-multiplexed, and the raw sequencing data was converted to FASTQ format using bcl2fastq (v2.20.0.422). Joint processing of the gene expression and ATAC modalities was performed using the cellranger-arc software (v2.0.0). The mm10 reference genome was used for the alignment (refdata-cellranger-arc-mm10-2020-A-2.0.0, obtained from https://cf.10xgenomics.com/supp/cell-arc/refdata-cellranger-arc-mm10-2020-A-2.0.0.tar.gz). To capture CMO reads, the gene expression modality was separately processed using the cellranger count pipeline (v7.0.0 and v7.1.0), aligning it against the mm10 reference genome.<br>For bulk RNA-sequencing of HnrnpU embryonic samples, demultiplexed FASTQ files were filtered and trimmed using Cutadapt (v4.1) and aligned to the mm10 reference genome using STAR. For the analysis of ASD mutants (Bckdk-/-, Cul3+/-, HnrnpU+/-) and C57BL/6J colony-matched controls, demultiplexed FASTQ files were filtered and trimmed using Cutadapt (v4.1). Samples were aligned to the GRCm39 reference genome using STAR (v2.7.11b), and gene-level counts were obtained using FeatureCounts (v2.1.1).<br>ZEN Black imaging software (v3.10) was used for lightsheet imaging. Zeiss Arivis Pro software (v4.2.0) was used for analysing lightsheet images. Miniature neurotransmission was manually analyzed using Mini Analysis Program (Synaptosoft, v6.0.7). Passive and active membrane properties were analyzed using Clampfit (v11.3.0.02). Video acquisition for gait analysis was performed with OBS Studio (v31.0.1). |
| Data analysis | Data analysis was performed in R (v4.3.0 and v4.5.0) or Python (v3.10.5) using the following packages, Seurat (v4.3.0.9012), Signac (v1.10.0), targets (v1.2.2), renv (v1.0.2), MACS2 (v2.2.7.1), scDblFinder (v1.14.0), scuttle (v1.10.2), edgeR (v3.42.4), DESeq2 (v1.34.0 and v1.40.2), enrichplot (v1.20.1) clusterProfiler (v4.8.2), circlize (v0.4.15), Libra (v1.0.0), Palantir (v1.0.1), scFates (v1.0.6), scCODA Python package (v0.1.8), harmonypy (v0.0.5) and SnapATAC2 Python package (v2.3.0), statsmodels (v0.14.3), Cutadapt (v4.1), FeatureCounts (v2.1.1), RUVSeq (v1.42.0), SLEAP (v1.4.1), scipy library (v0.15.3) and TadPose library47 (v0.4.0). Other analyses softwares used include the EnrichmentMap plugin (v3.4.0) for Cytoscape (v3.10.1) and the online tool SynGO (v1.2). Cytometry gating and analysis were performed using Beckman |

Coulter's CytExpert software (v2.7). Scripts and analyses that support the main findings of this study are accessible in a GitHub repository under https://git.ista.ac.at/research-sofware/mouseome.

For manuscripts utilizing custom algorithms or software that are central to the research but not yet described in published literature, software must be made available to editors and reviewers. We strongly encourage code deposition in a community repository (e.g. GitHub). See the Nature Portfolio guidelines for submitting code & software for further information.

## Data

Policy information about availability of data

All manuscripts must include a data availability statement. This statement should provide the following information, where applicable:

- Accession codes, unique identifiers, or web links for publicly available datasets
- A description of any restrictions on data availability
- For clinical datasets or third party data, please ensure that the statement adheres to our policy

Single-nucleus multiomics data are available from GEO (accession number: GSE328363). The mm10 reference genome was used for the alignment (refdata-cellranger-arc-mm10-2020-A-2.0.0, obtained from https://cf.10xgenomics.com/supp/cell-arc/refdata-cellranger-arc-mm10-2020-A-2.0.0.tar.gz). Single-cell data can be accessed and visualized through a CELLxGENE database (https://adameykolab.hifo.meduniwien.ac.at/cellxgene_public/filecrawl/.2026_Nature_Schwarz).

## Research involving human participants, their data, or biological material

Policy information about studies with human participants or human data. See also policy information about sex, gender (identity/presentation), and sexual orientation and race, ethnicity and racism.

| | |
|---|---|
| Reporting on sex and gender | *Use the terms sex (biological attribute) and gender (shaped by social and cultural circumstances) carefully in order to avoid confusing both terms. Indicate if findings apply to only one sex or gender; describe whether sex and gender were considered in study design; whether sex and/or gender was determined based on self-reporting or assigned and methods used. Provide in the source data disaggregated sex and gender data, where this information has been collected, and if consent has been obtained for sharing of individual-level data; provide overall numbers in this Reporting Summary. Please state if this information has not been collected. Report sex- and gender-based analyses where performed, justify reasons for lack of sex- and gender-based analysis.* |
| Reporting on race, ethnicity, or other socially relevant groupings | *Please specify the socially constructed or socially relevant categorization variable(s) used in your manuscript and explain why they were used. Please note that such variables should not be used as proxies for other socially constructed/relevant variables (for example, race or ethnicity should not be used as a proxy for socioeconomic status). Provide clear definitions of the relevant terms used, how they were provided (by the participants/respondents, the researchers, or third parties), and the method(s) used to classify people into the different categories (e.g. self-report, census or administrative data, social media data, etc.) Please provide details about how you controlled for confounding variables in your analyses.* |
| Population characteristics | *Describe the covariate-relevant population characteristics of the human research participants (e.g. age, genotypic information, past and current diagnosis and treatment categories). If you filled out the behavioural & social sciences study design questions and have nothing to add here, write "See above."* |
| Recruitment | *Describe how participants were recruited. Outline any potential self-selection bias or other biases that may be present and how these are likely to impact results.* |
| Ethics oversight | *Identify the organization(s) that approved the study protocol.* |

Note that full information on the approval of the study protocol must also be provided in the manuscript.

# Field-specific reporting

Please select the one below that is the best fit for your research. If you are not sure, read the appropriate sections before making your selection.

☒ Life sciences ☐ Behavioural & social sciences ☐ Ecological, evolutionary & environmental sciences

For a reference copy of the document with all sections, see nature.com/documents/nr-reporting-summary-flat.pdf

# Life sciences study design

All studies must disclose on these points even when the disclosure is negative.

| | |
|---|---|
| Sample size | Sample size was based on established standards in the field; including a minimum of three independent biological replicates per genotype and sex were used as is common practice in the field. For single-nuclei multi-omics profiling a total of 251 samples across 45 multiplexing reactions was used. Each multiplexing reaction contained between 4-6 samples having sample specific barcodes. For each multiplexing reaction, a total of 3 10X libaries were prepped (ATAC, RNA, Hashtag oligo library).<br>For bulk RNA sequencing, 12 embryos of HnrnpU were used (n= 3 per sex + genotype), male and female litter-mate mice were used. For bulk RNA-sequencing animals of Bckdk, Cul3, HnrnpU mutants and colony-matched controls, n=3 per sex + genotype + timepoint), male and female mice were used. |

| | |
|---|---|
| Data exclusions | One sample of aKdm6b mutants was determined as outlier during outlier analysis and excluded from analysis for assessing Pdgfra+ cells. One sample used for bulk RNA-sequencing of HnrnpU embryos was excluded, as it clustered clearly away from all other samples during the preliminary quality assessment, samples from this mouse were excluded from further analyses. Two samples used for multi-omics analysis of Kmt5b mutants were excluded, as they clustered clearly away from all other samples during the preliminary quality assessment and was recognized as outliers. |
| Replication | In total we have investigated multiple genetic knockout animals, across three time points, sexes and brain regions. For multi-omics profiling and bulk RNA-sequencing, animals coming from at least 3 separate litters were collected to avoid potential litter-specific biases. For every sex, a minimum of 3 individual biological replicates were sampled. Our design included replication from individuals within the same line as well as replication with different knockout mutant lines. Additionally, we show high levels of correlations and replication of the multi-omics and bulkRNA seq dataset as shown in Extended Data Figure 3i. The individual "n"-values indicating the biological replicates per experiment are indicated either as data points or in the figure legends. |
| Randomization | Samples were randomly assigned for multiplexing reactions to avoid any genotype-specific representation in the mutli-omic multiplexing groups. Whenever possible, female and male counterparts of the same genotype were grouped together in the multi-omic multiplexing reactions. All wild-type samples were randomly assigned to multiplexing reactions. Tissues (cerebral cortex & cerebellum) and time points were handled separately and not grouped together for multiplexing. Processing of samples did not follow any particular order. The dataset was monitored for evidence of batch effects, but did not identify any technical covariates that should be controlled for. |
| Blinding | Investigators were not blinded during sample preparation for sequencing or during bioinformatics analyses, as genotype information was required for accurate sample pooling and downstream bioinformatic analysis. Investigators were blinded during image analysis, electrophysiological recordings and analysis, as well as during behavioral gait recordings. |

# Reporting for specific materials, systems and methods

We require information from authors about some types of materials, experimental systems and methods used in many studies. Here, indicate whether each material, system or method listed is relevant to your study. If you are not sure if a list item applies to your research, read the appropriate section before selecting a response.

## Materials & experimental systems

| n/a | Involved in the study | |
|---|---|---|
| ☒ | ☐ | Antibodies |
| ☒ | ☐ | Eukaryotic cell lines |
| ☒ | ☐ | Palaeontology and archaeology |
| ☐ | ☒ | Animals and other organisms |
| ☒ | ☐ | Clinical data |
| ☒ | ☐ | Dual use research of concern |
| ☒ | ☐ | Plants |

## Methods

| n/a | Involved in the study | |
|---|---|---|
| ☒ | ☐ | ChIP-seq |
| ☐ | ☒ | Flow cytometry |
| ☒ | ☐ | MRI-based neuroimaging |

## Animals and other research organisms

Policy information about studies involving animals; ARRIVE guidelines recommended for reporting animal research, and Sex and Gender in Research

| | |
|---|---|
| Laboratory animals | Animals were kept in the Preclinical Facility at the Institute of Science and Technology. Mice were housed in commercially available individually ventilated cages (IVCs) under defined standard laboratory conditions (room temperature 22± 1 °C, relative humidity 55 ± 10%) on a 12 h light/dark cycle (lights on at 7:00 am) and housed in groups of 3-4 animals per cage, with food and water available ad libitum. Experiments were carried out under specific pathogen-free conditions, and the health status of the mouse lines was routinely checked by a veterinarian. Wild-type animals C57BL6/J animals were used. All transgenic mouse lines were backcrossed into C57BL/6J background a minimum of two times. We used animals of both sexes at the developmental stages of embryonic day (E)14.5, E16.5, postnatal day (P)4 and P14. |
| Wild animals | No wild animals were used in this study. |
| Reporting on sex | Both female and male animals were used to identify sex-specific changes. |
| Field-collected samples | No field samples were collected in this study. |
| Ethics oversight | All animals used in this study were approved by the Institutional Animal Care and Use Committee at ISTA and by the Austrian Federal Ministry of Education, Science and Research (BMBWF). |

Note that full information on the approval of the study protocol must also be provided in the manuscript.

# Plants

Seed stocks

*Report on the source of all seed stocks or other plant material used. If applicable, state the seed stock centre and catalogue number. If plant specimens were collected from the field, describe the collection location, date and sampling procedures.*

Novel plant genotypes

*Describe the methods by which all novel plant genotypes were produced. This includes those generated by transgenic approaches, gene editing, chemical/radiation-based mutagenesis and hybridization. For transgenic lines, describe the transformation method, the number of independent lines analyzed and the generation upon which experiments were performed. For gene-edited lines, describe the editor used, the endogenous sequence targeted for editing, the targeting guide RNA sequence (if applicable) and how the editor was applied.*

Authentication

*Describe any authentication procedures for each seed stock used or novel genotype generated. Describe any experiments used to assess the effect of a mutation and, where applicable, how potential secondary effects (e.g. second site T-DNA insertions, mosiacism, off-target gene editing) were examined.*

# Flow Cytometry

## Plots

Confirm that:

☒ The axis labels state the marker and fluorochrome used (e.g. CD4-FITC).

☒ The axis scales are clearly visible. Include numbers along axes only for bottom left plot of group (a 'group' is an analysis of identical markers).

☒ All plots are contour plots with outliers or pseudocolor plots.

☒ A numerical value for number of cells or percentage (with statistics) is provided.

## Methodology

Sample preparation

Mouse cortical tissue of E14.5 embryos (pregnant dam was EdU-injected 2h prior) was isolated in ice-cold HBSS, meninges were removed and forebrain structures were dissociated using an established papain-DNAse protocol. Following dissociation, S-phase cells that incorporated EdU were detected by click chemistry. In brief, washed samples were fixed for 15 min at room temperature and permeabilized using the kit's supplied 1X saponin-based permeabilization and wash buffer. Two washes (1% BSA in PBS, 300g for 10 min each) were performed between fixation and permeabilization. Following fixation and permeabilization, the click reaction was prepared according to the manufacturer's protocol, and cells were incubated for 30 min at room temperature, protected from light. Samples were washed twice with supplied 1X saponin-based permeabilization and wash buffer and centrifuged at 300g for 10 min per wash.

Instrument

Samples were analyzed on a Beckman Coulter Cytoflex LX.

Software

Gating and analysis were performed using Beckman Coulter's CytExpert software (v.2.7).

Cell population abundance

Cells population abundance was analysed, but not sorted for further experiment. Positive cell populations were analysed according to EdU-positive gating. For each experiment, a control sample was processed identically up to the permeabilization step and used as negative control for gating negative populations in cytometry analysis. 50,000 total events were acquired per sample.

Gating strategy

Cell populations were gated based on forward scatter (FSc) versus side scatter (SSc), and doublets were excluded via FSc area versus height gating.

☒ Tick this box to confirm that a figure exemplifying the gating strategy is provided in the Supplementary Information.
