# Supplementary material for: Cortical development dynamics across autism spectrum disorder mouse models
Source: Nature. 2026 Jun 17;656(8126):159–72. doi: 10.1038/s41586-026-10679-1 (PMC13441911; doi:10.1038/s41586-026-10679-1)
Supplement: Supplementary file 3 [file 41586_2026_10679_MOESM7_ESM.pdf]

nature portfolio

Peer Review File

Cortical development dynamics across autism spectrum disorder mouse models

Corresponding Author: Professor Gaia Novarino

Any redactions in this file are there to maintain patient confidentiality, the confidentiality of unpublished data, or to remove third-party material.

This file contains all reviewer reports in order by version, followed by all author rebuttals in order by version.

Attachments originally included by the reviewers as part of their assessment can be found at the end of this file.

Version 0:

Reviewer comments:

Referee #1

(Remarks to the Author)
Whole exome sequencing studies of autism spectrum disorder have identified >100 ASD susceptibility genes with high confidence, each consisting of a gene of large effect where protein-truncating mutations confer significant risk. ASD susceptibility genes are preferentially expressed in the developing brain and have roles in chromatin regulation and synaptic function. However it remains unclear what are the key pathways, cell lineages and developmental stages that are disrupted in autism. This knowledge is difficult to infer without a broad understanding of the molecular pathways that are under the regulation of this wide variety of genes.
The same genes also have relatively large effects in rodent and iPSC models making them ideal targets for characterizing the effects of genes on the developing brain. To date, however, most studies have done characterization of mouse models one-gene-at-a-time looking broadly at the effects of a single gene on molecular and cellular function and behavior. From such studies, it is difficult to infer what are the common pathways across multiple genes, that presumably form some core aspects of ASD. The traditional throw-the-kitchen-sink-at-one-gene-at-a-time fails to consider a fundamental question about the pathogenesis of disease: of all of the functional effects that this gene might have, how does it relate to autism? Of the many behavioral phenotypes that this mouse model might exhibit, which of these cognitive readouts are most related to the human condition? For complex neurodevelopmental disorders that are linked to many genes a logical approach is, rather than of publishing 11 mouse models one-model-at-a-time in 11 different papers, instead to carry out each analysis as a systematic comparative study of all 11, but with a narrower application of platforms (snRNA sequencing) and scientific focus (effects on gene regulation and progression of cell lineages). Such a comparative study allows the investigators to evaluate how genes CONVERGE on common phenotypes and pathways and conversely where do the genetic effects DIVERGE significantly between mouse models (which are sobering results that indicate how molecular and cellular mechanisms may differ by gene).
The study by Novarino and colleagues is refreshing in this respect. This study has applied single-nucleus multi-omics sequencing to characterize the genetic effects on molecular pathways and the development of cell lineages in forebrain from mouse models of 11 ASD susceptibility genes at E14.5, P4 and P14. Nearly all of which are involved in chromatin, regulation of gene expression or cell signaling (Ash1l, Cul3, HnrnpU, Kdm6b, Kmt5b, Ptchd1, Setd5, Trip12, Usp7 or Wac), One exception is Bckdk which is kinase that directly regulates ketone metabolism.
Perhaps the most dramatic finding was a remarkably consistent trend in forebrain cells lineages across multiple mouse models demonstrating, at E14.5, an increase in radial glia cells and corresponding decrease in cortical plate cells. Strong effects were observed in Cul3, HnrnpU, Kdm6b, and Ptchd1 models and weak trends were observed in other models. If this finding is mostly robust, it suggests that delayed progression of the radial glia cell lineage is a “convergent” effect of multiple ASD genes.
Differential expression of genes was investigated in pseudobulk RNA from specific cell types. With regard to the total number of DEGs, the strongest effects were observed in P4 layer II/IV neurons. This pattern was surprisingly similar across all mutants. Several synaptic proteins, ion channels and cytoskeletal proteins were found to be differentially expressed across which included several genes implicated in ASD. This type of evidence for convergence has been seen before in other mouse models of ASD genes, but this the first evidence pointing to relatively small very specific set of genes that are

consistently dysregulated in both period and cell type across many mutants. There were also many DEGs that were unique to each mutant. The overall interpretation is basically "we see commonalities and we see differences", which is a bit unsatisfying. Overall effects of genes on expression were stronger in females. A subset of genes that showed female biased differential expression were identified, and subset of these were observed in multiple mouse models. Sex biased DEGs were validated by bulk RNASEq of one line hnrnpU E16.5. Gene expression and chromatin accessibility were positively correlated for genes and gene sets.
I assert that the convergent findings are of high significance IF THEY ARE ROBUST. I do have some questions and concerns about the overall design and approach, that I think should be addressed to fully assess their credibility. Overall a more rigorous analytical approach to quantifying convergence and divergence of genes and pathways among differentially expressed genes is needed.
1. Mouse cohort breeding. Each time point included 3 mice per genotype per sex, which were drawn from 3 different litters. Were matched littermate controls used for each mouse model? (This is mentioned for the bulk RNA-Seq but was not described for the scRNA samples). Or were all mouse models compared to a single control group of 3 per genotype per sex (which would generally not be littermates, would have been bred separately and would not be analyzed in parallel with all mouse models)? IF it is the latter, this would present some challenges. If controls were derived from separate litters than the mouse models, there is potential for there to be a few rare coding mutations in that line that can accumulate over a couple generations. In addition, genes or pathways that form outliers in a single small group (N = 3 male, 3 female) of controls, would appear as convergent cell type effects and convergent DEGs relative to most of the mouse models. It would be difficult to distinguish idiosyncratic characteristics of a small control group from "convergent" effects of ASD genes. Again, this would not be a major concern if the controls were drawn from littermates of several different mouse models… and they were not bred and analyzed separately.
2. Not sure what the guidelines are at nature but it's generally a good idea to include the numbers of wt an control animals per group in the figure legends.
3. Is the reduction in CP cells and increase in CP cells consistent with other published mouse models? Or Human models? Some studies do show an effect that contrasts with this (but are zeroing in on the same process). For instance human brain organoid models at mid-to-late fetal period of 16p11.2 deletion show reduced numbers of neural progenitors and increased numbers of mature neurons (PMID: 34433918, PMID: 36826401).
4. Not sure how to interpret the convergence and divergence of DEGs between mutants. The analysis of molecular pathways seems view all DEGs that overlap between mouse models as "convergence" or "independent validation of the DEGs" and all DEGs that do not overlap were viewed as "divergence". Without a more rigorous analysis of the molecular convergence of these 11 mutants this kind of overlap is hard to interpret. There are statistical methods for determining if overlap between 2, 3, 4, 5… sets of DEGs is significant. See for example the R package SuperExactTest. In addition, There are computational methods for quantifying convergence of gene sets at the network level by network colocalization (PMID: 36653526) that may be more powerful than quantifying overlaps of specific genes. It feels like the authors are basically giving a broad description of the observed "convergence" but without a rigorous statistical analysis the reader is left to sort of draw their own conclusions.
5. Is there a plausible explanation for why all mutants show a nearly identical pattern of strong differential expression of genes in P4 excitatory neurons and comparatively weaker effects at E14.5. Why do you suppose phenotypic effects were more concentrated in E14.5 but effects on gene expression were not?
6. And then in comparing all strains to wild type finds exactly 2 genes with very large differential expression. Rsrp1 galnt17. Is this pseudobulk of all samples vs wild type? These 2 genes stand out so much, they look almost like they were CRISPR'd. Were these significant DEGs in any of the individual mutants? Or only in the combined data? Can you rule out (1) off target effects in these genes AND (2) coding mutations that accumulated in the BL6J line before breeding the controls for this experiment.
7. Cell type pseudobulk of all mutants vs wild type. Fig 2A. The cell types that have the greatest number of DEGs are consistently excitatory neurons at P4 and to a lesser extent inhibitory neurons. Are the genes that are being modeled in these mutants expressed predominantly in P4? Is
8. "At P4, mutants displayed a marked convergence on pathways related to synaptic and ion channel functions (Fig. 2d, bottom).The blue color of all the nodes in this network in Fig 2d appear to correspond to one gene in the legend (Wac). Were the nodes in this network supposed to be color-coded by gene? From this figure, I can't infer anything about convergence. Extended data figure 4b does have color coded circles around the clusters in the enrichment map and the gray clusters are shared by a majority of mutants. But the colors in the figure are hard to match to the legend.
9. The enrichment maps in Figure 2d do not do a great job of illustrating the relationship of the various mouse models to pathways. Extended data figure 5 does a better job of illustrating the direction of the effects and linking them to specific mouse models. This shows downregulation of synaptic transmission and upregulation of RNA processing and translation as a convergent mechanism. But the results are still fragmented into separate plots for each cell type and developmental period. Wouldn't it be possible to define a variety of gene sets that correspond to the major categories in Figure 2d, and then to create heatmap illustration that combines the normalized enrichment scores of the gene sets across all mouse models, cell types and periods? I would anticipate that this would highlight various points of convergence and divergence between mouse models in a more unified way, and the reader would not need to look for meaningful similarities and differences across multiple figure panels. Ideally, this kind of heatmap would NOT have missing values for nonsignificant enrichments. Significance could be indicated by varying some other feature of the heatmap (size, shape, etc.)
10. "Despite some commonalities, each mutant showed a unique combination of affected processes, suggesting that every mutation leads to a distinct synaptic state (Fig. 3a')" how does one quantify the uniqueness of the plots in Fig 3a'? If the results were NOT unique for each mutant, what exactly would that look like? Even replicate experiments of the same mouse model are not going to produce identical results. There would be differences. What does the null expectation look like? What is it about the single gene heatmaps in Fig 3d,e that make them "unique" and how are they are more unique than the "convergent" patterns for ion channels in Fig 2b,c?

Referee #2

(Remarks to the Author)
In this manuscript, “Developmental dynamics of the cortical cellular and molecular landscapes in autism spectrum disorder”, Schwarz and colleagues aim to understand how so many genes functioning in different molecular mechanisms lead to somewhat similar phenotypes in autism spectrum disorder (ASD). To do this, they performed single nucleus multiome sequencing coupling gene expression profiles and chromatin landscape from the cortex (and in some cases cerebellum) of 11 different mouse models of ASD at three different timepoints in development. They found that the studied mutants have a delay in the radial glia lineage progression, that gene expression changes were most prominent at postnatal day 4, affecting essential synaptic and neurotransmission-related pathways, that the chromatin landscape was most impacted at embryonic day E14.5, that there were sex-specific differences, and that the studied ASD genes converge on essential neurodevelopmental pathways, while also affecting pathways unique to each gene.

Overall, the study is extensive and is the first to look at 11 mouse models of autism simultaneously, while aiming to find pathways of convergence. Strengths of the study include addressing a central question in ASD research about the extent of molecular and cellular convergence of distinct mutations, and employing systematic and unbiased analysis of multiple ASD mutations simultaneously. However, several major and minor points need to be addressed to increase the rigor and impact of this work.

Major points:

1. Our major concern is that none of the single nuclei multiomics conclusions are followed up by validation, including the major conclusion about a developmental delay affecting the radial glia lineage. This means that all the results and conclusions are entirely based on one experiment. Single nuclei results need to be confirmed with other methods: starting with the cell abundance results in the different mutants, the authors can confirm their data with quantitative single molecule fluorescence in situ hybridization or they can perform immunostaining for markers of the affected cell type at the different timepoints. The delay in radial glia lineage progression (representing some of the strongest evidence for convergence across ASD mutations) is a strong claim from single nuclei data alone and needs to be validated as well: tissue staining, more sophisticated proliferation analysis (e.g., with BrdU labeling), and/or flow cytometry showing increase in the RG1 cells in mutants, and experiments assessing proliferation of the RG in the mutant to confirm that the mutant RG remain in their proliferating RG1 state, are necessary. Similarly, one of the main conclusions of this paper is the disrupted synaptic function, the authors could validate those findings with electrophysiological recordings in the affected mouse models compared to controls.

2. For the ATAC portion of the analysis, mainly fig.4, the authors need to provide more insights and interpretations of their results: why are HnrnpU and Kdm6b mutants the ones with the significantly higher DARs than other genes? How do you explain Setd5 mutants not showing nearly as much DAR although it is a chromatin remodeler itself? What makes Kdm6b different than the other histone modifiers mutants studied in this paper that it shows that many DARs, whereas the others don’t? Given what we know about the chromatin landscape and gene expression changes, how do you explain DARs mostly present at E14.5 compared to DEG at P4? At the technical level, is the cell coverage sufficient for differential accessibility analysis – the authors note the use of nominal p-values due to the lower signal to noise and the TSS enrichment looks a bit lower than expected.

3. For the sex-specific effect, the data are also lacking proper and deeper interpretation. Given that the mutations studied are rare, and that they affect both males and females, the observed results in this paper are interesting and very informative to understand the sex differences, but they don’t necessarily help explain the sex bias seen in ASD diagnosis. The data should be interpreted as differences in males and females with mutations, not as explaining the sex bias. The authors should also reflect more on the larger effect size seen in females and what it could mean for females affected by these mutations.

Other points

1. I appreciate the pseudobulk analysis for differential expression. However, the text states that “Given the nature of the pseudo-bulk analysis, the number of sequenced cells per cluster was not expected to influence these findings.” I wonder if this is true, because the Extended data fig 2a-c heatmaps look by eye to show more divergence from median for the lower frequency cell types. At a statistical level, wouldn’t sequencing more cells from a particular cell type and genotype increase the precision of these measures compared with low cell count clusters? I would at least be careful of the above statement.

2. The reduced ability to detect low abundance genes is noted in the limitations. However, I am still concerned about the related issue of how the reduced power to detect differential expression among low expressed (but detected) genes would affect the ontology enrichments (that constitute a major portion of the manuscript).

This issue is not unique to this study (which certainly meets or exceeds analysis standards in the field in many areas), but my guess is that significant quantitative differentially expressed genes are skewed towards higher abundance. Because higher abundance genes likely belong to different ontology categories than the background set of all detected genes, this skew could have strong effects on enrichment results. As such, the authors consider a background set and/or permutation

test that accounts for the distribution of expression levels at which DE genes are discovered. I mention this in part because the ontology enrichments are such a major piece of this manuscript.

I do appreciate that in a few cases, the ATAC data provides independent support by showing similar enrichments without being as affected by variation in expression levels. However, it is not clear how enrichments are performed for the ATAC data. Are these enrichments simply done at the gene level for genes linked to differentially accessible peaks? If so, couldn't this introduce a bias, because longer genes and genes with longer intergenic distances are more likely to have a linked differentially accessible peak?

3. Ambient RNA contamination has been shown to be a major issue in single nuclei data sets (https://doi.org/10.1016/j.neuron.2022.09.010), especially when the phenotype detected relate to glial cell types, since they are the ones primarily contaminated with ambient RNA. The experimental design to collect data in a common batch helps to minimize confounds between ambient RNA and particular genotypes. Nevertheless, the authors could consider tools like Cellbender (https://doi.org/10.1038/s41592-023-01943-7) to clean up nuclear ambient RNA contamination, and they should plot the intronic reads ratio (against UMI bins) to show that none of the analyzed cell types have a low intronic reads ratio, indicator of non-nuclear ambient RNA contamination, all of this prior to plotting the data and drawing conclusions.

[decontX https://genomebiology.biomedcentral.com/articles/10.1186/s13059-020-1950-6, soupx]

4. The authors should provide more clear discussion/visuals on the number of nuclei analyzed (post-filtering) for each mutant and sex alone (not just the total number) as this would help assess if sufficient cell coverage has been achieved across conditions and also to add transparency to interpretations of cell abundance differences (like in fig1.e).

Minor points:

1. For the pseudotime analysis, in fig.1h, the authors need to show the UMAPs of the control and mutants side-by-side to be able to visualize the differences in trajectories and to see the difference in RG1/RG2 between control and mutants. Also, please indicate which cells were included in the pseudotime analysis, and the choice of the starting point of the trajectory.

2. Some figures are hard to follow for the reader, please find a clearer way to present the data:
fig.1b-c and most UMAPs in the paper: the colors of the clusters and labels are very hard to distinguish and identify, consider drawing circles around the clusters or labels on top of the clusters as an extended figure;
fig.2d: the figure is complicated and not all the pathways need to be present in the figure, consider focusing on the ones highlighted;
fig.4b: different dot shapes and colors are overlapping and hard to follow, highlight the ones that are relevant;
extended fig.7: can barely see the dashed rectangles, make them more noticeable;
extended fig.4: very dense, simplify; extended fig.8d: the colors are extremely faint and cannot see them on top of the UMAPs.

3. Fig.6a should be part of extended figures, it does not fit with the goal of fig.6.

4. Fig.6c and extended fig.2a-c, the text says that "the samples and genotypes were similarly distributed across clusters", however the figures do not show all the samples. For simplicity the current figures are ok, but it would be nice to have one figure panel that shows all the samples/replicates, to make sure the replicates are similar, and that not a certain sample/replicate is driving the differences detected. The authors could also provide a stacked bar plot or other visuals to indicate the representation of genotypes in each cluster. Also in these same figures, the order of the samples does not match the order of the samples' labels for WAC mutant and WT, flip the samples on the heatmap to match the label and have the WT at the bottom. Also, fig.6c only shows males, show both males and females.

5. In fig.1i and j, the authors only show RG1 in each mutant and all mutants, include RG2 as well.

6. Some p values are missing from legends and text (ex fig.1j), please include all p values. The fold change cutoff for all differential gene expression is missing, please include in text and legends and justify the choice.

7. The text referring to fig.2d does not mention chromosomal segregation in excitatory neurons at E14.5, but it is the biggest colored cluster and the legend says that colored clusters are mentioned in results. Please add.

8. In extended fig. 7, the authors say "several mutant lines showed deregulation of axogenesis and dendrite morphogenesis-related genes, with the directionality of these changes varying between mutants. DEGs associated with these processes were upregulated in PV+ and Vip+ interneurons in most ASD mutants, while they were downregulated in excitatory Layer II/IV neurons." The authors should also include Sst+ cells as they show high upregulation as well.

9. In extended fig.10a, the authors say "When analyzing all mutants collectively, we found a strong correlation in Layer II/IV neurons at P4", the author should also include the strong correlation at P4 in immature astrocytes.

Version 1:

Reviewer comments:

Referee #1

(Remarks to the Author)
The authors have nicely addressed a majority of my concerns. One of my main concerns has been partially addressed, but additional genes should validated to substantiate claims of convergence of the RG phenotype
My main technical concern (comment #1) was in 2 parts. Concern #1A was a question about the breeding which the authors have nicely addressed. The additional details were very helpful. Concern #1B was a more general one that the evidence for convergence relies on comparing all mutants to a single small control group of 3 wild type males and 3 females. A random statistical fluctuation in the controls will be indistinguishable from a convergent effect of multiple gene mutations. The authors have partly addressed this concern by validating a main finding (increased RG numbers) in a 2nd set of HnrnpU mutant and sibling controls. This response is encouraging. There is reproducible evidence that RG cell numbers are increased in some mutations. The finding replicates in 1 gene (I won't quibble about the p-value of 0.08, but again statistical power of the small Ns is a persistent issue). Also, in the original manuscript differentially expressed genes were validated in several mutants and littermate controls, which is a convincing type of validation. Comparison of all mutants to a single small control group is STILL the basis for the claims of the convergent RG phenotype. To be honest a more convincing result would be one where the effects on RG differ between genes, which would suggest that the pattern is clearly related to the effects of gene mutations.
I recognize that this study was a huge amount of work. And to address this issue would require an even greater amount of work. At a minimum, I think the authors need to acknowledge that there is are key limitations in their experimental design.
1. Mouse studies done on this scale are very costly. Cost saving measures are required including (A) using small Ns per genotype which limits power to detect effects. Thus statistical evidence in Figure 1 is generally modest. Also (b) a small N of common controls has an additional limitation…
2. Random statistical variability in controls will produce patterns that will not look like noise. It will look like convergence. This design necessitates a fair amount of validation to substantiate claims of convergence. To make the evidence convincing, they might try expanding their validation to 2 more genes (such as KDM6B, Cul3 which had effects that were similarly strong)… similar to what was done to validate DE genes. Larger Ns of mutant and littermate controls are recommended. Given modest effects that replicated for HNRNPU, Ns of ~15 per group might be needed.

Referee #2

(Remarks to the Author)
I applaud the authors for the detailed revisions and for addressing all major comments.

In particular, the conclusions are strengthened by:
-The more sophisticated analyses of convergence and the consideration of independent control cells in these analyses
-The histological validation of delayed differentiation and altered proliferation
-The correlation between transcriptomic changes and subtle electrophysiological alteration.

Overall, this is an important study with a well-reasoned conceptual framework to systematically analyze many loss-of-function ASD-linked genes together in mouse models, and the analyses add to our knowledge about existing genes and illuminate the potential for transient convergence, while providing a model for future studies.

Referee #4

(Remarks to the Author)
I co-reviewed this manuscript with one of the reviewers who provided the listed reports.

Version 2:

Reviewer comments:

Referee #1

(Remarks to the Author)
The authors have addressed my main concern regarding evidence for convergence of the RG proliferation phenotype by replicating the phenotype for not only HnrnpU but also for a 2nd gene Kdm6b. While the required N's were hard to come by, it's clear that they were needed. I have no other significant concerns with this paper.

Just a minor point. The first sentence of the abstract concludes with a controversial premise "Recent research has identified over 100 causal genes in autism spectrum disorder, raising the question of how *mutations in genes with diverse functions result in similar clinical presentations*".
The clinical presentations of people within the autism spectrum are diverse. Likewise, the clinical presentations of various gene mutations are diverse. The association of 100 genes with a heterogeneous categorical trait does not mean that they generally present with similar neurobehavioral and psychiatric traits, but they must have SOME things in common that are relevant to social behavior.

A variation on this premise that is more consistent with our current understanding would be: "While 100 autism risk genes have diverse functions, phenotypes that converge across multiple gene models could reveal the shared neurobiological processes relevant to autism."

Open Access This Peer Review File is licensed under a Creative Commons Attribution 4.0 International License, which permits use, sharing, adaptation, distribution and reproduction in any medium or format, as long as you give appropriate credit to the original author(s) and the source, provide a link to the Creative Commons license, and indicate if changes were made.
In cases where reviewers are anonymous, credit should be given to 'Anonymous Referee' and the source.
The images or other third party material in this Peer Review File are included in the article's Creative Commons license, unless indicated otherwise in a credit line to the material. If material is not included in the article's Creative Commons license and your intended use is not permitted by statutory regulation or exceeds the permitted use, you will need to obtain permission directly from the copyright holder.
To view a copy of this license, visit https://creativecommons.org/licenses/by/4.0/

We thank the reviewers for their thoughtful and constructive comments. Following these recommendations, we have conducted extensive additional analyses and experiments to address all points raised. While this meant extended revision time, we believe that the new data have further strengthened the significance of our findings.
To facilitate the review process, we provide a summary table of all updated figures and datasets (Revision Table 1), along with a detailed, point-by-point response to each reviewer comment. Substantial revisions in the *Results, Methods, and Figure Legends* sections are highlighted in blue throughout the manuscript for ease of review.

Revision Table 1: Summary list of figure changes.

Figure	***Changes/ Additions***	***Analyses/ Experiments***	***Answer to Reviewer***
Figure 1	b-d	Modification of UMAP readability	R2
Figure 1	i (**new**)	RG1 & RG2 pseudotime	R2
Figure 2	c, d (**new**)	Network colocalization and overlap analysis (excitatory neurons)	R1
Figure 3	**new**	Electrophysiology analysis	R2
Figure 4	b	Readability of figure	R2
Figure 5	b (**new**)	New gene set enrichment analysis	
Figure 6	a	Modification of UMAP readability	R2
Figure 6	e-g (**new**)	Motor behavior in *Bckdk* and *Kmt5b* mutants	R2
Extended Data Fig. 1	b, c (**new**)	Expression pattern of the selected genes	R1
Extended Data Fig. 2	d-f (**new**)	Visuals to indicate the representation of genotypes in each cluster	R2
Extended Data Fig. 3	d, d' (**new**)	Validation of cell type abundance in OPCs and imm. astrocytes	R2
Extended Data Fig. 3	e (**new**)	EdU labeling/validation of expanded pool of RG cells	R1 & R2
Extended Data Fig. 4	c (**new**)	Downsampling analysis to control for sequencing depth	R1 & R2
Extended Data Fig. 4	f (**new**)	Statistical testing of DEG overlaps	R1
Extended Data Fig. 4	i (**new**)	Evaluation of reproducibility using additional datasets	R2
Extended Data Fig. 8	a (**new**)	New gene set enrichment analysis	
Extended Data Fig. 9	**new**	Network colocalization and overlap analysis (inhibitory neurons)	R1
Extended Data Fig. 10	**new**	Electrophysiology validation	R2
Extended Data Fig. 14	c (**new**)	Visuals to indicate the representation of genotypes in each cluster	R2
Revision Fig. R1.1		Breeding scheme employed	R1
Revision Fig. R1.2		Transcriptomic correlations across datasets	R1
Revision Fig. R1.3		Gene set enrichment validation	R1
Revision Fig. R1.4		Confounding factor analysis of metadata	R1

Revision Fig. R1.5		Sequencing quality metrics of datasets	R1
Revision Fig. R1.6		Cell type abundance changes in *HnrnpU* mutants.	R1
Revision Fig. R2.1		Cell type abundance changes in *HnrnpU* mutants	R2
Revision Fig. R2.2		Transcriptomic correlations across datasets	R2
Revision Fig. R2.3		Correlation gene-body differential accessibility *p*-values and corresponding gene lengths	R2
Revision Fig. R2.4		Quality metrics showing intronic read ratios	R2

Point-to-point response to reviewers' comments:

Referee #1 (Remarks to the Author):

Whole exome sequencing studies of autism spectrum disorder have identified >100 ASD susceptibility genes with high confidence, each consisting of a gene of large effect where protein-truncating mutations confer significant risk. ASD susceptibility genes are preferentially expressed in the developing brain and have roles in chromatin regulation and synaptic function. However it remains unclear what are the key pathways, cell lineages and developmental stages that are disrupted in autism. This knowledge is difficult to infer without a broad understanding of the molecular pathways that are under the regulation of this wide variety of genes.
The same genes also have relatively large effects in rodent and iPSC models making them ideal targets for characterizing the effects of genes on the developing brain. To date, however, most studies have done characterization of mouse models one-gene-at-a-time looking broadly at the effects of a single gene on molecular and cellular function and behavior. From such studies, it is difficult to infer what are the common pathways across multiple genes, that presumably form some core aspects of ASD. The traditional throw-the-kitchen-sink-at-one-gene-at-a-time fails to consider a fundamental question about the pathogenesis of disease: of all of the functional effects that this gene might have, how does it relate to autism? Of the many behavioral phenotypes that this mouse model might exhibit, which of these cognitive readouts are most related to the human condition? For complex neurodevelopmental disorders that are linked to many genes a logical approach is, rather than of publishing 11 mouse models one-model-at-a-time in 11 different papers, instead to carry out each analysis as a systematic comparative study of all 11, but with a narrower application of platforms (snRNA sequencing) and scientific focus (effects on gene regulation and progression of cell lineages). Such a comparative study allows the investigators to evaluate how genes CONVERGE on common phenotypes and pathways and conversely where do the genetic effects DIVERGE significantly between mouse models (which are sobering results that indicate how molecular and cellular mechanisms may differ by gene).
The study by Novarino and colleagues is refreshing in this respect. This study has applied single-nucleus multi-omics sequencing to characterize the genetic effects on molecular pathways and the development of cell lineages in forebrain from mouse models of 11 ASD susceptibility genes at E14.5, P4 and P14. Nearly all of which are involved in chromatin, regulation of gene expression or cell signaling (Ash1l, Cul3, HnrnpU, Kdm6b, Kmt5b, Ptchd1, Setd5, Trip12, Usp7 or Wac), One exception is Bckdk which is kinase that directly regulates ketone metabolism.
Perhaps the most dramatic finding was a remarkably consistent trend in forebrain cells lineages across multiple mouse models demonstrating, at E14.5, an increase in radial glia cells and corresponding decrease in cortical plate cells. Strong effects were observed in Cul3, HnrnpU,

Kdm6b, and Ptchd1 models and weak trends were observed in other models. If this finding is mostly robust, it suggests that delayed progression of the radial glia cell lineage is a "convergent" effect of multiple ASD genes.
Differential expression of genes was investigated in pseudobulk RNA from specific cell types. With regard to the total number of DEGs, the strongest effects were observed in P4 layer II/IV neurons. This pattern was surprisingly similar across all mutants. Several synaptic proteins, ion channels and cytoskeletal proteins were found to be differentially expressed across which included several genes implicated in ASD. This type of evidence for convergence has been seen before in other mouse models of ASD genes, but this the first evidence pointing to relatively small very specific set of genes that are consistently dysregulated in both period and cell type across many mutants. There were also many DEGs that were unique to each mutant. The overall interpretation is basically "we see commonalities and we see differences", which is a bit unsatisfying. Overall effects of genes on expression were stronger in females. A subset of genes that showed female biased differential expression were identified, and subset of these were observed in multiple mouse models. Sex biased DEGs were validated by bulk RNASEq of one line hnrnpU E16.5. Gene expression and chromatin accessibility were positively correlated for genes and gene sets.
I assert that the convergent findings are of high significance IF THEY ARE ROBUST. I do have some questions and concerns about the overall design and approach, that I think should be addressed to fully assess their credibility. Overall a more rigorous analytical approach to quantifying convergence and divergence of genes and pathways among differentially expressed genes is needed.

We thank the reviewer for the valuable feedback and recognition of our study concept and experimental approach.

1. Mouse cohort breeding. Each time point included 3 mice per genotype per sex, which were drawn from 3 different litters. Were matched littermate controls used for each mouse model? (This is mentioned for the bulk RNA-Seq but was not described for the scRNA samples). Or were all mouse models compared to a single control group of 3 per genotype per sex (which would generally not be littermates, would have been bred separately and would not be analyzed in parallel with all mouse models)? IF it is the latter, this would present some challenges. If controls were derived from separate litters than the mouse models, there is potential for there to be a few rare coding mutations in that line that can accumulate over a couple generations. In addition, genes or pathways that form outliers in a single small group (N = 3 male, 3 female) of controls, would appear as convergent cell type effects and convergent DEGs relative to most of the mouse models. It would be difficult to distinguish idiosyncratic characteristics of a small control group from "convergent" effects of ASD genes. Again, this would not be a major concern if the controls were drawn from littermates of several different mouse models… and they were not bred and analyzed separately.

Response: We appreciate the reviewer's comments on our breeding strategy and the importance of appropriate controls. In alignment with 3R principles and given the large number of animals required across multiple mutant lines, which would have made this study unaffordable, we did not test every mutant line against its own wild-type littermates. However, we designed our experiments to maximize comparability while minimizing genetic drift and line-specific idiosyncrasies.

Specifically, all our mutant lines are maintained in a C57BL/6J (BL6) background. To maintain this background, colonies are continuously refreshed by mating mutants with BL6 animals drawn

from a continuously refreshed central BL6 colony. All the sequenced mutant animals were generated by mating a BL6 female from the continuously refreshed central BL6 colony with a mutant male maintained on the same BL6 background (e.g., BL6 female × *Ash1l*$^{+/-}$ male = litter sequenced). The only exceptions were homozygous ***Bckdk*** mice whose heterozygous breeders were regularly refreshed with the same central BL6 colony. Control animals were BL6×BL6 offspring sourced from the same central colony (Revision Fig. R1.1). Dams used for mutant and control litters were drawn from the same central-colony cohorts and often littermates; at minimum, they were not separated by generations of inbreeding. For each time point and genotype (including controls), we analyzed at least 3 independent litters (at least *n*=3/sex, *n*=6 per genotype).

[Redaction]
Figure Includes
Third Party Material

Revision Fig. R1.1: Breeding scheme employed for the generation of ASD mutant mouse models
All ASD mutants were generated by mating C57BL/6J (BL6) females from a refreshed central colony with mutant males. The *Bckdk* line was studied homozygously, with heterozygous breeders periodically outcrossed to the same colony. Controls were BL6 × BL6 offspring from this colony.

This ensures that:

1. **Minimized Rare Coding Mutations:** Using a single, refreshed colony for both mutants and controls limits the accumulation of rare coding variants and reduces the chance that idiosyncratic control features are misinterpreted as convergence. Since both our mutant lines and our control animals are bred with the same central BL6 colony, any rare mutation would either be shared in both groups or rapidly diluted out by the continuous refreshment of the BL6 colony.
2. **Outlier Genes/Pathways:** Although the sample size of *n*=3 per sex and genotype is indeed small, our controls are drawn from the same foundational BL6 colony to which our mutant lines are continuously outcrossed. This significantly reduces the likelihood that any observed 'convergent' effects are merely idiosyncratic characteristics of a single small control group. Instead, any commonalities observed across different mutant models, when compared to this standardized BL6 control, are more likely to represent true convergent effects related to the respective gene perturbations, rather than artifacts of a divergent control group. Furthermore, most of our observations are based on *n*=6 rather than 3.
3. **Ensuring Genetic Homogeneity:** We believe this design represents the cleanest approach for comparing multiple distinct genetic models. By constantly refreshing the BL6 background for both our mutants and controls and by employing a central BL6 colony, we minimize the accumulation of line-specific genetic variations that could confound comparisons between different mutant strains.
4. **Balanced processing:** We would also like to point out that dissections and library preparations were carried out in parallel for both mutant and wild-type samples. Multiple litters were processed per day, with genotypes (including controls) prepared side by side. Additionally, the controls were distributed across several multiplexes and processed alongside the different ASD mutant strains to ensure balanced experimental handling.

5. **Control for outliers**: A leave-one-out cross-validation strategy, which systematically removed one of the wild-type mice from the DESeq2 comparison, was employed to investigate whether any of the wild-type mice were strong outliers.

To ensure that our designed breeding scheme is clearly understood, we have revised the Materials & Methods section "*Sample preparation and Nuclei isolation"*, to specify the control line used and detail the standardized breeding approach applied.

Nevertheless, to address the concern of the controls used, we generated a second snRNA-seq dataset with a subset of mutants (*HnrnpU* at E14.5; *Bckdk* and *HnrnpU* at P4), this time including wild-type littermate controls. **The breeding scheme and the central colony were identical to the initial study** (mutant male x BL6 female from our central colony). Differences were the inclusion of littermate wild-type controls and the use of an snRNA-seq-only library preparation kit (10X Genomics; 3' Gene Expression) instead of the Multiome kit used initially. For simplicity, we will refer to the **initial dataset** as **Dataset A** and the **new dataset** as **Dataset B**.

We harmonized Datasets A and B by integrating them into a shared latent space using Harmony and applied a conservative batch correction. We then analyzed Dataset B using the same DEG pipeline used for Dataset A, and compared the $\log_2$FC values of the DEGs from Dataset A with those measured in Dataset B. Overall, the datasets showed a strong concordance, with correlations up to r = 0.90 (Revision Fig. R1.2a,b).

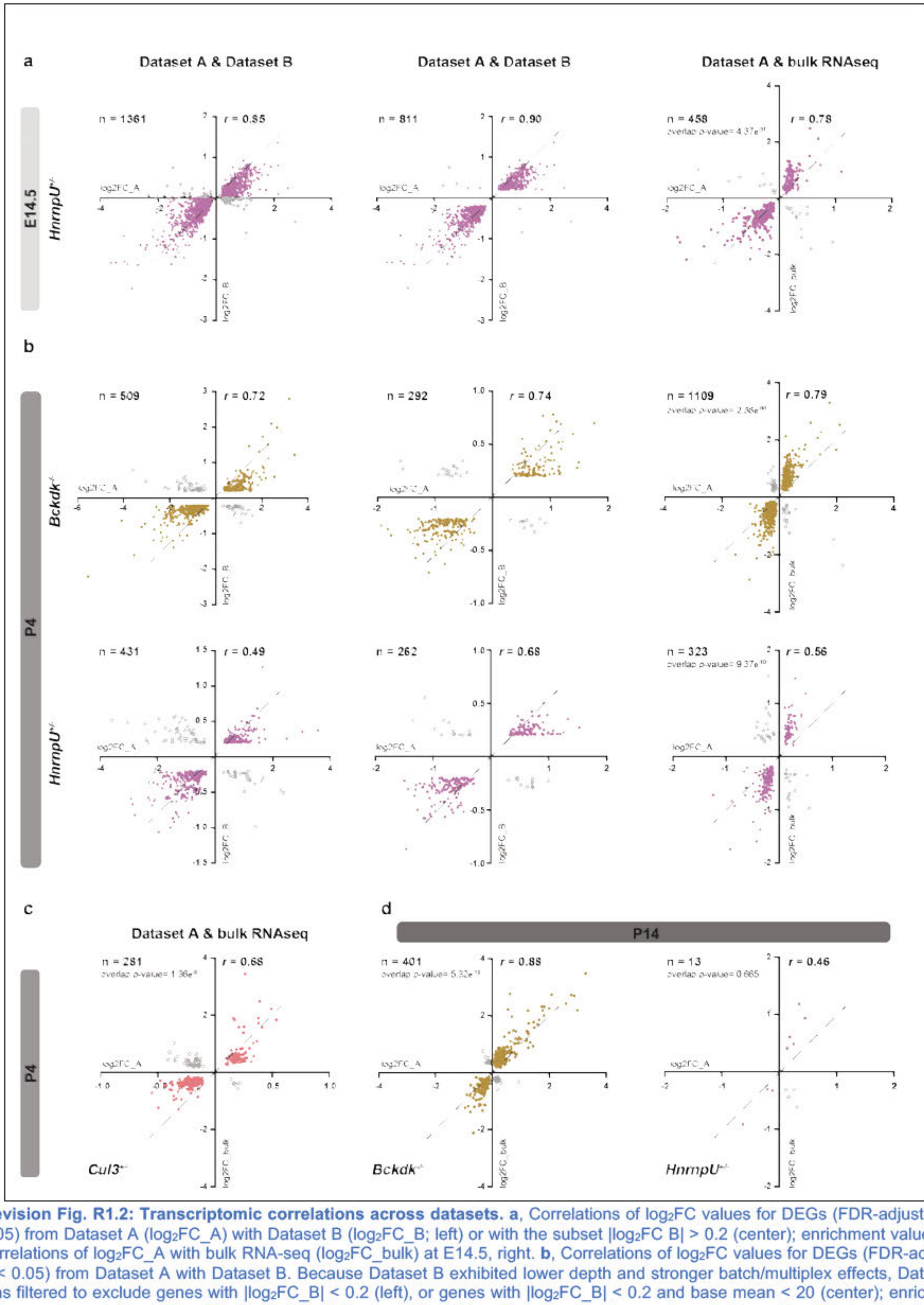

Revision Fig. R1.2: Transcriptomic correlations across datasets. a, Correlations of log_2FC values for DEGs (FDR-adjusted $p < 0.05$) from Dataset A (log_2FC_A) with Dataset B (log_2FC_B; left) or with the subset $|log_2FC\ B| > 0.2$ (center); enrichment values and correlations of log_2FC_A with bulk RNA-seq (log_2FC_bulk) at E14.5, right. **b**, Correlations of log_2FC values for DEGs (FDR-adjusted $p < 0.05$) from Dataset A with Dataset B. Because Dataset B exhibited lower depth and stronger batch/multiplex effects, Dataset B was filtered to exclude genes with $|log_2FC\_B| < 0.2$ (left), or genes with $|log_2FC\_B| < 0.2$ and base mean < 20 (center); enrichment values and correlations of log_2FC_A with bulk RNA-seq at P4, right. **c**, Enrichment values and correlations of log_2FC_A with bulk RNA-seq for *Cul3* at P4. **d**, Correlations of log_2FC_A with bulk RNA-seq at P14. Spearman correlation was applied when normality was

violated; otherwise, Pearson's r was used. Identity line is shown for reference. Overlap *p*-value obtained from one-tailed Fisher's exact test (enrichment). Animals used for analyses: Dataset A; *n (E14.5)*= 6 (*HnrnpU*$^{+/-}$, colony-matched controls), *n (P4)*= 6 (*Bckdk*$^{-/-}$, *HnrnpU*$^{+/-}$, colony-matched controls), Dataset B: *n (E14.5)*= 6 (*HnrnpU*$^{+/+}$, *HnrnpU*$^{+/-}$), *n (P4)*= 6 (*Bckdk*$^{+/+}$, *Bckdk*$^{-/-}$, *HnrnpU*$^{+/+}$, *HnrnpU*$^{+/-}$), bulk RNA-seq: *n (E14.5)*= 6 (*HnrnpU*$^{+/+}$, colony-matched controls), *n (P4)*= 6 (*Bckdk*$^{-/-}$, *Cul3*$^{+/-}$, *HnrnpU*$^{+/-}$, colony-matched controls), *n (P14)*= 6 (*Bckdk*$^{-/-}$, *Cul3*$^{+/-}$, *HnrnpU*$^{+/-}$, colony-matched controls).

We also compared Datasets A and B at the level of GSEA analysis. Pre-ranked GSEA using Dataset A as reference and B as query showed significant, concordant enrichment for most conditions (Revision Fig. R1.3).
Thus, despite using a different library kit, a ~2-year interval between experiments, and different controls and mutant animals, we found very good agreement between the two datasets, supporting the robustness of Dataset A, even though Dataset A used non-littermate but colony-matched controls.

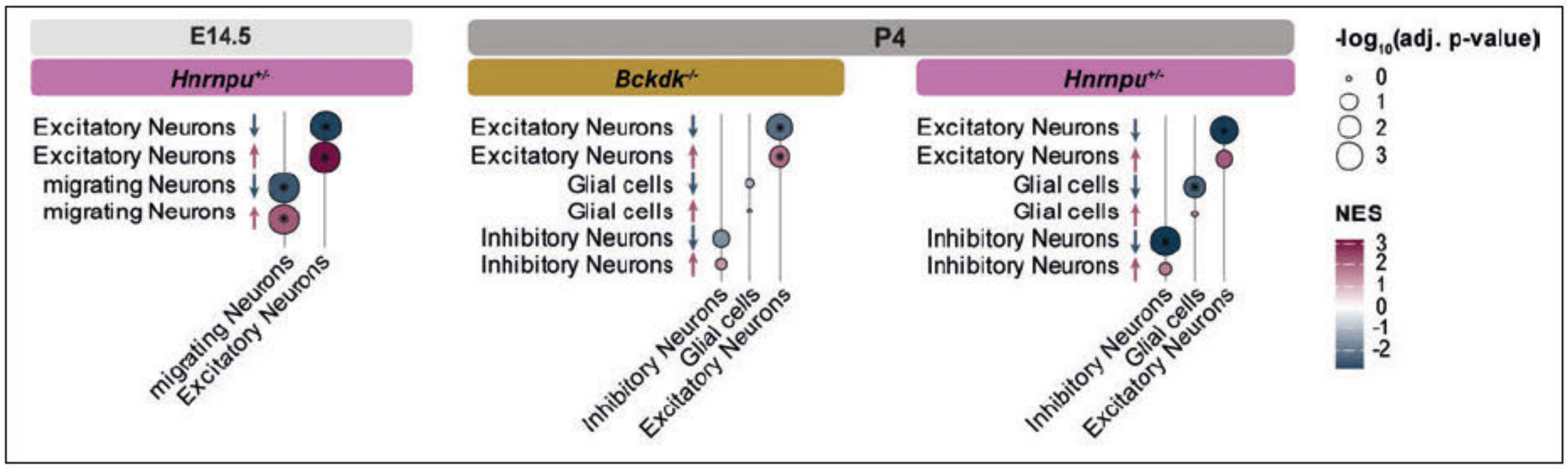

Revision Fig. R1.3: Gene set enrichment validation of Dataset B.
An enrichment database was generated from the DEG lists of Dataset A. Dataset B differential expression results were then subjected to pre-ranked GSEA against this database. Dataset B conditions show positive enrichment (NES > 0) for corresponding upregulated terms and negative enrichment (NES < 0) for downregulated terms.

We would like to note that, despite the high overall concordance, Dataset B showed a weaker signal relative to Dataset A. This included a general reduction in effect sizes (i.e., $\log_2FC$) (Revision Fig. R1.2a,b) and the failure of several positive controls, previously identified in independent mutant-littermate studies (e.g., Novarino et al. 2012) and confirmed in Dataset A, to pass significance thresholds.

A closer inspection indicated that the reduced statistical power was driven by technical rather than biological factors. Specifically, we assessed potential confounding effects by testing associations between metadata variables and the first ten principal components (PCs). Dataset B, particularly at P4, showed a strong batch/multiplex effect (Revision Fig. R1.4), accounting for approximately 10% of the variation captured by PC2. The leading PCs were mainly driven by expected factors such as cell type, condition, read counts, and the number of detected genes. However, the batch effect was markedly more pronounced in Dataset B, whereas it was minimal in Dataset A, indicating a substantial technical confounding influence.

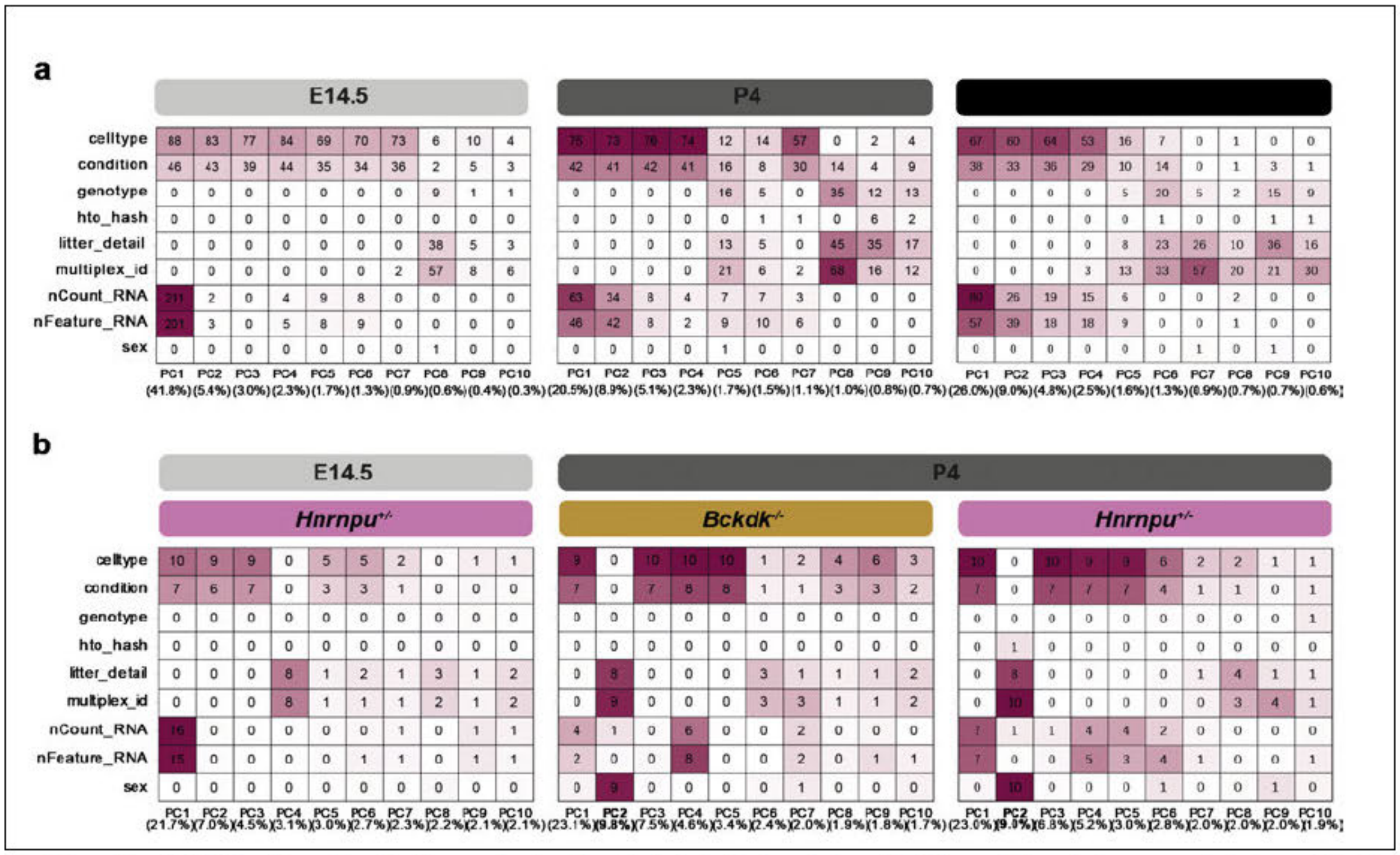

Revision Fig. R1.4: Confounding factor analysis of metadata in Dataset A and B. Associations between metadata and the first ten PCs from PCA in Dataset A (**a**) and Dataset B (**b**). *P*-values were adjusted using the Benjamini-Hochberg method. Heatmaps show -log$_{10}$ adjusted p-values with hierarchically clustered metadata. PCs in Dataset A are mainly driven by expected factors, whereas Dataset B shows a pronounced batch effect.

Moreover, Dataset B showed lower reads per cell and fewer detected genes (i.e., reduced sequencing depth), as well as 4-8% fewer reads mapping to protein-coding transcripts compared with Dataset A (Revision Fig. R1.5). Nevertheless, we cannot exclude that complex biological factors may also contribute to the observed reduction in effect sizes.

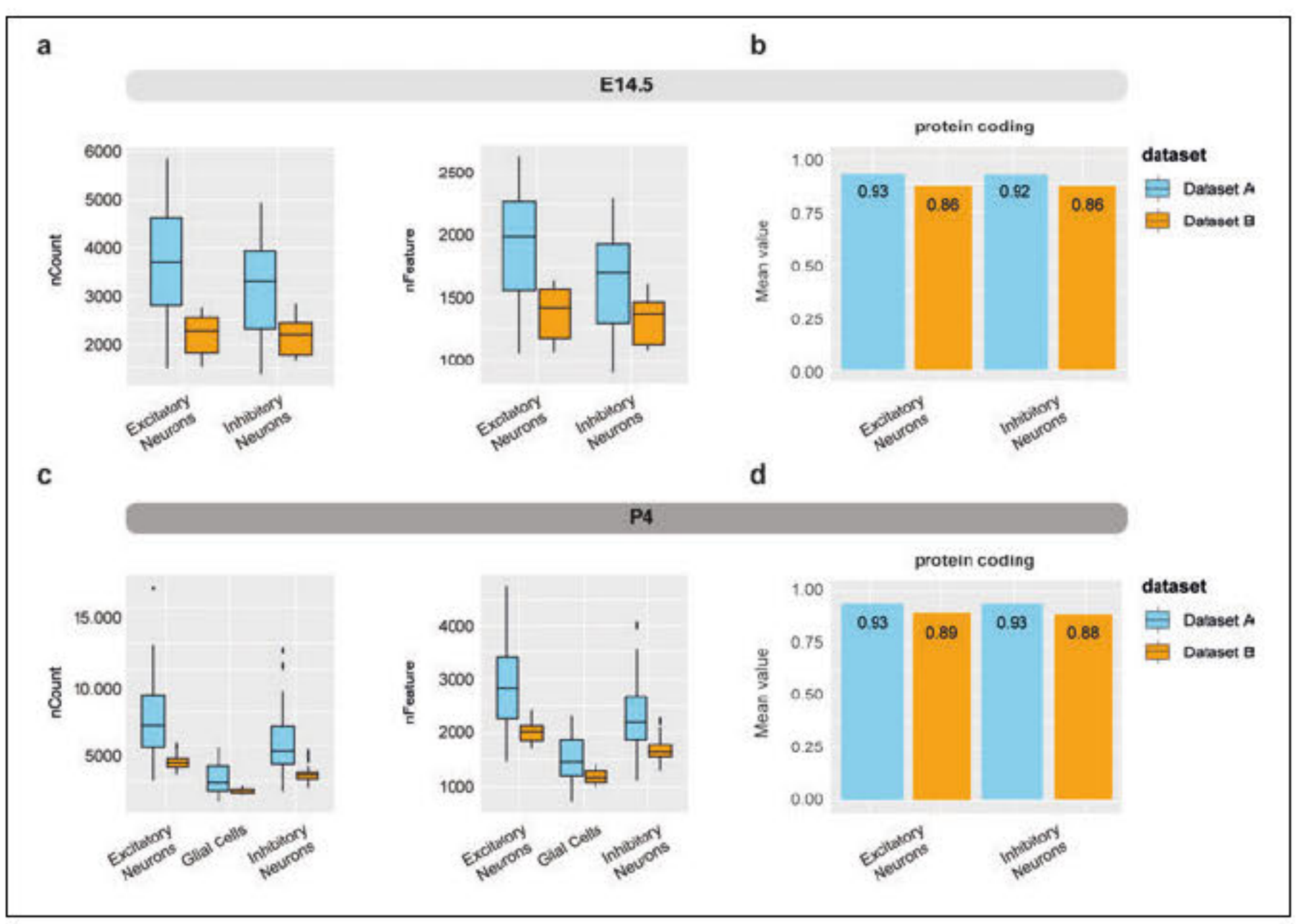

Revision Fig. R1.5: Overview of sequencing quality metrics of sequencing datasets

a, **c**, Quality metrics showing the median number of sequencing depth (left) and genes detected (right) in Dataset A (blue) and Dataset B (orange) across cell types and time points. Center lines represent medians, the box limits show the 25-75% interquartile range (IQR), whiskers extend to 1.5 the range of the IQR. **b**, **d**, Barplot showing reads mapped to protein-coding RNAs in Dataset A and Dataset B across time points.

Finally, to further evaluate the reproducibility of Dataset A, we performed bulk RNA-seq for selected genotypes and time points, using new colony-matched BL6 control and mutant samples. We found a highly significant overlap between DEGs identified in Dataset A and bulk RNA-seq. In addition, the shared DEGs showed comparable effects (log_2FC) (Revision Fig. R1.2a, b (right), c, d, Extended Data Fig. 4i in the revised manuscript).

Altogether, our analyses indicate that the results are robust across control types and methods. Nevertheless, all functional and validation experiments (see later points) were performed with line-matched wild-type littermates alongside colony-matched BL6 controls, following the same breeding scheme and central BL6 colony as described (Revision Fig. R1.1).

2. Not sure what the guidelines are at nature but it's generally a good idea to include the numbers of wt and control animals per group in the figure legends.

Response: We apologize for this, as described in the text, we did not initially include the exact *n* numbers for each sequencing. However, the reviewer is right and these numbers should be clearly stated also in the figure legends. We have amended this in the revised version of the manuscript.

3. Is the reduction in CP cells and increase in CP cells consistent with other published mouse models? Or Human models? Some studies do show an effect that contrasts with this (but are zeroing in on the same process). For instance human brain organoid models at mid-to-late fetal period of 16p11.2 deletion show reduced numbers of neural progenitors and increased numbers of mature neurons (PMID: 34433918, PMID: 36826401).

Response: The reviewer is correct. Several studies have reported alterations in cortical plate (CP) and radial glia (RG) cell numbers in ASD-related models, with both increases and decreases observed across different systems. Our results are most consistent with transient shifts, reflecting delays or accelerations in developmental trajectories rather than lasting changes in final cell numbers. This view fits with the observation that just a subset of patients with ASD show changes in head/brain size and with the literature on the specific genes examined in this study. According to the most recent SFARI gene database (queried version Q3 2025), except for *Bckdk*, none of the genes studied here have been specifically associated with either microcephaly or macrocephaly. Our review of the current literature revealed no evidence that mutations in *ASH1L, CUL3, HNRNPU, SETD5, TRIP12, USP7,* or *WAC* lead to altered brain size in patients. In contrast, patients and mice with *Bckdk* mutations develop postnatal microcephaly (Novarino et al., 2012 and unpublished data), in agreement with our findings. Approximately 25% of patients with *KDM6B* mutations present with macrocephaly, which could be compatible with our observations[1]. Finally, about 60% of patients with *KMT5B* mutations show macrocephaly; however, this phenotype is thought not to result from altered proliferation, as Ki67 staining in mutant mice did not reveal changes in proliferative activity[2]. Moreover, it remains unclear whether the enlargement observed in *KMT5B* mutants reflects a cortical or subcortical phenotype[2]. Nevertheless, we would also like to acknowledge a limitation of our analysis. Specifically, if a mutation affects the absolute number of all cell types uniformly, we would not be able to capture this with snRNAseq, which only provides information on relative cell-type proportions. We added this information in the limitation paragraph.

Furthermore, we validated the observed alterations in proliferating RG cell numbers in one selected mutant (i.e., *HnrnpU*), using two complementary approaches. First, we analyzed Dataset B and observed the same increase in RG cells as reported in Fig. 1e of the manuscript (Revision Fig. R1.6), not only validating the results but also confirming that these conclusions were not influenced by the controls used for Dataset A.

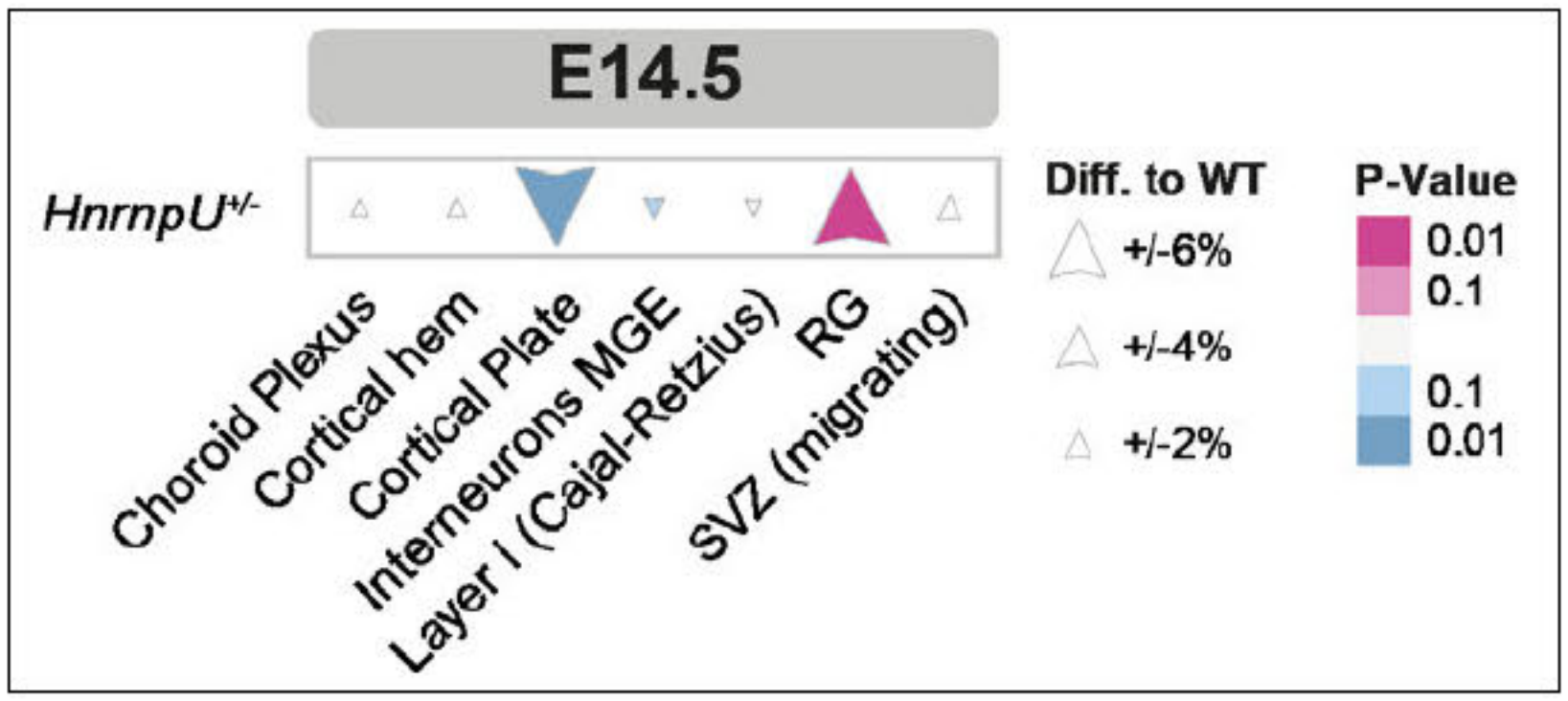

Revision Fig. R1.6: Cell type abundance changes in *HnrnpU* mutants. Changes in cell type abundances in *HnrnpU* mutants at E14.5. Arrows represent the relative difference in percentage compared to wild-type littermate controls. Nominal *p*-values are shown. Animals used for analyses: *n (E14.5)*= 6 (*HnrnpU*$^{+/+}$, *HnrnpU*$^{+/-}$).

Second, we employed an EdU-labeling strategy as a method orthogonal to sequencing. Precisely, pregnant females were injected with EdU at E14.5 (14 days post coitum), and brains from *HnrnpU*$^{+/-}$ mutants and their wild-type littermates were collected 6 hours later. To closely parallel the quantification performed by snRNA-seq, we combined EdU labeling with CUBIC tissue clearing and quantified labeled cells within the entire cortex. Consistent with the snRNAseq results, *HnrnpU*$^{+/-}$ mutants showed increased numbers of EdU-positive cells (Extended Data Fig. 3e), indicative of an expanded pool of proliferative RG cells at E14.5.

4. Not sure how to interpret the convergence and divergence of DEGs between mutants. The analysis of molecular pathways seems view all DEGs that overlap between mouse models as "convergence" or "independent validation of the DEGs" and all DEGs that do not overlap were viewed as "divergence". Without a more rigorous analysis of the molecular convergence of these 11 mutants this kind of overlap is hard to interpret. There are statistical methods for determining if overlap between 2, 3, 4, 5··· sets of DEGs is significant. See for example the R package SuperExactTest. In addition, There are computational methods for quantifying convergence of gene sets at the network level by network colocalization (PMID: 36653526) that may be more powerful than quantifying overlaps of specific genes. It feels like the authors are basically giving a broad description of the observed "convergence" but without a rigorous statistical analysis the reader is left to sort of draw their own conclusions.

Response: We thank the reviewer for their suggestions, particularly for recommending a network-level analysis. We believe that this analysis is more robust than simple DEGs overlaps. By taking into account all gene expression changes jointly within a network context, this analysis also minimizes issues related to false-positive DEGs. We have now employed both suggested approaches; below, we summarize the results.

We have applied the SuperExactTest and found that in excitatory neurons at P4, all pairwise overlaps across mutants were significant (SuperExactTest 45/45 pairs, FDR < 0.05), with a median fold-enrichment (FE) of 4.46. The strongest overlaps (e.g., *Kdm6b* with *Ash1l*) reached FE values above 10. Significant overlaps were also observed among larger sets of mutants and in inhibitory neurons. At E14.5 and P14, overlaps remained significant in many cases (e.g., P14: 6/28 pairs, FDR < 0.05) but were generally weaker (median FE = 0.82).

However, given the dependencies introduced by using a single central control group, we have decided to perform a separate statistical analysis, complementing the suggested statistical testing. Specifically, each mutant pair was compared against distinct sets of control samples. Control samples (including both male and female samples) were randomly divided into two disjoint groups. For each mutant line pair, one mutant line was compared to the first control group and the other to the second. Assigning distinct, non-overlapping control groups to each mutant line prevented shared variance and thus maintained statistical independence between the two comparisons. For each mutant line pair, differential expression analysis was performed using DESeq2.
Overlap between each mutant pair was assessed by comparing the top 300 most differentially expressed genes (ranked by *p*-value), and the observed-to-expected ratio and statistical significance of the overlap were determined using Fisher's exact test. Altogether, these results confirm that the overlaps we observed represent significant, convergent, non-random overlaps of transcriptional responses across genotypes (Extended Data Fig. 4f and Methods).

We next employed network colocalization (NetColoc) analyses, following the method suggested by the reviewer. First, we constructed a protein-protein interaction (PPI) network from publicly available datasets, comprising 18,921 proteins and 417,767 undirected interactions (see Methods for details). Within this network, we identified clusters of functionally related proteins by clustering shared genes based on averaged random walks. Each cluster was subsequently assessed for functional enrichment using GO annotations. For each genotype, we quantified the effect size (i.e., magnitude) and significance of expression changes across the identified clusters, generating a network-wide map of how each mutation influences the activity of biological processes within excitatory and inhibitory neuronal lineages (Fig. 2c, Extended Data Fig. 9a).
We then compared the resulting network localization profiles across genotypes and developmental time points to assess the similarity of affected gene systems across genotypes. This analysis assesses how similarly the top-ranked genes from each condition are positioned within the functional network, providing an independent measure of convergence across shared gene systems. (Fig. 2d, Extended Data Fig. 9b). This analysis showed that both the network structure and similarity scores are primarily shaped by developmental stage. Within each developmental stage, the similarity between mutants was significantly higher than expected by chance. At the latest stage (P14), however, mutants were less similar to one another than at earlier stages, particularly in excitatory neurons, indicating divergence of genotype-associated transcriptional programs, superimposed on stage-specific convergence.

We believe that these additions considerably strengthen the manuscript, and we thank the reviewer for the suggestions. We have changed figures and text accordingly.

5. Is there a plausible explanation for why all mutants show a nearly identical pattern of strong differential expression of genes in P4 excitatory neurons and comparatively weaker effects at E14.5. Why do you suppose phenotypic effects were more concentrated in E14.5 but effects on gene expression were not?

Response: We thank the reviewer for raising this point. We have considered it carefully. Based on the results presented here and previous studies of the group[3], we interpret the phenotype observed at E14.5 as a consequence of earlier molecular perturbations that transiently delay, or shift, development. At E14.5, however, when examined at single-cell resolution, we do not detect strong cell-intrinsic effects, indicating that these cell types are molecularly similar to each other. Instead, we propose that this delay impacts circuit assembly, consistent with the robust molecular changes observed at P4.

6. And then in comparing all strains to wild type finds exactly 2 genes with very large differential expression. Rsrp1 galnt17. Is this pseudobulk of all samples vs wild type? These 2 genes stand out so much, they look almost like they were CRISPR'd. Were these significant DEGs in any of the individual mutants? Or only in the combined data? Can you rule out (1) off target effects in these genes AND (2) coding mutations that accumulated in the BL6J line before breeding the controls for this experiment.

Response: We would like to clarify some aspects. First, not all lines included in this study were generated using CRISPR/ Cas9. Specifically, *Ash1l*$^{+/-}$*, Kdm6b*$^{+/-}$*, Kmt5b*$^{+/-}$*, Trip12*$^{+/-}$*, Usp7*$^{+/-}$ and *Wac*$^{+/-}$ mutant lines were generated using CRISPR/ Cas9 (as described in the Material & Methods section (*"Generation of constitutive heterozygous mouse lines"*), whereas *Bckdk*$^{-/-}$, *Cul3*$^{+/-}$, *HnrnpU*$^{+/-}$, *Ptchd1*$^{-/y}$, and *Setd5*$^{+/-}$ mutant animals were generated through targeted homologous recombination. Second, all CRISPR-derived lines were evaluated for potential off-target effects. Third, the cell-type specificity of the observed phenotype argues against a CRISPR off-target artifact, as the change is restricted to a small subset of cells.
The differences in *Rsrp1* and *Galn17* were significant at the nominal *p*-value in all genotypes except for Ptchd1 (*p-value* = 0.058). In *HnrnpU* mutants, both genes were significant at the adjusted *p*-value. Finally, we observed a downregulation of *Rsrp1* and *Galn17* in Dataset B in *HnrnpU* mutants, as compared to their littermate controls (*p*-values = 0.027 and 0.065, respectively).

7. Cell type pseudobulk of all mutants vs wild type. Fig 2A. The cell types that have the greatest number of DEGs are consistently excitatory neurons at P4 and to a lesser extent inhibitory neurons. Are the genes that are being modeled in these mutants expressed predominantly in P4?

Response: No, they are not; we have revised Extended Data Fig. 1b, c to illustrate the expression patterns of our selected genes across developmental stages and cell types, demonstrating that the genes analyzed are ubiquitously expressed across time and cell types. One possible explanation for this observation is that, although we do not detect a direct relationship between the number of cells sequenced and the number of differentially expressed genes (DEGs), sequencing depth may still have an effect. To address this directly, we performed a downsampling-based differential expression analysis aimed at balancing statistical power, which we consider more suitable for comparing relative numbers of DEGs across cell types. This analysis shows that, in many cases, neurons are the most affected cell type, with excitatory and inhibitory neurons similarly impacted. However, there are instances in which glial cells are affected to a greater extent. Accordingly, we have revised the text and replaced panel D of Extended Data Fig. 3 (Extended Data Fig. 4c in the revised manuscript), which showed a non-linear relationship between the number of sequenced cells and DEGs, with the downsampling analysis.

8. "At P4, mutants displayed a marked convergence on pathways related to synaptic and ion channel functions (Fig. 2d, bottom).The blue color of all the nodes in this network in Fig 2d appear to correspond to one gene in the legend (Wac). Were the nodes in this network supposed to be color-coded by gene? From this figure, I can't infer anything about convergence. Extended data figure 4b does have color coded circles around the clusters in the enrichment map and the gray clusters are shared by a majority of mutants. But the colors in the figure are hard to match to the legend.

Response: We apologize for the confusion. The reviewer is correct that the figure could be misleading. The node colors were not coded by genotype; the accompanying list of genotypes

was provided only to indicate which genotypes were used to generate the maps. We have now completely revised Fig. 2, replacing it with the analysis suggested by the reviewer in Comment 4 and have retained Extended Data Fig. 4 (Extended Data Fig. 5 in the revised manuscript).

9. The enrichment maps in Figure 2d do not do a great job of illustrating the relationship of the various mouse models to pathways. Extended data figure 5 does a better job of illustrating the direction of the effects and linking them to specific mouse models. This shows downregulation of synaptic transmission and upregulation of RNA processing and translation as a convergent mechanism. But the results are still fragmented into separate plots for each cell type and developmental period. Wouldn't it be possible to define a variety of gene sets that correspond to the major categories in Figure 2d, and then to create heatmap illustration that combines the normalized enrichment scores of the gene sets across all mouse models, cell types and periods? I would anticipate that this would highlight various points of convergence and divergence between mouse models in a more unified way, and the reader would not need to look for meaningful similarities and differences across multiple figure panels. Ideally, this kind of heatmap would NOT have missing values for nonsignificant enrichments. Significance could be indicated by varying some other feature of the heatmap (size, shape, etc.)

Response: We have changed Fig. 2, emphasizing the relationships across mutant lines condensed in one figure, as suggested by the reviewer, using the output of the Network colocalization analysis.

10. "Despite some commonalities, each mutant showed a unique combination of affected processes, suggesting that every mutation leads to a distinct synaptic state (Fig. 3a')" how does one quantify the uniqueness of the plots in Fig 3a'? If the results were NOT unique for each mutant, what exactly would that look like? Even replicate experiments of the same mouse model are not going to produce identical results. There would be differences. What does the null expectation look like? What is it about the single gene heatmaps in Fig 3d,e that make them "unique" and how are they are more unique than the "convergent" patterns for ion channels in Fig 2b,c?

Response: We thank the reviewer for this comment. We agree that defining "uniqueness" in a statistical sense is difficult. The former Fig. 3a' illustrated diversity or heterogeneity rather than strict uniqueness. In Fig. 3d,e, we compared each mutant against all other mutants collectively, thereby highlighting changes that are not shared with all the other mutants. Yet, this does not imply uniqueness, but rather DEGs that are not shared across most mutants. We have therefore revised the text accordingly to better reflect this distinction, and we have deemphasized and moved Fig. 3a to the Extended Fig. 8. Overall, we have revised the text throughout to adopt a more conservative phrasing and no longer refer to the patterns as "unique".

Referee #2 (Remarks to the Author):

In this manuscript, "Developmental dynamics of the cortical cellular and molecular landscapes in autism spectrum disorder", Schwarz and colleagues aim to understand how so many genes functioning in different molecular mechanisms lead to somewhat similar phenotypes in autism spectrum disorder (ASD). To do this, they performed single nucleus multiome sequencing coupling gene expression profiles and chromatin landscape from the cortex (and in some cases cerebellum) of 11 different mouse models of ASD at three different timepoints in development. They found that the studied mutants have a delay in the radial glia lineage progression, that gene expression changes were most prominent at postnatal day 4, affecting essential synaptic and neurotransmission-related pathways, that the chromatin landscape was most impacted at embryonic day E14.5, that there were sex-specific differences, and that the studied ASD genes converge on essential neurodevelopmental pathways, while also affecting pathways unique to each gene.

Overall, the study is extensive and is the first to look at 11 mouse models of autism simultaneously, while aiming to find pathways of convergence. Strengths of the study include addressing a central question in ASD research about the extent of molecular and cellular convergence of distinct mutations, and employing systematic and unbiased analysis of multiple ASD mutations simultaneously. However, several major and minor points need to be addressed to increase the rigor and impact of this work.

We thank the reviewer for the valuable feedback and recognition of our study concept and experimental approach.

Major points:

1. Our major concern is that none of the single nuclei multiomics conclusions are followed up by validation, including the major conclusion about a developmental delay affecting the radial glia lineage. This means that all the results and conclusions are entirely based on one experiment. Single nuclei results need to be confirmed with other methods: starting with the cell abundance results in the different mutants, the authors can confirm their data with quantitative single molecule fluorescence in situ hybridization or they can perform immunostaining for markers of the affected cell type at the different timepoints. The delay in radial glia lineage progression (representing some of the strongest evidence for convergence across ASD mutations) is a strong claim from single nuclei data alone and needs to be validated as well: tissue staining, more sophisticated proliferation analysis (e.g., with BrdU labeling), and/or flow cytometry showing increase in the RG1 cells in mutants, and experiments assessing proliferation of the RG in the mutant to confirm that the mutant RG remain in their proliferating RG1 state, are necessary. Similarly, one of the main conclusions of this paper is the disrupted synaptic function, the authors could validate those findings with electrophysiological recordings in the affected mouse models compared to controls.

Response: We agree that orthogonal validations can strengthen the manuscript. Accordingly, we performed new experiments, including additional RNA sequencing, *in situ* labeling, electrophysiology, and behavioral assays, for selected genotypes and time points, whereas extending every assay to every line would have required several years. Altogether, these additional experiments further strengthen our single-nucleus transcriptomic results, provide deeper insights into the functional consequences of the selected mutations, and illustrate the range of phenotypes they entail. The new experiments and results are outlined below.

Independent validation of cell counts by snRNA-seq: we performed an additional round of snRNA-seq (Dataset B) on *HnrnpU* mutants and their wild-type littermates. Consistent with the initial results, we again observed an increased number of radial glial cells (RG) and a decreased number of CP cells in mutants compared with wild-type controls (Revision Fig. R2.1). Molecular changes also aligned remarkably well with the initial dataset (Dataset A), with correlation coefficients up to $r = 0.9$ (Revision Fig. R2.2).

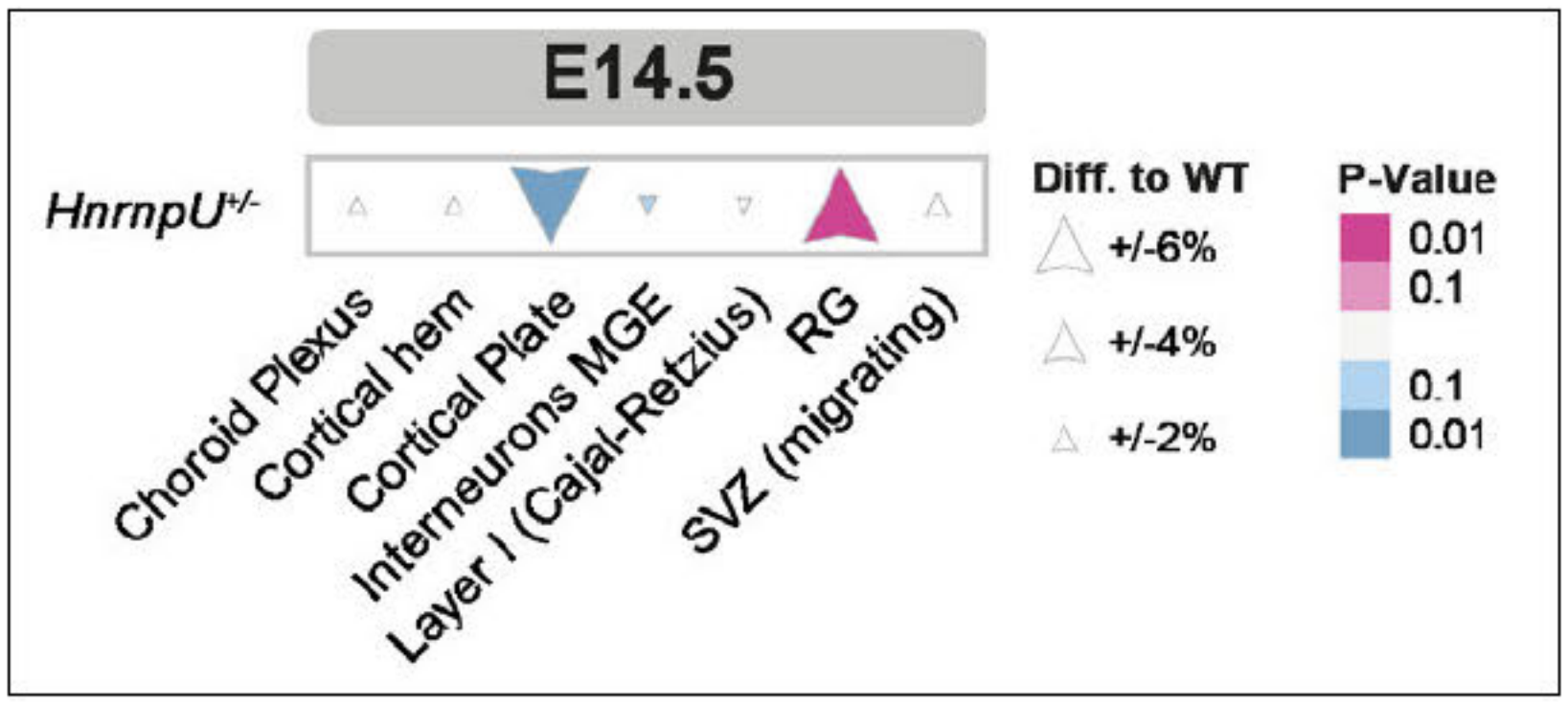

Revision Fig. R2.1: Cell type abundance changes in *HnrnpU* mutants. Changes in cell type abundances in *HnrnpU* mutants at E14.5. Arrows represent the relative difference in percentage compared to wild-type littermate controls. Nominal *p*-values are shown. Animals used for analyses: *n (E14.5)* = 6 (*HnrnpU*$^{+/+}$, *HnrnpU*$^{+/-}$).

EdU pulse-chase assay and RNA scope for cell counts: As an orthogonal validation of RG cell number changes, we performed EdU labeling in *HnrnpU*$^{+/-}$ animals and their littermate controls. Pregnant females were injected with EdU at E14.5 (14 days post coitum), and brains from *HnrnpU*$^{+/-}$ and wild-type embryos were collected 6 hours later. To parallel the quantification performed by snRNA-seq, we combined EdU labeling with CUBIC tissue clearing and quantified labeled cells within the entire cortex. Consistent with the snRNAseq results, *HnrnpU*$^{+/-}$ mutants showed increased numbers of EdU-positive cells (Extended Data Fig. 3e, Supplementary Video 1), indicating an expanded pool of proliferative RG cells at E14.5.

We have also performed **RNAScope analysis** for *Slc1a3* (immature astrocytes) and *Pdgfra* (OPC) to evaluate the number of immature astrocytes and OPCs in *HnrnpU* and *Kdm6b* mutant animals, alongside their wild-type littermates. The changes observed in the two selected mutants, *HnrnpU*$^{+/-}$ and *Kdm6b*$^{+/-}$, by *in situ* hybridization are comparable to those seen by snRNAseq (Extended Data Fig. 3d).

Bulk RNA sequencing: To validate the molecular results employing a different methodology, we additionally performed bulk RNA sequencing on selected mutants and time points. Bulk RNA sequencing results showed significant overlap in DEGs and concordant effect sizes with the initial snRNAseq dataset (Revision Fig. R2.2, Extended Data Fig. 4i).

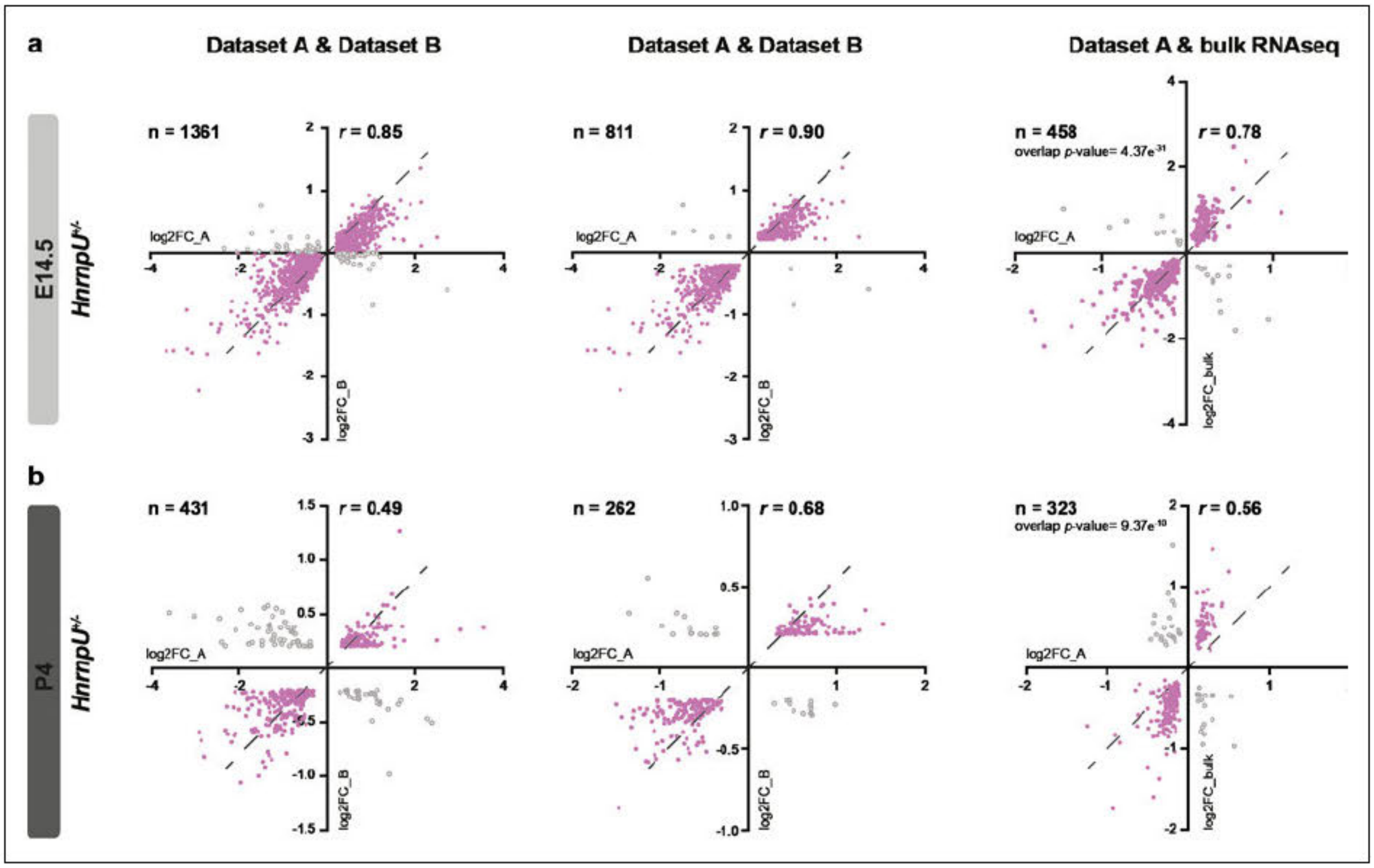

Revision Fig. R2.2: Transcriptomic correlations across datasets. a, Correlations of $\log_2$FC values for DEGs (FDR-adjusted $p < 0.05$) from Dataset A ($\log_2$FC_A) with Dataset B ($\log_2$FC_B; left) or with the subset $|\log_2\text{FC B}| > 0.2$ (center); enrichment values and correlations of $\log_2$FC_A with bulk RNA-seq ($\log_2$FC_bulk) at E14.5, right. **b**, Correlations of $\log_2$FC values for DEGs (FDR-adjusted $p < 0.05$) from Dataset A with Dataset B. Because Dataset B exhibited lower depth and stronger batch/multiplex effects, Dataset B was filtered to exclude genes with $|\log_2\text{FC\_B}| < 0.2$ (left), or genes with $|\log_2\text{FC\_B}| < 0.2$ and base mean < 20 (center); enrichment values and correlations of $\log_2$FC_A with RNA-seq at P4, right. Spearman correlation was applied when normality was violated; otherwise, Pearson's r was used. Identity line is shown for reference. Overlap *p*-value obtained from one-tailed Fisher's exact test (enrichment). Animals used for analyses: Dataset A; *n (E14.5)*= 6 (*HnrnpU*$^{+/-}$, colony-matched controls), *n (P4)*= 6 (*Bckdk*$^{-/-}$, *HnrnpU*$^{+/-}$, colony-matched controls), Dataset B: *n (E14.5)*= 6 (*HnrnpU*$^{+/+}$, *HnrnpU*$^{+/-}$), *n (P4)*= 6 (*Bckdk*$^{+/+}$, *Bckdk*$^{-/-}$, *HnrnpU*$^{+/+}$, *HnrnpU*$^{+/-}$), bulk RNA-seq: *n (E14.5)*= 6 (*HnrnpU*$^{+/+}$, colony-matched controls), *n (P4)*= 6 (*Bckdk*$^{-/-}$, *Cul3*$^{+/-}$, *HnrnpU*$^{+/-}$, colony-matched controls), *n (P14)*= 6 (*Bckdk*$^{-/-}$, *Cul3*$^{+/-}$, *HnrnpU*$^{+/-}$, colony-matched controls).

Electrophysiology: Given that one of our main findings was persistent transcriptional changes in genes encoding synaptic proteins and ion channels, we selected four mutant lines (*Bckdk*$^{-/-}$, *HnrnpU*$^{+/-}$, *Trip12*$^{+/-}$ and *Usp7*$^{+/-}$) and performed whole-cell patch-clamp recordings in Layer II/III pyramidal neurons at P7, the earliest time point near P4 at which robust synaptic activity can be measured (Fig. 3). The four lines were selected to span different degrees of transcriptional changes and were feasible to breed in sufficient numbers for electrophysiology experiments.

We quantified intrinsic membrane and action-potential (AP) properties, as well as recorded miniature EPSCs and IPSCs, in mutants and controls. In three of four ASD-risk models, we observed altered intrinsic excitability and/or input-output (F–I) relationships. *Trip12*$^{+/-}$ mutants were the exception, consistent with their comparatively small transcriptional divergence at P4. When we calculated Euclidean distances from wild-type samples in ion-channel expression space and compared them with distances in a multivariate space of intrinsic and AP features, the two measures showed strong alignment: genotypes with greater transcriptional deviation from controls also showed greater physiological divergence.
We further built a minimal conductance-based model resembling a P7 Layer II/III pyramidal neuron. Scaling ion channel conductances according to the direction and magnitude of expression

changes from our transcriptomic data reproduced the diversity of AP and F–I behaviors. This indicates that modest shifts in ion channel composition are predictive of the observed physiological variability (Fig. 3).

Miniature synaptic recordings mirrored the transcriptional signatures of synaptic dysfunction. We observed reduced mEPSC and/or mIPSC frequency in *Bckdk*$^{-/-}$, *Usp7*$^{+/-}$, and *Trip12*$^{+/-}$ mutant animals, consistent with the down-regulation of transcripts related to the presynaptic release machinery (e.g., *Rims1/2, Syn2, Dnm1l, Pacsin1*)[4–10]. Changes in mIPSC frequency were of similar magnitude, however, *Trip12* mutants showed a small but significant E/I frequency imbalance (Extended Data Fig. 10d). In *Bckdk* mutants, the reduced mEPSC amplitude was accompanied by a selective down-regulation of *Gria1* (*GluA1*), which is an established determinant of quantal amplitude[11], providing a direct molecular explanation for lower quantal size.
While some DEGs were shared, many were genotype-restricted, implying heterogeneous “synaptic compositions” and supporting a dose-like coupling: the greater the transcriptional deviation of synaptic- and ion channel-related genes, the larger the resulting intrinsic and synaptic physiological impact. One exception was the *HnrnpU* mutant, where broad transcriptomic changes were accompanied by mild miniature synaptic phenotypes. SynGO annotation of DEGs revealed downregulation of a core set of synaptic changes, but also displayed genotype-associated upregulation of genes such as *Ap3b2* (promoting vesicle biogenesis/ recycling)[12], *Tprg1l (Mover)* (modulation of release probability via Ca^{2+} sensitivity)[13], and *Diaph1* (stabilization of dendritic spines and presynaptic endocytic scaffolds)[14], which we interpret as potential compensatory mechanisms preserving transmission efficacy.
Overall, our electrophysiological results align with the snRNA-seq data at the pathway level. However, the net phenotype likely reflects multiple small, partially opposing effects, as well as post-transcriptional buffering, making one-to-one mapping between individual genes and phenotypes inherently difficult.

Finally, given the cerebellum’s central role in motor coordination, we examined the **motor behavior** of juvenile *Bckdk*$^{-/-}$ animals and compared it with that of *Kmt5b*$^{+/-}$ mice, which exhibit a single DEG in the cerebellum. Juvenile *Bckdk* mutant animals have motor impairments: they moved significantly less and had reduced stride and step lengths, even when normalized by body length. In addition, we observed a trend toward reduced motor coordination (Fig. 6e-g, Supplementary Videos 2, 3) and abnormal posture. In contrast, *Kmt5b* mutants showed no obvious motor abnormalities at the same age.

We have incorporated these new analyses into the revised manuscript.

2. For the ATAC portion of the analysis, mainly fig.4, the authors need to provide more insights and interpretations of their results: why are HnrnpU and Kdm6b mutants the ones with the significantly higher DARs than other genes? How do you explain Setd5 mutants not showing nearly as much DAR although it is a chromatin remodeler itself? What makes Kdm6b different than the other histone modifiers mutants studied in this paper that it shows that many DARs, whereas the others don’t? Given what we know about the chromatin landscape and gene expression changes, how do you explain DARs mostly present at E14.5 compared to DEG at P4? At the technical level, is the cell coverage sufficient for differential accessibility analysis – the authors note the use of nominal p-values due to the lower signal to noise and the TSS enrichment looks a bit lower than expected.

Response: In the revision, given the breadth of added data, the ATAC-seq analyses are positioned to contextualize the molecular findings rather than to serve as a primary focus. Nevertheless, we address the reviewer's questions in detail below.

HnrnpU encodes an RNA-binding protein with established roles in chromatin organization and nuclear architecture. Its loss has been associated with altered chromatin accessibility and gene regulation in multiple systems, which may explain the relatively high number of DARs observed. ***Kdm6b***, an H3K27me3 demethylase, directly modulates a broadly repressive chromatin state by removing H3K27me3 marks. Its disruption is therefore expected to cause widespread changes in accessibility, consistent with our findings.
In contrast, ***Setd5*** mutants show relatively few DARs. While Setd5 has been initially thought to be a histone methyltransferase, several studies have shown that it lacks the methyltransferase activity[3,15,16]. Setd5 mainly functions as a scaffold for chromatin remodeling complexes, and it regulates RNApol II occupancy and transcription of CNS-relevant genes. The results obtained here are supported by previous findings, which show that *Setd5* mutations impact neurodevelopment very early on (before or around E9.5) and do not impair synaptic gene expression in the hippocampus under naïve conditions[3].

While we lack direct evidence as to why *HnrnpU* and *Kdm6b* mutants exhibit substantially more DARs than other histone modifiers, our observations for *Kdm6b* are consistent with independent, unpublished data from our lab. In experiments challenging synaptic plasticity, *Kdm6b* mutants consistently showed larger accessibility differences compared to, for example, *Ash1l* mutants. It is possible that other chromatin remodelers or histone modifiers included in this study require specific environmental or developmental challenges to reveal stronger chromatin-level effects. A ChIP-seq approach might provide more detailed mechanistic insights into these pathways. While the whole mechanistic aspect is beyond the scope of this manuscript, we would like to thank the reviewer for this comment, prompting us to add a sentence on this in the discussion.

Regarding the temporal pattern, the general decrease in DARs from E14.5 to later stages aligns with previous studies[17,18], suggesting that chromatin becomes progressively restricted as neural cells differentiate. Less-mature cells likely maintain a more permissive and dynamic chromatin landscape to allow transcriptional flexibility, whereas more differentiated cells restrict accessibility as they adopt specialized identities.

We agree that ensuring high data quality is essential. Our TSS enrichment scores and sequencing depth are comparable to publicly available benchmark datasets, such as the mouse brain dataset provided by 10x Genomics (e.g., as shown in the Signac vignette). This confirms that our data quality is well within the expected range for single-cell ATAC-seq experiments of this type. We opted to use nominal (uncorrected) *p*-values for our differential accessibility analysis for two specific, context-specific reasons:

1) Unlike comparisons between distinct cell types (e.g., neurons vs. glia) where accessibility differences are vast, we are comparing control and mutant conditions within the same developing system. Since the overall developmental processes remain largely intact, we therefore expect the biological signal to be genuine but subtle.
2) ATAC-seq data is inherently sparser and noisier than RNA-seq data because accessibility is measured on a maximum of two DNA copies per cell, compared to potentially hundreds of RNA transcripts.

Given these factors, applying a stringent FDR correction would likely eliminate many true positives, masking the subtle but biologically relevant changes we aim to uncover. Our approach is thus a deliberate choice to balance sensitivity and specificity tailored to this particular biological question.

3. For the sex-specific effect, the data are also lacking proper and deeper interpretation. Given that the mutations studied are rare, and that they affect both males and females, the observed results in this paper are interesting and very informative to understand the sex differences, but they don’t necessarily help explain the sex bias seen in ASD diagnosis. The data should be interpreted as differences in males and females with mutations, not as explaining the sex bias. The authors should also reflect more on the larger effect size seen in females and what it could mean for females affected by these mutations.

Response: We thank the reviewer for the comment and agree that this is an important point. We have therefore extended the discussion on the perspective of this data. Nevertheless, we would like to point out that this is a first observation and that we will need an extensive analysis to understand the bases and the effects of such differences. Specifically, we are now following up on these observations to study chromosomal vs. hormonal effects. Alternatively, these findings could indicate that specific molecular or cellular pathways are differentially buffered or regulated between sexes.

Other points

1. I appreciate the pseudobulk analysis for differential expression. However, the text states that “Given the nature of the pseudo-bulk analysis, the number of sequenced cells per cluster was not expected to influence these findings.” I wonder if this is true, because the Extended data fig 2a-c heatmaps look by eye to show more divergence from median for the lower frequency cell types. At a statistical level, wouldn’t sequencing more cells from a particular cell type and genotype increase the precision of these measures compared with low cell count clusters? I would at least be careful of the above statement.

Response: We thank the reviewer for this comment. Although we did not detect a direct relationship between the number of sequenced cells and the number of DEGs, we acknowledge that sequencing depth could still have an effect. To account for this potential bias and to balance statistical power across cell types, we performed a downsampling-based differential expression analysis. This analysis is better suited for comparing relative DEG numbers between cell types. The results show that, in many cases, neurons are the most affected population, with excitatory and inhibitory neurons similarly impacted. However, in some instances, glial cells show a stronger response (Extended Data Fig. 4c). Overall, the relative distribution of affected cell types across mutant lines remains consistent with the original analysis when using this more stringent downsampling approach. Accordingly, we have removed the abovementioned statement and replaced panel D of Extended Data Fig. 3, which previously illustrated a non-linear relationship between the number of sequenced cells and DEGs, with the new downsampling-based approach (Extended Data Fig. 4c).

2. The reduced ability to detect low abundance genes is noted in the limitations. However, I am still concerned about the related issue of how the reduced power to detect differential expression among low expressed (but detected) genes would affect the ontology enrichments (that constitute a major portion of the manuscript).
This issue is not unique to this study (which certainly meets or exceeds analysis standards in the field in many areas), but my guess is that significant quantitative differentially expressed genes are skewed towards higher abundance. Because higher abundance genes likely belong to

different ontology categories than the background set of all detected genes, this skew could have strong effects on enrichment results. As such, the authors consider a background set and/or permutation test that accounts for the distribution of expression levels at which DE genes are discovered. I mention this in part because the ontology enrichments are such a major piece of this manuscript.

Response: We agree that expression-dependent read counts reduce the power to detect differential expression for lowly expressed genes, which can bias enrichment. Our ontology analyses use rank-based GSEA applied to all genes that pass our expression filters, which reduces list-selection bias relative to over-representation tests that rely on a DE subset. However, we recognize that GSEA does not fully eliminate abundance-related effects because statistics for lowly expressed genes are inherently noisier. To our knowledge, there is no widely adopted, GSEA-specific correction that directly adjusts for expression-dependent detection power. Methods such as GOseq address this issue for over-representation analyses (not GSEA) and require a DE-gene list, which would further depend on detection power. We have therefore retained GSEA as our primary approach and explicitly acknowledge this limitation in the manuscript.

I do appreciate that in a few cases, the ATAC data provides independent support by showing similar enrichments without being as affected by variation in expression levels. However, it is not clear how enrichments are performed for the ATAC data. Are these enrichments simply done at the gene level for genes linked to differentially accessible peaks? If so, couldn't this introduce a bias, because longer genes and genes with longer intergenic distances are more likely to have a linked differentially accessible peak?

Response: This is an excellent point, and we thank the reviewer for this critical evaluation of our methodology. We performed two types of enrichment analysis, and we have clarified in the Material & Methods section how we control for potential biases like gene length.
Motif Enrichment Analysis: This analysis was performed directly on the DNA sequences of the differentially accessible peaks themselves, not on linked genes. We used the SnapATAC2 package to identify enriched transcription factor motifs within the peak regions. Because this method analyzes the peaks directly, it is completely independent of and not biased by the length or proximity of any associated genes.
GO Term Enrichment Analysis: This analysis was indeed performed on genes associated with differential accessibility at the gene-body level. To directly address the valid concern about potential gene length bias, we performed a post-hoc analysis (Revision Fig. R2.3). We tested for a correlation between the gene-body differential accessibility *p*-values and the corresponding gene lengths. Our analysis revealed no significant association, demonstrating that gene length is not a systematic confounding factor:

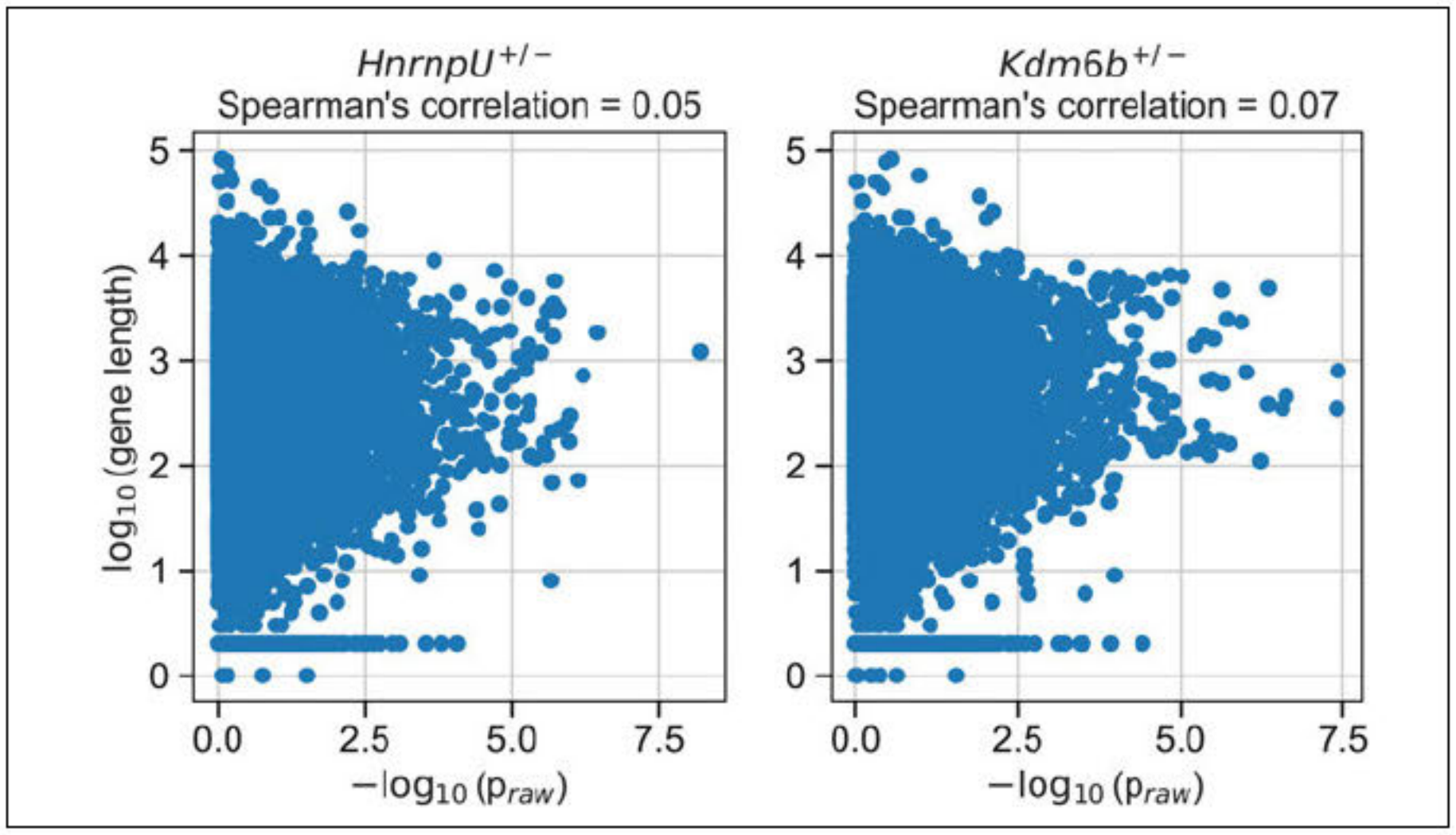

Revision Fig. R2.3: Correlation between gene-body differential accessibility p-values and corresponding gene lengths. Our analysis revealed no significant association, demonstrating that gene length is not a systematic confounding factor.

3. Ambient RNA contamination has been shown to be a major issue in single nuclei data sets (https://doi.org/10.1016/j.neuron.2022.09.010), especially when the phenotype detected relate to glial cell types, since they are the ones primarily contaminated with ambient RNA. The experimental design to collect data in a common batch helps to minimize confounds between ambient RNA and particular genotypes. Nevertheless, the authors could consider tools like Cellbender (https://doi.org/10.1038/s41592-023-01943-7) to clean up nuclear ambient RNA contamination, and they should plot the intronic reads ratio (against UMI bins) to show that none of the analyzed cell types have a low intronic reads ratio, indicator of non-nuclear ambient RNA contamination, all of this prior to plotting the data and drawing conclusions.

[decontX https://genomebiology.biomedcentral.com/articles/10.1186/s13059-020-1950-6, soupx]

We appreciate the reviewer's suggestions and have carried out the recommended analyses. We quantified intronic ratios across all samples and cell types, finding uniformly high and consistent values (Revision Fig. R2.4). For clarity, we present an overview per multiplex rather than per-sample panels, which would be visually overcrowded (with over 200 samples).

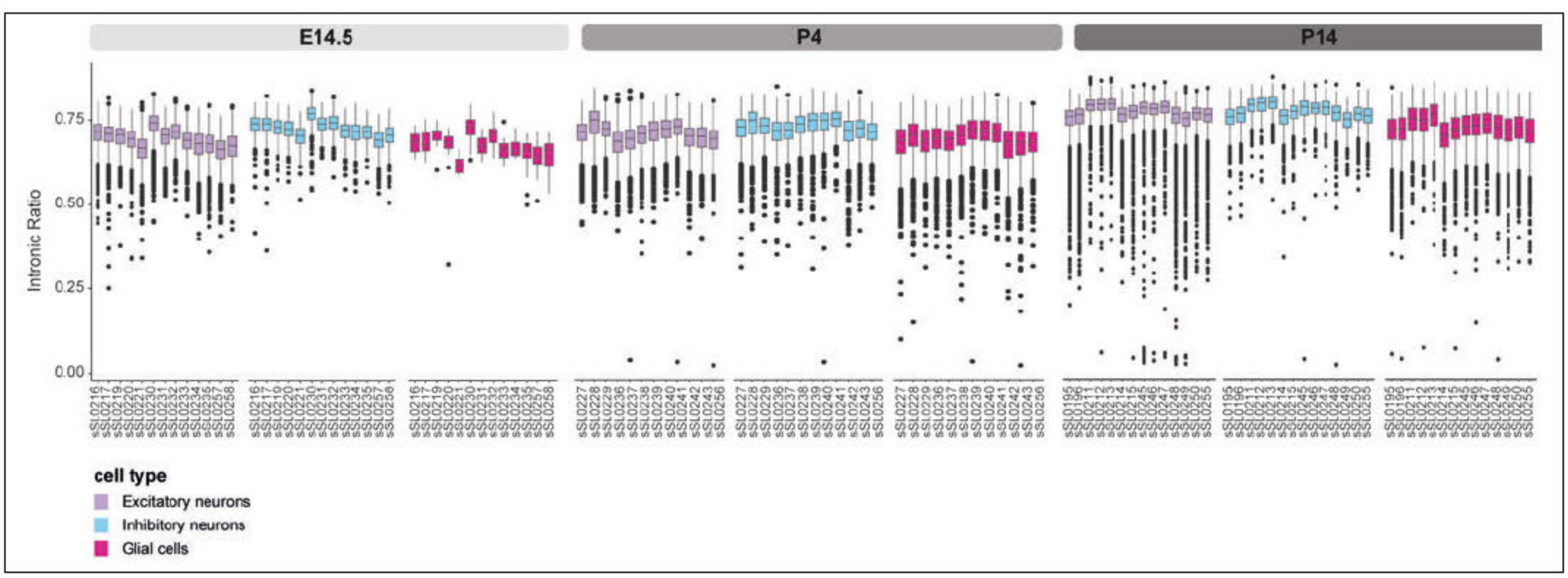

Revision Fig. R2.4: Quality metrics of Dataset A. Intronic read ratios of excitatory and inhibitory Neurons, as well as glial cells across multiplexes and time points. Center lines represent medians, the box limits show the 25-75% interquartile range (IQR), whiskers extend to 1.5 the range of the IQR. Data are mean ± SEM.

Nevertheless, we also repeated our analysis after applying SoupX filtering and found no substantial differences in either the number or identity of DEGs. Together, these results suggest that we did not experience notable ambient RNA contamination.

4. The authors should provide more clear discussion/visuals on the number of nuclei analyzed (post-filtering) for each mutant and sex alone (not just the total number) as this would help assess if sufficient cell coverage has been achieved across conditions and also to add transparency to interpretations of cell abundance differences (like in fig1.e).

Response: We apologize for the lack of clarity in this regard. In the revised manuscript, we have updated Extended Data Fig. 2 to include visualizations for all samples and replicates, ensuring that the distribution of genotypes across clusters is fully represented. To facilitate comparison, we added a stacked bar plot summarizing the representation of genotypes within each cluster. Additionally, we have included the number of nuclei analyzed per cell type in Supplementary Table S1.
Finally, we would like to highlight that a graphical user interface providing online access to the dataset will be publicly accessible upon acceptance of the manuscript. This interface enables filtering and visualization of distributions across multiple parameters (e.g., cell type, sample, sex, etc.).

Minor points:

1. For the pseudotime analysis, in fig.1h, the authors need to show the UMAPs of the control and mutants side-by-side to be able to visualize the differences in trajectories and to see the difference in RG1/RG2 between control and mutants. Also, please indicate which cells were included in the pseudotime analysis, and the choice of the starting point of the trajectory.

Response: We thank the reviewer for this suggestion. While we believe that displaying UMAPs of all mutants side by side may not effectively illustrate the overall differences between RG1 and RG2 cells, we have now included pseudo-time plots of RG cells in Fig. 1i, showing all mutants in comparison to wild-type controls. This provides a clearer visualization of differences in trajectories and RG1/RG2 populations between conditions.
We selected the RG cluster as the starting point for the pseudo-time trajectory because RG cells are the primary neural progenitors in the developing cortex, giving rise to intermediate progenitors, migrating neurons, and ultimately cortical plate neurons. Their central role at the onset of corticogenesis makes them the most biologically meaningful root for reconstructing these developmental trajectories. To clarify the rationale and which cell populations were included in the pseudo-time analysis, we have added this information to the main text and provided further details in the Materials & Methods section.

2. Some figures are hard to follow for the reader, please find a clearer way to present the data:
fig.1b-c and most UMAPs in the paper: the colors of the clusters and labels are very hard to distinguish and identify, consider drawing circles around the clusters or labels on top of the clusters as an extended figure;

Response: We thank the reviewer for this valuable feedback and apologize for the limited readability of the UMAPs. To improve clarity, we have added numerical labels to the clusters in Fig. 1b-c, and Fig. 6a, making it easier to distinguish and identify individual clusters.

fig.2d: the figure is complicated and not all the pathways need to be present in the figure, consider focusing on the ones highlighted;

Response: We have reworked Fig. 2, emphasizing the relationships across mutant lines, using the output of the Network colocalization analysis.

fig.4b: different dot shapes and colors are overlapping and hard to follow, highlight the ones that are relevant;

Response: We apologize for the difficulty in reading the figure. We have revised Fig. 4b to improve clarity and highlight the relevant data points.

extended fig.7: can barely see the dashed rectangles, make them more noticeable;

Response: Thank you for pointing this out. In the revised version of the manuscript, we have exchanged Extended Fig. 7 with results from the Network-based analysis. Here, we identified clusters of functionally related proteins. For each genotype, we quantified the effect size and significance of its impact on these clusters representing biological processes, generating a network-wide map of biological processes affected by each mutation in both excitatory and inhibitory neuronal lineages (Fig. 2c, Extended Data Fig. 9a). Finally, we compared the similarity of expression effects on shared gene network profiles across genotypes and developmental stages (Fig. 2d, Extended Data Fig. 9b).

extended fig.4: very dense, simplify;

Response: We agree that Extended Data Fig. 4 (now Extended Data Fig. 5) is visually dense. However, we believe it effectively illustrates both genotype-specific distinctions and overlapping processes, providing valuable context for the other analyses. We have therefore retained this figure but revised related process-focused figures (e.g., Fig. 2, Fig. 3, Extended Data Fig. 8, etc.) to improve clarity and aid interpretation of shared and distinct patterns.

extended fig.8d: the colors are extremely faint and cannot see them on top of the UMAPs.

Response: We apologize for the limited visibility of the UMAPs. Considering that we are supplying a graphical user interface to access the dataset online upon acceptance of the manuscript, we removed the respective UMAPs. This interface allows interactive filtering and visualization of distributions across multiple parameters (e.g., cell type, sample, sex, etc.).

3. Fig.6a should be part of extended figures, it does not fit with the goal of fig.6.

Response: Considering that we have added behavioral analyses of *Bckdk* and *Kmt5b* mutant animals, complementing the results shown in Fig. 6, we revised Fig. 6 to focus on the most relevant transcriptional-behavioral changes and moved selected panels to Extended Data Fig. 14.

4. Fig.6c and extended fig.2a-c, the text says that “the samples and genotypes were similarly distributed across clusters”, however the figures do not show all the samples. For simplicity the current figures are ok, but it would be nice to have one figure panel that shows all the samples/replicates, to make sure the replicates are similar, and that not a certain sample/replicate is driving the differences detected. The authors could also provide a stacked bar plot or other

visuals to indicate the representation of genotypes in each cluster. Also in these same figures, the order of the samples does not match the order of the samples' labels for WAC mutant and WT, flip the samples on the heatmap to match the label and have the WT at the bottom. Also, fig.6c only shows males, show both males and females.
To be done

Response: We thank the reviewer for this helpful suggestion and for pointing out the order of the labels. We have revised Extended Data Fig. 2e-f to include all samples and replicates, ensuring that the distribution of genotypes across clusters is fully represented. To facilitate comparison, we added a stacked bar plot summarizing the representation of genotypes within each cluster.

Furthermore, we corrected the order of the samples in the heatmaps so that the WT samples appear at the bottom and the *Wac* mutants at the top, in line with the labeling.

Regarding Fig. 6, we confirm that it includes only male samples, as only males were sequenced in the cerebellum dataset.

5. In fig.1i and j, the authors only show RG1 in each mutant and all mutants, include RG2 as well.

Response: We thank the reviewer for pointing this out. This was indeed a mistake in the figure title; the plot in Fig. 1j already includes all RG cells and does not distinguish between RG1 and RG2 subtypes. We have corrected the title accordingly to avoid confusion. Moreover, we have now included pseudo-time plots of RG cells in Fig. 1i showing all mutants in comparison to wild-type controls. This provides a clearer visualization of differences in trajectories and RG1/RG2 populations between conditions.

6. Some p values are missing from legends and text (ex fig.1j), please include all p values. The fold change cutoff for all differential gene expression is missing, please include in text and legends and justify the choice.

Response: We thank the reviewer for this valuable comment. We have revised all figures and legends to include the exact *p*-values. In cases where different thresholds were applied, these are now clearly indicated in the text, figure legends, and/or respective Materials & Methods sections.

7. The text referring to fig.2d does not mention chromosomal segregation in excitatory neurons at E14.5, but it is the biggest colored cluster and the legend says that colored clusters are mentioned in results. Please add.

Response: We thank the reviewer for bringing this to our attention. We have now updated the text referring to previous Fig. 2d to explicitly mention the chromosomal segregation observed in excitatory neurons at E14.5, ensuring consistency with the figure. While we have revised Fig. 2 according to the Reviewer comments, the processes are still present in the updated figure panels.

8. In extended fig. 7, the authors say "several mutant lines showed deregulation of axogenesis and dendrite morphogenesis-related genes, with the directionality of these changes varying between mutants. DEGs associated with these processes were upregulated in PV+ and Vip+ interneurons in most ASD mutants, while they were downregulated in excitatory Layer II/IV neurons." The authors should also include Sst+ cells as they show high upregulation as well.

Response: We have now updated the text to also mention the Sst⁺ interneurons, which show upregulation of axogenesis- and dendrite morphogenesis-related genes in several ASD mutants.

9. In extended fig.10a, the authors say “When analyzing all mutants collectively, we found a strong correlation in Layer II/IV neurons at P4”, the author should also include the strong correlation at P4 in immature astrocytes.

Response: We thank the reviewer for pointing this out. We have now included the strong correlation observed in immature astrocytes at P4 in the main text of the manuscript as well.

References:

1. Rots, D. *et al.* The clinical and molecular spectrum of the *KDM6B*-related neurodevelopmental disorder. *Am. J. Hum. Genet.* **110**, 963–978 (2023).
2. Sheppard, S. E. *et al.* Mechanism of KMT5B haploinsufficiency in neurodevelopment in humans and mice. *Sci. Adv.* **9**, eade1463 (2023).
3. Deliu, E. *et al.* Haploinsufficiency of the intellectual disability gene SETD5 disturbs developmental gene expression and cognition. *Nat. Neurosci.* **21**, 1717–1727 (2018).
4. Kaeser, P. S., Deng, L., Fan, M. & Südhof, T. C. RIM genes differentially contribute to organizing presynaptic release sites. *Proc. Natl. Acad. Sci.* **109**, 11830–11835 (2012).
5. Wu, S. *et al.* The role of RIM in neurotransmitter release: promotion of synaptic vesicle docking, priming, and fusion. *Front. Neurosci.* **17**, 1123561 (2023).
6. Medrihan, L. *et al.* Synapsin II desynchronizes neurotransmitter release at inhibitory synapses by interacting with presynaptic calcium channels. *Nat. Commun.* **4**, 1512 (2013).
7. Dumont, V. & Lehtonen, S. PACSIN proteins in vivo: Roles in development and physiology. *Acta Physiol.* **234**, e13783 (2022).
8. Widagdo, J., Fang, H., Jang, S. E. & Anggono, V. PACSIN1 regulates the dynamics of AMPA receptor trafficking. *Sci. Rep.* **6**, 31070 (2016).
9. Chung, C., Barylko, B., Leitz, J., Liu, X. & Kavalali, E. T. Acute Dynamin Inhibition Dissects Synaptic Vesicle Recycling Pathways That Drive Spontaneous and Evoked Neurotransmission. *J. Neurosci.* **30**, 1363–1376 (2010).
10. Boumil, R. M. *et al.* A Missense Mutation in a Highly Conserved Alternate Exon of Dynamin-1 Causes Epilepsy in Fitful Mice. *PLoS Genet.* **6**, (2010).
11. Chater, T. E. & Goda, Y. The role of AMPA receptors in postsynaptic mechanisms of synaptic plasticity. *Front. Cell. Neurosci.* **8**, 401 (2014).
12. Newell-Litwa, K., Seong, E., Burmeister, M. & Faundez, V. Neuronal and non-neuronal functions of the AP-3 sorting machinery. *J. Cell Sci.* **120**, 531–541 (2007).
13. Körber, C. *et al.* Modulation of Presynaptic Release Probability by the Vertebrate-Specific Protein Mover. *Neuron* **87**, 521–533 (2015).
14. Deguchi, Y. *et al.* mDia and ROCK Mediate Actin-Dependent Presynaptic Remodeling Regulating Synaptic Efficacy and Anxiety. *Cell Rep.* **17**, 2405–2417 (2016).
15. Li, M. *et al.* Frontiers | Structure, activity and function of the lysine methyltransferase SETD5. *Front. Endocrinol.* **14**, (2023).
16. Moore, S. M. *et al.* Setd5 haploinsufficiency alters neuronal network connectivity and leads to autistic-like behaviors in mice. *Transl. Psychiatry* **9**, 1–12 (2019).

17. Gulati, G. S. *et al.* Single-cell transcriptional diversity is a hallmark of developmental potential. *Science* **367**, 405–411 (2020).
18. Meléndez-Ramírez, C. *et al.* Dynamic landscape of chromatin accessibility and transcriptomic changes during differentiation of human embryonic stem cells into dopaminergic neurons. *Sci. Rep.* **11**, 16977 (2021).

Point-to-point response to reviewers' comments:

Referee #1 (Remarks to the Author):

The authors have nicely addressed a majority of my concerns. One of my main concerns has been partially addressed, but additional genes should validated to substantiate claims of convergence of the RG phenotype

My main technical concern (comment #1) was in 2 parts. Concern #1A was a question about the breeding which the authors have nicely addressed. The additional details were very helpful. Concern #1B was a more general one that the evidence for convergence relies on comparing all mutants to a single small control group of 3 wild type males and 3 females. A random statistical fluctuation in the controls will be indistinguishable from a convergent effect of multiple gene mutations. The authors have partly addressed this concern by validating a main finding (increased RG numbers) in a 2nd set of HnrnpU mutant and sibling controls. This response is encouraging. There is reproducible evidence that RG cell numbers are increased in some mutations. The finding replicates in 1 gene (I won't quibble about the p-value of 0.08, but again statistical power of the small Ns is a persistent issue). Also, in the original manuscript differentially expressed genes were validated in several mutants and littermate controls, which is a convincing type of validation. Comparison of all mutants to a single small control group is STILL the basis for the claims of the convergent RG phenotype. To be honest a more convincing result would be one where the effects on RG differ between genes, which would suggest that the pattern is clearly related to the effects of gene mutations.

I recognize that this study was a huge amount of work. And to address this issue would require an even greater amount of work. At a minimum, I think the authors need to acknowledge that there is are key limitations in their experimental design.

1. Mouse studies done on this scale are very costly. Cost saving measures are required including (A) using small Ns per genotype which limits power to detect effects. Thus statistical evidence in Figure 1 is generally modest. Also (b) a small N of common controls has an additional limitation…

Response: We have now explicitly acknowledged in the "Limitations" the statistical power limitations and the constraints associated with shared control groups that are inherent to studies of this scale.

2. Random statistical variability in controls will produce patterns that will not look like noise. It will look like convergence.

This design necessitates a fair amount of validation to substantiate claims of convergence. To make the evidence convincing, they might try expanding their validation to 2 more genes (such as KDM6B, Cul3 which had effects that were similarly strong)… similar to what was done to validate DE genes. Larger Ns of mutant and littermate controls are recommended. Given modest effects that replicated for HNRNPU, Ns of ~15 per group might be needed.

Response: We thank the reviewer for this suggestion. We agree that additional validation across multiple genotypes would further strengthen the evidence for convergence and consider this an important direction for future work.

At the same time, extending the validation to several genotypes with the suggested sample sizes would require substantial additional time and effort. Timed matings and pregnancies are inherently time-dependent processes. Mating success, pregnancy rates, and litter sizes are variable, particularly in mutant lines. In addition, the method used in this manuscript to quantify proliferating cells (tissue clearing followed by volumetric imaging and cell counting) is itself time-intensive, with the clearing step alone requiring approximately one week per experiment.

To address this point as thoroughly as possible within the revision period, we initiated a large number of timed matings across four genotypes (*Cul3*, *Kdm6b*, *HnrnpU*, and *Ptchd1*). In parallel, we

established a cytometry-based protocol to enable more rapid quantification and reduce reliance on the clearing and volumetric imaging workflow.
Despite these efforts, the number of successful pregnancies, the resulting litter sizes, and the availability of litters containing both mutant and wild-type animals within the revision period were insufficient to reach the recommended group sizes (~15 animals per genotype) for several lines. Nevertheless, we achieved the target sample size for *Kdm6b* mutants and completed the requested quantification using the cytometry approach. Consistent with the snRNA-seq data, *Kdm6b* mutants showed a higher proportion of EdU-positive cells compared to wild-type littermate controls. These data have been added to Extended Data Figure 3 (Extended Data Figure 3f-f').

Referee #2 (Remarks to the Author):

I applaud the authors for the detailed revisions and for addressing all major comments.

In particular, the conclusions are strengthened by:
-The more sophisticated analyses of convergence and the consideration of independent control cells in these analyses
-The histological validation of delayed differentiation and altered proliferation
-The correlation between transcriptomic changes and subtle electrophysiological alteration.

Overall, this is an important study with a well-reasoned conceptual framework to systematically analyze many loss-of-function ASD-linked genes together in mouse models, and the analyses add to our knowledge about existing genes and illuminate the potential for transient convergence, while providing a model for future studies.

Response: We thank the reviewer for the support and the previous comments that helped us strengthen the manuscript.

Referee #4 (Remarks to the Author):

I co-reviewed this manuscript with one of the reviewers who provided the listed reports.

Response: We thank the reviewer for the effort in reviewing our manuscript and the comments that helped us strengthen the study.
